# Analysis of the Diversity of *Xylophilus ampelinus* Strains Held in CIRM-CFBP Reveals a Strongly Homogenous Species

**DOI:** 10.3390/microorganisms10081531

**Published:** 2022-07-28

**Authors:** Perrine Portier, Géraldine Taghouti, Paul-Emile Bertrand, Martial Briand, Cécile Dutrieux, Audrey Lathus, Marion Fischer-Le Saux

**Affiliations:** Institut Agro, University Angers, INRAE, IRHS, SFR QUASAV, CIRM-CFBP, F-49000 Angers, France; geraldine.taghouti@inrae.fr (G.T.); bertrand.paul.emile@gmail.com (P.-E.B.); martial.briand@inrae.fr (M.B.); cecile.dutrieux@inrae.fr (C.D.); audrey.lathus@inrae.fr (A.L.); marion.le-saux@inrae.fr (M.F.-L.S.)

**Keywords:** *Xylophilus*, diversity, biological resources center, multi locus sequence analysis

## Abstract

*Xylophilus ampelinus* is the causal agent of blight and canker on grapevine. Only a few data are available on this species implying that the occurrence of this pathogen may be underestimated, and its actual ecological niche may not be understood. Moreover, its genetic diversity is not well known. To improve our knowledge of this species, an analysis of the complete genome sequences available in NCBI was performed. It appeared that several sequences are misidentified. The complete genome sequence of the type strain was obtained and primers designed in order to sequence *gyrB* and *rpoD* genes for the strains held in CIRM-CFBP. The genetic barcoding data were obtained for 93 strains, isolated over 35 years and from several geographical origins. The species revealed to be strongly homogenous, displaying nearly identical sequences for all strains. However, the oldest strains of this collection were isolated in 2001 therefore, a new isolation campaign and epidemiological surveys are necessary, along with the obtention of new complete genome sequences for this species.

## 1. Introduction

*Xylophilus ampelinus* is a Gram-negative betaproteobacterium [1,2] which causes blight and canker on grapevine (*Vitis vinifera*), its only known host. The disease was described in Greece in 1939 but its causal agent was only identified as the slow growing bacteria *Xanthomonas ampelina* in 1969 [3]. This bacterium was also shown to be responsible of different grapevine diseases such as ‘mal nero della vite’ in Italy [4], ‘maladie d’Oléron’ in France [5], ‘vlamsiekte’ in South Africa [6] and ‘necrosis bacteriana’ in Spain [7]. Severity of the disease appears to be dependent on cultivar and strain [8] and can lead to serious harvest losses [9,10]. A DNA and RNA study revealed that this bacterium is not related to *Xanthomonas* and was thus transferred in the *Xylophilus* genus as *X. ampelinus* [11]. This genus is, to date, composed of only two species *X. ampelinus* and “*X. rhododendri*”; the latter is not yet validated [12].

In Europe, *X. ampelinus* was classified as a quarantine organism until 2019 (date of the revision of the list of quarantine organisms), but is still present on the A2 list of organisms established by the European Plant Protection Organization (https://www.eppo.int/, (accessed on 26 July 2022)), indicating that it is still considered as a potential threat for the European and Mediterranean agriculture. The control of the disease can be obtained by using preventive measures such as disinfection of pruning tools, detection and identification of the bacterium to ensure the use of pathogen-free propagative and planting material. Hot water treatment of canes, at 52 °C for 45 min, was shown to eliminate *X. ampelinus* efficiently in grapevine cuttings, along with being efficient toward other pathogens [13,14,15]. More recently, some extracts of the plant *Limonium binervosum* (G.E.Sm.) C.E.Salmon (rock sea-lavender), have shown some activity against *X. ampelinus,* and this could lead to new control strategies [16]. 

This bacterium is distributed in several grapevine-growing areas, such as the Mediterranean basin (France, Greece Italy, Jordan, Moldova, Slovenia), South Africa, Russia and Japan. Reports of symptoms close to the diseases described as caused by *X. ampelinus* have been made from Argentina, Portugal, Switzerland, Tunisia, Turkey and former Yugoslavia, but the presence of the bacterium had not been confirmed (except for Slovenia). Formerly present in Spain, the disease is reported as no longer found since the 2010s. As the occurrence of the disease over the years can be erratic, the symptoms can be confused with other diseases and because of the absence of systematic surveys in many areas, there is uncertainty about its geographical distribution. *X. ampelinus* may be present in more grapevine-growing countries than is currently known [15]. Moreover, the distribution of the bacterium inside the plant can be heterogenous, adding to the difficulties for its detection [17].

It may be possible that the actual ecological niche of the bacterium is not completely known. During the analysis of the American Gut Project, Perz et al. [18] remarked that the microbiota associated to autistic patients are enriched in *Xylophilus ampelinus*. In the MetaMetaDB [19], hits corresponding to *Xylophilus ampelinus* 16S (97% identity) appear in a variety of ecosystems (beetle: 22.46%, soil: 17.67%, rhizosphere: 13.01%, marine: 8.34%, freshwater: 6.98%, root: 6.88%, human lung: 5.79%, ant fungus garden: 5.72%, human skin: 5.08%, hydrocarbon: 4.53% bovine gut: 3.52%). The bacterium has also been recently isolated from the microbiota of blueberry [20], indicating that its actual occurrence in the environment is probably underestimated. 

Only little information is available through public databases; hence, the genetic diversity of this bacterium is poorly known. In this regard, Komatsu et al. [21] established, using Eric-, Box- and Rep-PCR, that the population of the bacterium is homogenous even if they were able to discriminate three genetic types. In GenBank, only thirteen genomic data are available for *Xylophilus*. The complete genome sequence is available for three strains labeled as *X. ampelinus* (including the type strain CECT 7646^T^), two others are available for isolates labeled as *Xylophilus* sp. along with the type strain of ‘*X. rhododendri’* (KACC 21265). Seven other sequences, corresponding to uncultured organisms retrieved from metagenomes, are labeled as *Xylophilus* sp. (https://www.ncbi.nlm.nih.gov/datasets/genomes/?taxon=54066, (accessed on 26 July 2022)). 

The French Collection for Plant-associated Bacteria (CIRM-CFBP; https://cirm-cfbp.fr, (accessed on 26 July 2022)) preserves bacterial resources strategic for plant health, mainly plant-pathogens. These resources serve as a tool available for worldwide researchers, to improve crop health and to better understand plant–bacteria interactions. CIRM-CFBP holds 101 strains of *Xylophilus ampelinus*, isolated from various locations over a long time period. In order to enhance the quality of the strains held in CIRM-CFBP, we decided to obtain the partial sequence of two housekeeping genes for all accessions of the collection. This technique allows to accurately identify the strains at the species level. Moreover, the data can also be used to build the phylogeny of the strains and to better understand the diversity of the considered taxa. This technique was successfully applied in different genera and the protocols (and associated references) used at CIRM-CFBP are available via the collection’s website (https://cirm-cfbp.fr/page/molecular_identification, (accessed on 26 July 2022)). In order to apply this technique to *Xylophilus ampelinus* strains, we sequenced the complete genome of the type strain CFBP 1192^T^ and designed primers for *gyrB* and *rpoD* genes. These two genes were chosen because they are used for the molecular identification of *Xanthomonas* [22] and *Pseudomonas* [23,24] and revealed to be efficient for species identification and diversity analysis of these two genera. The sequences of these two genes were obtained for all the strains held in the collection. In order to complete our study of the diversity of this genus, we also analyzed the different whole genome sequences available in GenBank labeled as *Xylophilus*.

## 2. Materials and Methods

### 2.1. Bacterial Strains

The 101 strains belonging to *Xylophilus ampelinus* held in CIRM-CFBP were isolated all from grapevine plants, over a period of 35 years in Greece, France, Spain and South Africa. The most recent strains present in the collection were isolated in 2001. The strains are preserved as freeze-dried or in sterile water with 40% glycerol at −80 °C or in liquid nitrogen at −196°C. For routine cultivation, the strains are plated on YPGA (yeast extract 7 g.L^−1^; bacto peptone 7 g.L^−1^; glucose 7 g.L^−1^; agar 15 g.L^−1^) for 4 days at 25 °C. The type strain of *Acidovorax anthurii* CFBP 3232^T^ was added in this study as an outgroup. Both species *X. ampelinus* and *A. anthurii* belong to the *Comamonadaceae* family. All strains information is listed in the Supplementary Materials, Table S1. All strains listed in Table S1 are preserved at CIRM-CFBP (https://cirm-cfbp.fr, (accessed on 26 July 2022)) and are available upon request for the international scientific community.

### 2.2. Genome Sequencing

The complete genome sequence of the type strain CFBP 1192^T^ was obtained as described by Merda et al. [25], using the Illumina technology and HiSeq 2000 (Genoscreen, Lille, France). Libraries of genomic DNA were performed using the Kit NextEra 141 XT (Illumina, San Diego, CA, USA). Paired-end reads of 2 × 100 bp were assembled in contigs using SOAPDENOVO 1.05 [26] and VELVET 1.2.02 [27]. Annotation was performed using Prokka [28]. 

### 2.3. Comparative Genomics

The thirteen genomes labeled as *Xylophilus* available in GenBank on 1 June 2022 were retrieved. Six of these genomes correspond to isolates, the other seven sequences correspond to MAGs (Metagenome Assembled Genomes) with no associated cultured isolate. These genome features are listed in Table 1. All genomes were checked for quality using ChekM [29].

The genome sequence data retrieved from GenBank, along with the sequence of CFBP 1192^T^, were uploaded to the Type (Strain) Genome Server (TYGS), a free bioinformatics platform (https://tygs.dsmz.de, (accessed on 26 July 2022)), for a whole genome-based taxonomic analysis [30,31]. The TYGS analysis permits accurate identification, by determining the closest type strains present in the TYGS database, of the uploaded genomes. The Newick tree derived from this analysis was then edited using Mega 11 (https://www.megasoftware.net/, (accessed on 26 July2022)) [32].

The subsequent dDDH (digital DNA-DNA-hybridation) analysis was performed, still by the TYGS pipeline, between the uploaded genomes and a selection of the closest type strains’ genomes from the TYGS database.

After TYGS analysis, ANIb calculation, using pyani [33] were performed with the 14 genomes along with the genome of the type strain of *Xenophilus azovorans* DSM 13620^T^, detected as closely related to the CCH5-B3 and BgEED09 genomes by TYGS analysis.

### 2.4. gyrB-rpoD Phylogeny

Primers to amplify *gyrB* and *rpoD* genes were designed using the genome of the type strain CFBP 1192^T^ (this study) and the sequence of strain CFBP 3232^T^ (*Acidovorax anthurii*; GCA_003269065.1) using the online tool Primer Blast (https://www.ncbi.nlm.nih.gov/tools/primer-blast/, (accessed on 26 July 2022)), and software Amplifix [34] (https://inp.univ-amu.fr/en/amplifx-manage-test-and-design-your-primers-for-pcr, (accessed on 26 July 2022)) and Amplify4 (https://engels.genetics.wisc.edu/amplify/, (accessed on 26 July 2022)). For the 93 strains listed in Table S1, portions of the *gyrB* and *rpoD* genes were sequenced. PCR amplification mix was as follows: Taq polymerase GoTaq (Promega) 5U, polymerase buffer 1X, MgCl_2_ 1 mM, dNTP 100μM, boiled cells 10%. Primers and amplification program are detailed in Table 2.

PCR products’ sequencing was performed by Genoscreen (Lille, France). The consensus sequences for each gene for each strain were extracted from forward and reverse sequence assemblies using Geneious Pro version 9.1.8 (www.geneious.com). The sequences were then aligned and trimmed using BioEdit version 5.0.6. A phylogenetic tree was constructed with concatenated alignments of all genes with MEGA 7.0.26 using the neighbor-joining method with 1000 bootstrap replicates, and the evolutionary distances were computed by using the Kimura two-parameter method. The sequences of *gyrB* and *rpoD* for type strains CFBP 1192^T^ (*X. ampelinus*) and CFBP 3232^T^ (*A. anthurii*) were retrieved from the complete genome sequences, the latter strain acting as an outgroup. The sequences were obtained for both genes for 93 strains. All the sequences used for the phylogenetic tree were deposited at NCBI, and the accession numbers are listed in Table S1. The sequence alignment is provided in Table S2.

## 3. Results and Discussion

### 3.1. Genome Comparison

The genome features for strain CFBP 1192^T^ and NCBI accession number are summarized in Table 3.

The ChekM process revealed, unsurprisingly, that the genomes obtained from MAGs were of a lesser quality than the ones obtained from isolates (Table S3). However, all were uploaded for whole genome analysis by TYGS. 

**Figure 1:**
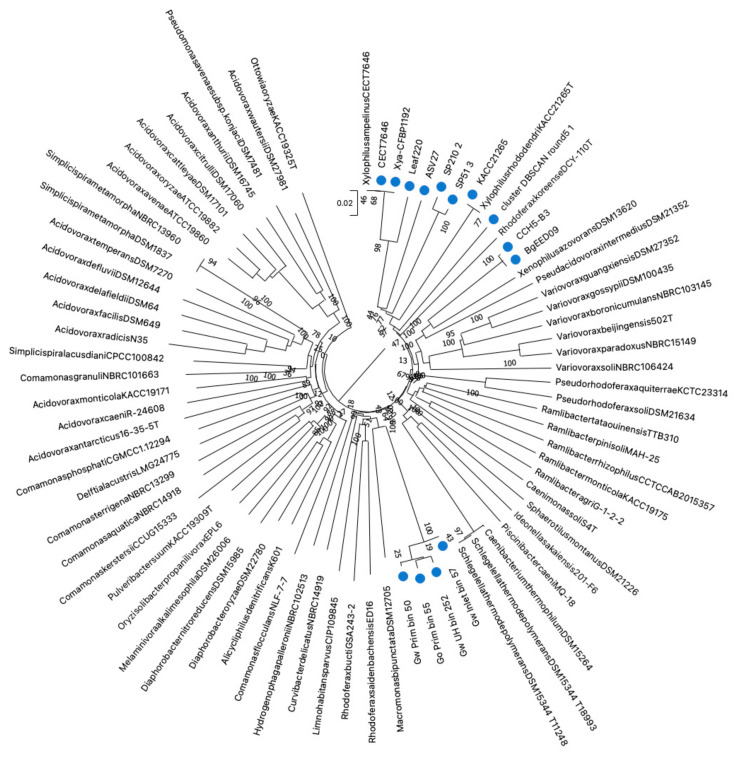
Phylogenetic tree provided after TYGS analysis [30]. Tree inferred with FastME 2.1.6.1 [36] from GBDP distances calculated from genome sequences. The branch lengths are scaled in terms of GBDP distance formula *d_5_*. The numbers on branches are GBDP pseudo-bootstrap support values > 60% from 100 replications, with an average branch support of 81.2%. The tree was rooted at the midpoint [37]. The Newick file was edited in MEGA11 [32]. The 14 blue dots correspond to the uploaded genomes.

The TYGS analysis (Figure 1) revealed that the genomes of the type strains of *X. ampelinus* (CECT 7646^T^, CFBP 1192^T^) and ‘*X. rhododendri*’ (KACC 21265^T^) correspond to their respective taxa. However, all the other genomes labeled as ‘*Xylophilus’* do not correspond to taxa available in the TYGS database. The comparison of the dDDH and ANIb values confirms these results. Strains CECT 7646^T^ and CFBP 1192^T^ display ANIb and dDDH values at 100%, indicating that these two strains are equivalent (Table 4, Figure 1).

**Table 4:** ANIb (above diagonal) and dDDH (below diagonal) values, calculated respectively with pyani [33] and TYSG, formula d_4_ [35]. Highlighted in green, the values above the 95% (for ANIb) or 70% (for dDDH) thresholds for bacterial species delineation. The numbers featured on top, correspond to the genome number on the left. CFBP 1192^T^ and CECT 7646^T^ are both equivalent of the same type strain of the species held in two different collections.

Genome Name	Taxonomy (in Genbank)	1	2	3	4	5	6	7	8	9	10	11	12	13	14	15
**1.** CECT7646	*X. ampelinus* (Type strain)	1.00	1.00	0.93	0.82	0.81	0.81	0.80	0.79	0.78	0.78	0.78	0.77	0.77	0.76	0.76
**2.** Xya-CFBP1192	*X. ampelinus* (Type strain)	1.00	1.00	0.93	0.82	0.81	0.81	0.80	0.79	0.78	0.78	0.78	0.77	0.77	0.77	0.77
**3.** Leaf220	*Xylophilus* sp.	0.48	0.48	1.00	0.25	0.81	0.81	0.23	0.78	0.21	0.22	0.22	0.21	0.21	0.21	0.21
**4.** ASV27	*Xylophilus* sp.	0.25	0.25	0.82	1.00	0.80	0.81	0.23	0.79	0.79	0.79	0.79	0.77	0.77	0.77	0.77
**5.** SP210_2	*Xylophilus* sp.	0.24	0.24	0.24	0.24	1.00	0.98	0.22	0.78	0.21	0.22	0.22	0.20	0.20	0.21	0.20
**6.** SP51_3	*Xylophilus* sp.	0.24	0.24	0.24	0.24	0.86	1.00	0.23	0.78	0.22	0.22	0.22	0.21	0.20	0.22	0.21
**7.** KACC21265	*X. rhododendri* (Type strain)	0.23	0.23	0.79	0.80	0.79	0.79	1.00	0.78	0.77	0.78	0.78	0.76	0.75	0.76	0.76
**8.** cluster_DBSCAN_round5_1	*Xylophilus* sp.	0.22	0.22	0.22	0.22	0.22	0.22	0.21	1.00	0.22	0.22	0.22	0.21	0.20	0.21	0.21
**9.** BgEED09	*Xylophilus ampelinus*	0.22	0.22	0.78	0.22	0.77	0.77	0.21	0.78	1.00	1.00	0.84	0.76	0.76	0.76	0.76
**10.** CCH5-B3	*Xylophilus ampelinus*	0.22	0.22	0.78	0.22	0.78	0.78	0.22	0.78	0.99	1.00	0.83	0.76	0.76	0.76	0.76
**11.** JQKD01.1	*Xenophilus azovorans* DSM 13,620 (Type strain)	0.22	0.22	0.78	0.22	0.78	0.78	0.21	0.78	0.29	0.28	1.00	0.21	0.21	0.21	0.21
**12.** Gw_Inlet_bin_57	*Xylophilus* sp.	0.21	0.21	0.77	0.21	0.76	0.77	0.20	0.76	0.21	0.21	0.77	1.00	0.90	0.97	0.98
**13.** Go_Prim_bin_55	*Xylophilus* sp.	0.21	0.21	0.77	0.21	0.76	0.76	0.20	0.76	0.21	0.21	0.76	0.99	1.00	0.98	0.98
**14.** Gw_Prim_bin_50	*Xylophilus* sp.	0.21	0.21	0.77	0.21	0.76	0.77	0.21	0.76	0.21	0.21	0.77	0.90	0.91	1.00	0.98
**15.** Gw_UH_bin_252	*Xylophilus* sp.	0.20	0.20	0.77	0.20	0.77	0.77	0.20	0.77	0.21	0.21	0.77	0.90	0.89	0.89	1.00

The genomes of strains CCH5-B3 and BgEED09 labeled as *X. ampelinus*, belong to a same species, but are in fact closer to *Xenophilus* strains. The comparison of these two genomes with the genomes of the type strain of *Xenophilus azovorans* added as reference (Table 4), showed that they probably belong to a not yet described species in this genus.

On the other hand, strains ASV27 and leaf220 correspond to two undescribed species embedded inside the *Xylophilus* genus.

The two MAGs SP210_2 and SP51_3 are closely related, belonging to a same species, well embedded in the *Xylophilus* genus, probably corresponding to a not yet described *Xylophilus* species. However, as these genomes were retrieved from MAGs, this assignation may not be accurate enough. The situation is equivalent for the genome cluster_DBSCAN_round5_1 which corresponds to another not yet described species located at the limit of the *Xylophilus* genus. Here also, the limited quality of the genome does not permit to ensure its precise taxonomic position. Finally, the cluster of MAGs retrieved from rice microbiota all belong to a not yet described species close to *Macromonas bipunctata*, but with the same reserves considering the quality of the genomic sequences. Even though the exact taxonomic position of these genomes may not be precise enough, it is sufficient to confirm that microbiotas can contain yet unknown members of *Xylophilus*, and that not all sequences assigned as *Xylophilus* are bona fide *Xylophilus*.

Finally, only two genome sequences belong to *X. ampelinus*, and both were obtained from the type strain (from two different collections). These data are far from enough to permit a comprehensive study of the diversity of this species.

These results indicate two things. The first is that the *Xylophilus* genus is far from well known, with unknown species detected in this genus, with unknown ecological niches and only a few data available. Secondly, that the taxonomic assignation of the publicly available sequences is not always accurate. Hence, this raises the question of the accuracy of the assignation of the sequences extracted from metagenomes and identified as *Xylophilus*. A more in-depth analysis is warranted to determine if they really correspond to *Xylophilus* or to other related genera.

### 3.2. Genetic Diversity of CIRM-CFBP Xylophilus Ampelinus Strains

**Figure 2:**
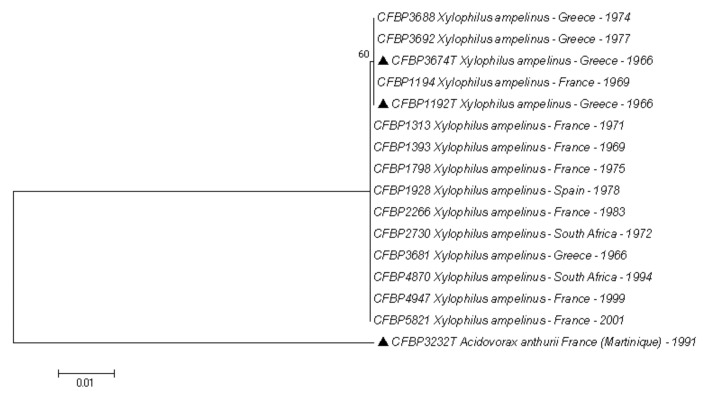
Phylogenetic tree reconstructed from concatenated partial sequences of *gyrB* and *rpoD* housekeeping genes for 15 strains of *Xylophilus ampelinus* and the type strain of *Acidovorax anthurii* as outgroup. The phylogenetic tree was reconstructed with concatenated alignments of all genes with MEGA 7.0.26 using the neighbor-joining method with 1000 bootstrap replicates, and the evolutionary distances were computed by using the Kimura two-parameter method. Triangles indicate the two CFBP accession corresponding ot the type strain (accession duplicated in the CIRM-CFBP collection). The phylogenetic tree of the 93 *Xylophilus ampelinus* strains and all accession numbers of the sequences are available in Figure S1 and Table S1, respectively.

The *gyrB* and *rpoD* sequences were perfectly identical for 1382 base pairs (out of 1383) (Figure 2). The accession numbers for all *gyrB* and *rpoD* sequences are available in Table S1, the alignment of the sequences is available in Table S2, a version of the phylogenetic tree including the 93 *X. ampelinus* strains is available in Figure S1. A single 1 base-pair difference was observed in the *rpoD* sequence for 18 of the 92 strains, including the type strain of the species. A third gene (*rpoB*, results not shown) had been tested for a few strains leading also to perfectly identical sequences (thus, the analysis with this gene was not completed). These results are surprising considering that the strains have been isolated over a period of 35 years from different countries: Spain, Greece, France and South Africa. The number of analyzed genes is limited and may not reflect the actual diversity of the species. However, for other genera of plant-pathogenic bacteria, the analysis of only a few (1–3) housekeeping genes is enough to reveal the genetic diversity of the considered taxa. It is the case for *Xanthomonas* [38], *Acidovorax* [39] or *Pectobacterium* [40] for instance. A complete MultiLocus Sequence Analysis study of *Curtobacterium* flaccumfaciens [41] used 6 loci, but each locus independently was enough to reveal the diversity of the species. The homogeneity of *X. ampelinus* is thus remarkable.

In 2016, Komatsu et al. [21] described a limited genetic variability in *X. ampelinus* strains revealed by a combination of Box-, Eric- and Rep-PCR. The strains were divided in 4 groups, groups A and B comprising CFBP strains. The comparison of these results with the ones of the present study showed no correlations. The group A described by Komatsu et al. [21] clusters together strains belonging to both groups revealed by our study of *gyrB* and *rpoD* sequences. These different techniques do not analyze the diversity at the same level. Sequencing of housekeeping genes provide reliable information at the species/intra-specific level, while the Box-, Eric- and Rep-PCR are able to assess variations between individual strains. Thus, these two findings can be compatible. The analysis of a larger number of genomes of strains actually belonging to *X. ampelinus* is needed to bring a definitive answer on the actual diversity of this species.

Our results suggest that the species is very homogenous considering the housekeeping genes, with a limited diversity existing between the different strains. Grall et al. [17], reported that sap and old wood are the main reservoirs for the bacterium. Hence, human activities such as pruning, grafting and plant cuttings’ transportation are highly susceptible to favorize the spread of the bacterium. If this bacterium is disseminated by human activities from plant to plant, this could explain the homogenic structure of the species.

## 4. Conclusions

The homogeneity of *X. ampelinus* species is a key fact for plant pathology, permitting to better choose how to design tools for detection and identification of this species. However, more data on the diversity of the strains belonging to this species is necessary. Moreover, the analyzed collection does not extend further to strains isolated in 2001. Even though the analyzed strains are numerous, from diverse locations, and isolated at different times, these findings must be confirmed by the analysis of more recent strains. Hence, new isolation campaign and epidemiological surveys are necessary. As highlighted by Broders et al. [42], the continuous isolation and reliable preservation of plant-pathogenic strains is beneficial in the long term and can be of crucial help when epidemics arise.

On the other hand, the identification of the potential source of spread of a plant-pathogen such as *X. ampelinus* is of crucial importance for plant health. A better knowledge of the reservoirs of inoculum could indicate where and how the efforts should be concentrated to limit the effects of the disease on crops. The analysis of the different genome sequences available in the public databases showed clearly that the ecological niche of the genus *Xylophilus* is largely unknown. Its actual ecological importance, beyond its pathogenicity on grapevine, is still to be described. The ongoing analysis of microbiota in various environments could help us to better understand this genus and its repartition, once the problem of the accuracy of the sequence assignation has been addressed. The better characterization of *Xylophilus* strains held in the collection can help with this task and we encourage scientists to characterize their strains and to make them available for the scientific community.

## Figures and Tables

**Table 1:** Genomes available in GenBank on 1 June 2022 labeled as *Xylophilus*.

Isolate/Genome	Taxonomy	Isolate/MAG	Biotope	Biosample	Bioproject	Assembly	Total Length (bp)	Assembly Level
CECT 7646^T^	*Xylophilus ampelinus*	Isolate	Plant, *Vitis vinifera*	SAMN09074800	PRJNA463320	GCA_003217575.1	3731505	Scaffold
CCH5-B3	*Xylophilus ampelinus*	Isolate	Biofilm, hospital ward	SAMN04299458	PRJNA299404	GCA_001556675.1	6019991	Contig
BgEED09	*Xylophilus ampelinus*	Isolate	Human duodenum	SAMEA5664384	PRJEB32184	GCA_901875635.1	6174221	Contig
KACC 21265	*Xylophilus rhododendri*	Isolate	Plant, *Rhododendron schlippenbachii*	SAMN13783577	PRJNA600143	GCA_009906855.1	5873400	Complete Genome
ASV27	*Xylophilus* sp.	Isolate	Plant, *Sarracenia purpurea*	SAMN17004937	PRJNA224116	GCA_016428875.1	4734944	Contig
leaf220	*Xylophilus* sp.	Isolate	Plant, *Arabidopsis thaliana*	SAMN04151686	PRJNA297956	GCA_001421705.1	4483623	Scaffold
Gw_UH_bin_252	*Xylophilus* sp.	MAG	Wastewater treatment	SAMN18119505	PRJNA524094	GCA_017989255.1	1400660	Scaffold
Go_Prim_bin_55	*Xylophilus* sp.	MAG	Wastewater treatment	SAMN18119707	PRJNA524094	GCA_017990095.1	2320559	Scaffold
Gw_Prim_bin_50	*Xylophilus* sp.	MAG	Wastewater treatment	SAMN18119294	PRJNA524094	GCA_018005875.1	1282324	Scaffold
Gw_Inlet_bin_57	*Xylophilus* sp.	MAG	Wastewater treatment	SAMN18119261	PRJNA524094	GCA_018006615.1	1897017	Scaffold
SP210_2	*Xylophilus* sp.	MAG	Plant, rice	SAMEA8944525	PRJEB45634	GCA_913776965.1	4051675	Contig
SP51_3	*Xylophilus* sp.	MAG	Plant, rice	SAMEA8944104	PRJEB45634	GCA_913777525.1	3038960	Contig
cluster_DBSCAN_round5_1	*Xylophilus* sp.	MAG	Insect, Lagria villosa	SAMN12995593	PRJNA531449	GCA_009914555.1	4706822	Contig

**Table 2:** Primer sequences and PCR programs for partial *gyrB* and *rpoD* amplification for diversity analysis of *Xylophilus ampelinus* strains.

Gene	Primer	Sequence 5’-3’		Expected Size (bp)	Tm
*gyrB*	gyrB_XyF	AGATGGACGACAAGCACGAG		841	60
	gyrB_XyR	TTGGTCTGGCTGCTGAACTT			60
		30X			
95 °C	95 °C	65 °C	72 °C	72 °C	15 °C
5′	30′′	30′′	30′′	5′	∞
Gene	Primer	Sequence 5’-3’		Expected size (bp)	Tm
*rpoD*	rpoD-XyF	AAGGAACGCGCCTTGATGA		767	60
	rpoD-XyR	CCGTAGCCTTCCTTGTCGTAG			60
**PCR Program**					
		30X			
95 °C	95 °C	58 °C	72 °C	72 °C	15 °C
5′	30′′	30′′	30′′	5′	∞

**Table 3:** Features of the complete genome sequence of CFBP 1192^T^, type strain of *Xylophilus ampelinus*.

Strain	Size	Scaffolds	%GC	N50	N50 BP	Coverage	CDS	NCBI Accession
CFBP 1192^T^	3,736,570	85	67.8	9	138.681	225	3307	JAMOFZ000000000

## Data Availability

The sequence data supporting this article can be found in GenBank under the accession numbers listed in Table 2 and Table S1.

## Supplementary Materials

The following supporting information can be downloaded at: https://www.mdpi.com/article/10.3390/microorganisms10081531/s1. Figure S1: phylogenetic tree constructed from *gyrB* and *rpoD* concatenated sequences for the 93 strains of *X. ampelinus* held in CIRM-CFBP, Table S1: information on strains used in this study, Table S2: *gyrB-rpoD* sequence alignment for all strains listed in Table S1, Table S3: Statistics derived from ChekM analysis [29] on the *Xylophilus* genomes retrieved from NCBI.
