# Supplementary material for: Analysis of the Diversity of Xylophilus ampelinus Strains Held in CIRM-CFBP Reveals a Strongly Homogenous Species
Source: Microorganisms. 2022 Jul 28;10(8):1531. doi: 10.3390/microorganisms10081531 (PMC9412579; doi:10.3390/microorganisms10081531)
Supplement: Supplementary file 1 [file microorganisms-10-01531-s001.zip › PPortier-Xylophilus Supplemental data/PPortier-Xylophilus ampelinus-Caption and Table S2 .pdf]

Analysis of the diversity of *Xylophilus ampelinus* strains held in CIRM-CFBP reveals a strongly homogenous species.

Perrine Portier, Géraldine Taghouti, Paul-Emile Bertrand, Martial Briand, Cécile Dutrieux, Audrey Lathus, and Marion Fischer-Le Saux

Table S1: information on strains used in this study

Table S2: *gyrB-rpoD* sequence alignment for all strains listed in table S1. The *gyrB* sequence span from nucleotides 1 to 723 and *rpoD* sequence from nucleotide 724 to 1383.

Table S3: Results of ChekM [1] Analysis run on the 13 genomes of *Xylophilus* downloaded from Genbank along with the genome of CFBP 1192^{T}.
[1]: Parks, D.H.; Imelfort, M.; Skennerton, C.T.; Hugenholtz, P.; Tyson, G.W. CheckM: assessing the quality of microbial genomes recovered from isolates, single cells, and metagenomes. *Genome Res*. **2015** 25(7):1043-55

Figure S1: Phylogenetic tree reconstructed from concatenated partial sequences of *gyrB* and *rpoD* housekeeping genes for 93 strains of *Xylophilus ampelinus* and the type strain of *Acidovorax anthurii* as outgroup. The phylogenetic tree was reconstructed with concatenated alignments of all genes with MEGA 7.0.26 using the neighbour-joining method with 1000 bootstrap replicates, and the evolutionary distances were computed by using the Kimura two-parameter method.

>CFBP1193 Xylophilus ampelinus
CACGCCGGCGGCAAGTTCAACCAGAACAGCTACAAGGTCTCGGGCGGCCTGCACGGCGTG
GGCGTGAGTTGCGTCAACGCGCTCAGCAAGATGCTGCGCCTGACGGTGCGGCGCGACGGC
AAGGTCCACGTGCTGGAGTTCAGCAAGGGCTTCGTGCAGAACCGCATCGTCGAGCAGGCG
GGCGGCGTCGAGGTCTCGCCGATGCGCATCATGGGCGACACCGAGAAGCGCGGTACCGAG
GTGCACTTCCTGCCCGACAACGAGATCTTCAAGGAAAACGCCGATTTCCACTACGAGATC
CTGGCCAAGCGCCTGCGCGAACTGAGCTTCCTGAACAACGGCGTGCGCATCCGCCTGAAG
GACGAGCGCAGCGGCAAGGAAGACGATTTCTCCGGCGCCGGCGGCGTGCAGGGTTTCGTC
GAATTCATCAACAAGGGCAAGACCATCCTGCACCCCACGCCCTTCTACGCCGCGGGCGAG
AAGCCGGCCGAGACGTACGGCGGCATCCCCGGCACGCACATCGGCGTGGAAGTGTCGATG
CAGTGGAACAGCGGCTACACCGAGCAGGTGCTCTGTTTCACCAACAACATCCCGCAGCGC
GACGGCGGCACCCACCTGACCGGCCTGCGCGCCGCGATGACCCGGATCATCAACAAGTAC
ATCGAAGAAAACGAGCTGGCCAAGAAGGCCAAGGTCGAGGTAACCGGCGACGACATGCGC
GAAGAAGAGGACATGGCCAAGCGCCGTTCGCGCCTGAAGGCGTTGATCACGCTCGGCAAG
ACCCGCGGCTACCTGACGCAGGTCGAGATCTCCGACCACCTGCCCGACAAGCTGGTCGAC
GCCGAGACGATGGAAGTCGTGATCGCCATGTTGAACGACATGGGCATCGCGGTCTACGAG
CAGACGCCCGACGCCGAGACGCTGTTCCAGAACAACGTCACCCCCACCGCCGCCACTGTC
GAGGAAGCCGAGGAAGAAGCCGAGGCCGCGCTCTCCACCGTCGACAGCGAATTCGGCCGC
ACCACCGACCCGGTCCGCATGTACATGCGCGAAATGGGCACCGTCGAGCTGCTGACCCGC
GAAGGCGAGATCGAGATCGCCAAGCGCATCGAAGGCGGCATGCAGGCCATGATGGAGGCG
ATCAGCGCGTCGCCCGCCACCATCGCCGCGATCCTGGTGATGGGCGAGGACATCCGCGAA
GGCAAGGTCGTCATCTCGACCATCGTCGACGGCTTCTCCAACCCCAACGAAGCCGACGAC
TACGTGGCCGAGGAGGACTTCGACGAGTTCGACGAGGAAGACGACGACGACGGCAAGGGC
GGCTCCAAGGCGCTGACCAAGAAGCTGGAAGAATTGAAGCGCGAGGCGCTGTCGCGCTTC
GAC
>CFBP1194 Xylophilus ampelinus
CACGCCGGCGGCAAGTTCAACCAGAACAGCTACAAGGTCTCGGGCGGCCTGCACGGCGTG
GGCGTGAGTTGCGTCAACGCGCTCAGCAAGATGCTGCGCCTGACGGTGCGGCGCGACGGC
AAGGTCCACGTGCTGGAGTTCAGCAAGGGCTTCGTGCAGAACCGCATCGTCGAGCAGGCG
GGCGGCGTCGAGGTCTCGCCGATGCGCATCATGGGCGACACCGAGAAGCGCGGTACCGAG
GTGCACTTCCTGCCCGACAACGAGATCTTCAAGGAAAACGCCGATTTCCACTACGAGATC
CTGGCCAAGCGCCTGCGCGAACTGAGCTTCCTGAACAACGGCGTGCGCATCCGCCTGAAG
GACGAGCGCAGCGGCAAGGAAGACGATTTCTCCGGCGCCGGCGGCGTGCAGGGTTTCGTC
GAATTCATCAACAAGGGCAAGACCATCCTGCACCCCACGCCCTTCTACGCCGCGGGCGAG
AAGCCGGCCGAGACGTACGGCGGCATCCCCGGCACGCACATCGGCGTGGAAGTGTCGATG
CAGTGGAACAGCGGCTACACCGAGCAGGTGCTCTGTTTCACCAACAACATCCCGCAGCGC
GACGGCGGCACCCACCTGACCGGCCTGCGCGCCGCGATGACCCGGATCATCAACAAGTAC
ATCGAAGAAAACGAGCTGGCCAAGAAGGCCAAGGTCGAGGTAACCGGCGACGACATGCGC
GAAGAAGAGGACATGGCCAAGCGCCGTTCGCGCCTGAAGGCGTTGATCACGCTCGGCAAG
ACCCGCGGCTACCTGACGCAGGTCGAGATCTCCGACCACCTGCCCGACAAGCTGGTCGAC
GCCGAGACGATGGAAGTCGTGATCGCCATGTTGAACGACATGGGCATCGCGGTCTACGAG
CAGACGCCCGACGCCGAGACGCTGTTCCAGAACAACGTCACCCCCACCGCCGCCACTGTC
GAGGAAGCCGAGGAAGAAGCCGAGGCCGCGCTCTCCACCGTCGACAGCGAATTCGGCCGC
ACCACCGACCCGGTCCGCATGTACATGCGCGAAATGGGCACCGTCGAGCTGCTGACCCGC
GAAGGCGAGATCGAGATCGCCAAGCGCATCGAAGGCGGCATGCAGGCCATGATGGAGGCG
ATCAGCGCGTCGCCCGCCACCATCGCCGCGATCCTGGTGATGGGCGAGGACATCCGCGAA
GGCAAGGTCGTCATCTCGACCATCGTCGACGGCTTCTCCAACCCCAACGAAGCCGACGAC
TACGTGGCCGAGGAGGACTTCGACGAGTTCGACGAGGAAGACGACGACGACGGCAAGGGC
GGCTCCAAGGCGCTGACCAAGAAGCTGGAAGAATTGAAGCGCGAGGCGCTGTCGCGCTTC
GAC
>CFBP1313 Xylophilus ampelinus
CACGCCGGCGGCAAGTTCAACCAGAACAGCTACAAGGTCTCGGGCGGCCTGCACGGCGTG
GGCGTGAGTTGCGTCAACGCGCTCAGCAAGATGCTGCGCCTGACGGTGCGGCGCGACGGC
AAGGTCCACGTGCTGGAGTTCAGCAAGGGCTTCGTGCAGAACCGCATCGTCGAGCAGGCG
GGCGGCGTCGAGGTCTCGCCGATGCGCATCATGGGCGACACCGAGAAGCGCGGTACCGAG
GTGCACTTCCTGCCCGACAACGAGATCTTCAAGGAAAACGCCGATTTCCACTACGAGATC
CTGGCCAAGCGCCTGCGCGAACTGAGCTTCCTGAACAACGGCGTGCGCATCCGCCTGAAG
GACGAGCGCAGCGGCAAGGAAGACGATTTCTCCGGCGCCGGCGGCGTGCAGGGTTTCGTC

GAATTCATCAACAAGGGCAAGACCATCCTGCACCCCACGCCCTTCTACGCCGCGGGCGAG
AAGCCGGCCGAGACGTACGGCGGCATCCCCGGCACGCACATCGGCGTGGAAGTGTCGATG
CAGTGGAACAGCGGCTACACCGAGCAGGTGCTCTGTTTCACCAACAACATCCCGCAGCGC
GACGGCGGCACCCACCTGACCGGCCTGCGCGCCGCGATGACCCGGATCATCAACAAGTAC
ATCGAAGAAAACGAGCTGGCCAAGAAGGCCAAGGTCGAGGTAACCGGCGACGACATGCGC
GAAGAAGAGGACATGGCCAAGCGCCGTTCGCGCCTGAAGGCGTTGATCACGCTCGGCAAG
ACCCGCGGCTACCTGACGCAGGTCGAGATCTCCGACCACCTGCCCGACAAGCTGGTCGAC
GCCGAGACGATGGAAGTCGTGATCGCCATGTTGAACGACATGGGCATCGCGGTCTACGAG
CAGACGCCCGACGCCGAGACGCTGTTCCAGAACAACGTCACCCCCACCGCCGCCACTGTC
GAGGAAGCCGAGGAAGAAGCCGAGGCCGCGCTCTCCACCGTCGACAGCGAATTCGGCCGC
ACCACCGACCCGGTCCGCATGTACATGCGCGAAATGGGCACCGTCGAGCTGCTGACCCGC
GAAGGCGAGATCGAGATCGCCAAGCGCATCGAAGGCGGCCTGCAGGCCATGATGGAGGCG
ATCAGCGCGTCGCCCGCCACCATCGCCGCGATCCTGGTGATGGGCGAGGACATCCGCGAA
GGCAAGGTCGTCATCTCGACCATCGTCGACGGCTTCTCCAACCCCAACGAAGCCGACGAC
TACGTGGCCGAGGAGGACTTCGACGAGTTCGACGAGGAAGACGACGACGACGGCAAGGGC
GGCTCCAAGGCGCTGACCAAGAAGCTGGAAGAATTGAAGCGCGAGGCGCTGTCGCGCTTC
GAC
>CFBP1393 Xylophilus ampelinus
CACGCCGGCGGCAAGTTCAACCAGAACAGCTACAAGGTCTCGGGCGGCCTGCACGGCGTG
GGCGTGAGTTGCGTCAACGCGCTCAGCAAGATGCTGCGCCTGACGGTGCGGCGCGACGGC
AAGGTCCACGTGCTGGAGTTCAGCAAGGGCTTCGTGCAGAACCGCATCGTCGAGCAGGCG
GGCGGCGTCGAGGTCTCGCCGATGCGCATCATGGGCGACACCGAGAAGCGCGGTACCGAG
GTGCACTTCCTGCCCGACAACGAGATCTTCAAGGAAAACGCCGATTTCCACTACGAGATC
CTGGCCAAGCGCCTGCGCGAACTGAGCTTCCTGAACAACGGCGTGCGCATCCGCCTGAAG
GACGAGCGCAGCGGCAAGGAAGACGATTTCTCCGGCGCCGGCGGCGTGCAGGGTTTCGTC
GAATTCATCAACAAGGGCAAGACCATCCTGCACCCCACGCCCTTCTACGCCGCGGGCGAG
AAGCCGGCCGAGACGTACGGCGGCATCCCCGGCACGCACATCGGCGTGGAAGTGTCGATG
CAGTGGAACAGCGGCTACACCGAGCAGGTGCTCTGTTTCACCAACAACATCCCGCAGCGC
GACGGCGGCACCCACCTGACCGGCCTGCGCGCCGCGATGACCCGGATCATCAACAAGTAC
ATCGAAGAAAACGAGCTGGCCAAGAAGGCCAAGGTCGAGGTAACCGGCGACGACATGCGC
GAAGAAGAGGACATGGCCAAGCGCCGTTCGCGCCTGAAGGCGTTGATCACGCTCGGCAAG
ACCCGCGGCTACCTGACGCAGGTCGAGATCTCCGACCACCTGCCCGACAAGCTGGTCGAC
GCCGAGACGATGGAAGTCGTGATCGCCATGTTGAACGACATGGGCATCGCGGTCTACGAG
CAGACGCCCGACGCCGAGACGCTGTTCCAGAACAACGTCACCCCCACCGCCGCCACTGTC
GAGGAAGCCGAGGAAGAAGCCGAGGCCGCGCTCTCCACCGTCGACAGCGAATTCGGCCGC
ACCACCGACCCGGTCCGCATGTACATGCGCGAAATGGGCACCGTCGAGCTGCTGACCCGC
GAAGGCGAGATCGAGATCGCCAAGCGCATCGAAGGCGGCCTGCAGGCCATGATGGAGGCG
ATCAGCGCGTCGCCCGCCACCATCGCCGCGATCCTGGTGATGGGCGAGGACATCCGCGAA
GGCAAGGTCGTCATCTCGACCATCGTCGACGGCTTCTCCAACCCCAACGAAGCCGACGAC
TACGTGGCCGAGGAGGACTTCGACGAGTTCGACGAGGAAGACGACGACGACGGCAAGGGC
GGCTCCAAGGCGCTGACCAAGAAGCTGGAAGAATTGAAGCGCGAGGCGCTGTCGCGCTTC
GAC
>CFBP1394 Xylophilus ampelinus
CACGCCGGCGGCAAGTTCAACCAGAACAGCTACAAGGTCTCGGGCGGCCTGCACGGCGTG
GGCGTGAGTTGCGTCAACGCGCTCAGCAAGATGCTGCGCCTGACGGTGCGGCGCGACGGC
AAGGTCCACGTGCTGGAGTTCAGCAAGGGCTTCGTGCAGAACCGCATCGTCGAGCAGGCG
GGCGGCGTCGAGGTCTCGCCGATGCGCATCATGGGCGACACCGAGAAGCGCGGTACCGAG
GTGCACTTCCTGCCCGACAACGAGATCTTCAAGGAAAACGCCGATTTCCACTACGAGATC
CTGGCCAAGCGCCTGCGCGAACTGAGCTTCCTGAACAACGGCGTGCGCATCCGCCTGAAG
GACGAGCGCAGCGGCAAGGAAGACGATTTCTCCGGCGCCGGCGGCGTGCAGGGTTTCGTC
GAATTCATCAACAAGGGCAAGACCATCCTGCACCCCACGCCCTTCTACGCCGCGGGCGAG
AAGCCGGCCGAGACGTACGGCGGCATCCCCGGCACGCACATCGGCGTGGAAGTGTCGATG
CAGTGGAACAGCGGCTACACCGAGCAGGTGCTCTGTTTCACCAACAACATCCCGCAGCGC
GACGGCGGCACCCACCTGACCGGCCTGCGCGCCGCGATGACCCGGATCATCAACAAGTAC
ATCGAAGAAAACGAGCTGGCCAAGAAGGCCAAGGTCGAGGTAACCGGCGACGACATGCGC
GAAGAAGAGGACATGGCCAAGCGCCGTTCGCGCCTGAAGGCGTTGATCACGCTCGGCAAG
ACCCGCGGCTACCTGACGCAGGTCGAGATCTCCGACCACCTGCCCGACAAGCTGGTCGAC
GCCGAGACGATGGAAGTCGTGATCGCCATGTTGAACGACATGGGCATCGCGGTCTACGAG

CAGACGCCCGACGCCGAGACGCTGTTCCAGAACAACGTCACCCCCACCGCCGCCACTGTC
GAGGAAGCCGAGGAAGAAGCCGAGGCCGCGCTCTCCACCGTCGACAGCGAATTCGGCCGC
ACCACCGACCCGGTCCGCATGTACATGCGCGAAATGGGCACCGTCGAGCTGCTGACCCGC
GAAGGCGAGATCGAGATCGCCAAGCGCATCGAAGGCGGCCTGCAGGCCATGATGGAGGCG
ATCAGCGCGTCGCCCGCCACCATCGCCGCGATCCTGGTGATGGGCGAGGACATCCGCGAA
GGCAAGGTCGTCATCTCGACCATCGTCGACGGCTTCTCCAACCCCAACGAAGCCGACGAC
TACGTGGCCGAGGAGGACTTCGACGAGTTCGACGAGGAAGACGACGACGACGGCAAGGGC
GGCTCCAAGGCGCTGACCAAGAAGCTGGAAGAATTGAAGCGCGAGGCGCTGTCGCGCTTC
GAC
>CFBP1796 Xylophilus ampelinus
CACGCCGGCGGCAAGTTCAACCAGAACAGCTACAAGGTCTCGGGCGGCCTGCACGGCGTG
GGCGTGAGTTGCGTCAACGCGCTCAGCAAGATGCTGCGCCTGACGGTGCGGCGCGACGGC
AAGGTCCACGTGCTGGAGTTCAGCAAGGGCTTCGTGCAGAACCGCATCGTCGAGCAGGCG
GGCGGCGTCGAGGTCTCGCCGATGCGCATCATGGGCGACACCGAGAAGCGCGGTACCGAG
GTGCACTTCCTGCCCGACAACGAGATCTTCAAGGAAAACGCCGATTTCCACTACGAGATC
CTGGCCAAGCGCCTGCGCGAACTGAGCTTCCTGAACAACGGCGTGCGCATCCGCCTGAAG
GACGAGCGCAGCGGCAAGGAAGACGATTTCTCCGGCGCCGGCGGCGTGCAGGGTTTCGTC
GAATTCATCAACAAGGGCAAGACCATCCTGCACCCCACGCCCTTCTACGCCGCGGGCGAG
AAGCCGGCCGAGACGTACGGCGGCATCCCCGGCACGCACATCGGCGTGGAAGTGTCGATG
CAGTGGAACAGCGGCTACACCGAGCAGGTGCTCTGTTTCACCAACAACATCCCGCAGCGC
GACGGCGGCACCCACCTGACCGGCCTGCGCGCCGCGATGACCCGGATCATCAACAAGTAC
ATCGAAGAAAACGAGCTGGCCAAGAAGGCCAAGGTCGAGGTAACCGGCGACGACATGCGC
GAAGAAGAGGACATGGCCAAGCGCCGTTCGCGCCTGAAGGCGTTGATCACGCTCGGCAAG
ACCCGCGGCTACCTGACGCAGGTCGAGATCTCCGACCACCTGCCCGACAAGCTGGTCGAC
GCCGAGACGATGGAAGTCGTGATCGCCATGTTGAACGACATGGGCATCGCGGTCTACGAG
CAGACGCCCGACGCCGAGACGCTGTTCCAGAACAACGTCACCCCCACCGCCGCCACTGTC
GAGGAAGCCGAGGAAGAAGCCGAGGCCGCGCTCTCCACCGTCGACAGCGAATTCGGCCGC
ACCACCGACCCGGTCCGCATGTACATGCGCGAAATGGGCACCGTCGAGCTGCTGACCCGC
GAAGGCGAGATCGAGATCGCCAAGCGCATCGAAGGCGGCCTGCAGGCCATGATGGAGGCG
ATCAGCGCGTCGCCCGCCACCATCGCCGCGATCCTGGTGATGGGCGAGGACATCCGCGAA
GGCAAGGTCGTCATCTCGACCATCGTCGACGGCTTCTCCAACCCCAACGAAGCCGACGAC
TACGTGGCCGAGGAGGACTTCGACGAGTTCGACGAGGAAGACGACGACGACGGCAAGGGC
GGCTCCAAGGCGCTGACCAAGAAGCTGGAAGAATTGAAGCGCGAGGCGCTGTCGCGCTTC
GAC
>CFBP1797 Xylophilus ampelinus
CACGCCGGCGGCAAGTTCAACCAGAACAGCTACAAGGTCTCGGGCGGCCTGCACGGCGTG
GGCGTGAGTTGCGTCAACGCGCTCAGCAAGATGCTGCGCCTGACGGTGCGGCGCGACGGC
AAGGTCCACGTGCTGGAGTTCAGCAAGGGCTTCGTGCAGAACCGCATCGTCGAGCAGGCG
GGCGGCGTCGAGGTCTCGCCGATGCGCATCATGGGCGACACCGAGAAGCGCGGTACCGAG
GTGCACTTCCTGCCCGACAACGAGATCTTCAAGGAAAACGCCGATTTCCACTACGAGATC
CTGGCCAAGCGCCTGCGCGAACTGAGCTTCCTGAACAACGGCGTGCGCATCCGCCTGAAG
GACGAGCGCAGCGGCAAGGAAGACGATTTCTCCGGCGCCGGCGGCGTGCAGGGTTTCGTC
GAATTCATCAACAAGGGCAAGACCATCCTGCACCCCACGCCCTTCTACGCCGCGGGCGAG
AAGCCGGCCGAGACGTACGGCGGCATCCCCGGCACGCACATCGGCGTGGAAGTGTCGATG
CAGTGGAACAGCGGCTACACCGAGCAGGTGCTCTGTTTCACCAACAACATCCCGCAGCGC
GACGGCGGCACCCACCTGACCGGCCTGCGCGCCGCGATGACCCGGATCATCAACAAGTAC
ATCGAAGAAAACGAGCTGGCCAAGAAGGCCAAGGTCGAGGTAACCGGCGACGACATGCGC
GAAGAAGAGGACATGGCCAAGCGCCGTTCGCGCCTGAAGGCGTTGATCACGCTCGGCAAG
ACCCGCGGCTACCTGACGCAGGTCGAGATCTCCGACCACCTGCCCGACAAGCTGGTCGAC
GCCGAGACGATGGAAGTCGTGATCGCCATGTTGAACGACATGGGCATCGCGGTCTACGAG
CAGACGCCCGACGCCGAGACGCTGTTCCAGAACAACGTCACCCCCACCGCCGCCACTGTC
GAGGAAGCCGAGGAAGAAGCCGAGGCCGCGCTCTCCACCGTCGACAGCGAATTCGGCCGC
ACCACCGACCCGGTCCGCATGTACATGCGCGAAATGGGCACCGTCGAGCTGCTGACCCGC
GAAGGCGAGATCGAGATCGCCAAGCGCATCGAAGGCGGCCTGCAGGCCATGATGGAGGCG
ATCAGCGCGTCGCCCGCCACCATCGCCGCGATCCTGGTGATGGGCGAGGACATCCGCGAA
GGCAAGGTCGTCATCTCGACCATCGTCGACGGCTTCTCCAACCCCAACGAAGCCGACGAC
TACGTGGCCGAGGAGGACTTCGACGAGTTCGACGAGGAAGACGACGACGACGGCAAGGGC
GGCTCCAAGGCGCTGACCAAGAAGCTGGAAGAATTGAAGCGCGAGGCGCTGTCGCGCTTC

GAC
>CFBP1798 Xylophilus ampelinus
CACGCCGGCGGCAAGTTCAACCAGAACAGCTACAAGGTCTCGGGCGGCCTGCACGGCGTG
GGCGTGAGTTGCGTCAACGCGCTCAGCAAGATGCTGCGCCTGACGGTGCGGCGCGACGGC
AAGGTCCACGTGCTGGAGTTCAGCAAGGGCTTCGTGCAGAACCGCATCGTCGAGCAGGCG
GGCGGCGTCGAGGTCTCGCCGATGCGCATCATGGGCGACACCGAGAAGCGCGGTACCGAG
GTGCACTTCCTGCCCGACAACGAGATCTTCAAGGAAAACGCCGATTTCCACTACGAGATC
CTGGCCAAGCGCCTGCGCGAACTGAGCTTCCTGAACAACGGCGTGCGCATCCGCCTGAAG
GACGAGCGCAGCGGCAAGGAAGACGATTTCTCCGGCGCCGGCGGCGTGCAGGGTTTCGTC
GAATTCATCAACAAGGGCAAGACCATCCTGCACCCCACGCCCTTCTACGCCGCGGGCGAG
AAGCCGGCCGAGACGTACGGCGGCATCCCCGGCACGCACATCGGCGTGGAAGTGTCGATG
CAGTGGAACAGCGGCTACACCGAGCAGGTGCTCTGTTTCACCAACAACATCCCGCAGCGC
GACGGCGGCACCCACCTGACCGGCCTGCGCGCCGCGATGACCCGGATCATCAACAAGTAC
ATCGAAGAAAACGAGCTGGCCAAGAAGGCCAAGGTCGAGGTAACCGGCGACGACATGCGC
GAAGAAGAGGACATGGCCAAGCGCCGTTCGCGCCTGAAGGCGTTGATCACGCTCGGCAAG
ACCCGCGGCTACCTGACGCAGGTCGAGATCTCCGACCACCTGCCCGACAAGCTGGTCGAC
GCCGAGACGATGGAAGTCGTGATCGCCATGTTGAACGACATGGGCATCGCGGTCTACGAG
CAGACGCCCGACGCCGAGACGCTGTTCCAGAACAACGTCACCCCCACCGCCGCCACTGTC
GAGGAAGCCGAGGAAGAAGCCGAGGCCGCGCTCTCCACCGTCGACAGCGAATTCGGCCGC
ACCACCGACCCGGTCCGCATGTACATGCGCGAAATGGGCACCGTCGAGCTGCTGACCCGC
GAAGGCGAGATCGAGATCGCCAAGCGCATCGAAGGCGGCCTGCAGGCCATGATGGAGGCG
ATCAGCGCGTCGCCCGCCACCATCGCCGCGATCCTGGTGATGGGCGAGGACATCCGCGAA
GGCAAGGTCGTCATCTCGACCATCGTCGACGGCTTCTCCAACCCCAACGAAGCCGACGAC
TACGTGGCCGAGGAGGACTTCGACGAGTTCGACGAGGAAGACGACGACGACGGCAAGGGC
GGCTCCAAGGCGCTGACCAAGAAGCTGGAAGAATTGAAGCGCGAGGCGCTGTCGCGCTTC
GAC
>CFBP1799 Xylophilus ampelinus
CACGCCGGCGGCAAGTTCAACCAGAACAGCTACAAGGTCTCGGGCGGCCTGCACGGCGTG
GGCGTGAGTTGCGTCAACGCGCTCAGCAAGATGCTGCGCCTGACGGTGCGGCGCGACGGC
AAGGTCCACGTGCTGGAGTTCAGCAAGGGCTTCGTGCAGAACCGCATCGTCGAGCAGGCG
GGCGGCGTCGAGGTCTCGCCGATGCGCATCATGGGCGACACCGAGAAGCGCGGTACCGAG
GTGCACTTCCTGCCCGACAACGAGATCTTCAAGGAAAACGCCGATTTCCACTACGAGATC
CTGGCCAAGCGCCTGCGCGAACTGAGCTTCCTGAACAACGGCGTGCGCATCCGCCTGAAG
GACGAGCGCAGCGGCAAGGAAGACGATTTCTCCGGCGCCGGCGGCGTGCAGGGTTTCGTC
GAATTCATCAACAAGGGCAAGACCATCCTGCACCCCACGCCCTTCTACGCCGCGGGCGAG
AAGCCGGCCGAGACGTACGGCGGCATCCCCGGCACGCACATCGGCGTGGAAGTGTCGATG
CAGTGGAACAGCGGCTACACCGAGCAGGTGCTCTGTTTCACCAACAACATCCCGCAGCGC
GACGGCGGCACCCACCTGACCGGCCTGCGCGCCGCGATGACCCGGATCATCAACAAGTAC
ATCGAAGAAAACGAGCTGGCCAAGAAGGCCAAGGTCGAGGTAACCGGCGACGACATGCGC
GAAGAAGAGGACATGGCCAAGCGCCGTTCGCGCCTGAAGGCGTTGATCACGCTCGGCAAG
ACCCGCGGCTACCTGACGCAGGTCGAGATCTCCGACCACCTGCCCGACAAGCTGGTCGAC
GCCGAGACGATGGAAGTCGTGATCGCCATGTTGAACGACATGGGCATCGCGGTCTACGAG
CAGACGCCCGACGCCGAGACGCTGTTCCAGAACAACGTCACCCCCACCGCCGCCACTGTC
GAGGAAGCCGAGGAAGAAGCCGAGGCCGCGCTCTCCACCGTCGACAGCGAATTCGGCCGC
ACCACCGACCCGGTCCGCATGTACATGCGCGAAATGGGCACCGTCGAGCTGCTGACCCGC
GAAGGCGAGATCGAGATCGCCAAGCGCATCGAAGGCGGCCTGCAGGCCATGATGGAGGCG
ATCAGCGCGTCGCCCGCCACCATCGCCGCGATCCTGGTGATGGGCGAGGACATCCGCGAA
GGCAAGGTCGTCATCTCGACCATCGTCGACGGCTTCTCCAACCCCAACGAAGCCGACGAC
TACGTGGCCGAGGAGGACTTCGACGAGTTCGACGAGGAAGACGACGACGACGGCAAGGGC
GGCTCCAAGGCGCTGACCAAGAAGCTGGAAGAATTGAAGCGCGAGGCGCTGTCGCGCTTC
GAC
>CFBP1800 Xylophilus ampelinus
CACGCCGGCGGCAAGTTCAACCAGAACAGCTACAAGGTCTCGGGCGGCCTGCACGGCGTG
GGCGTGAGTTGCGTCAACGCGCTCAGCAAGATGCTGCGCCTGACGGTGCGGCGCGACGGC
AAGGTCCACGTGCTGGAGTTCAGCAAGGGCTTCGTGCAGAACCGCATCGTCGAGCAGGCG
GGCGGCGTCGAGGTCTCGCCGATGCGCATCATGGGCGACACCGAGAAGCGCGGTACCGAG
GTGCACTTCCTGCCCGACAACGAGATCTTCAAGGAAAACGCCGATTTCCACTACGAGATC
CTGGCCAAGCGCCTGCGCGAACTGAGCTTCCTGAACAACGGCGTGCGCATCCGCCTGAAG

GACGAGCGCAGCGGCAAGGAAGACGATTTCTCCGGCGCCGGCGGCGTGCAGGGTTTCGTC
GAATTCATCAACAAGGGCAAGACCATCCTGCACCCCACGCCCTTCTACGCCGCGGGCGAG
AAGCCGGCCGAGACGTACGGCGGCATCCCCGGCACGCACATCGGCGTGGAAGTGTCGATG
CAGTGGAACAGCGGCTACACCGAGCAGGTGCTCTGTTTCACCAACAACATCCCGCAGCGC
GACGGCGGCACCCACCTGACCGGCCTGCGCGCCGCGATGACCCGGATCATCAACAAGTAC
ATCGAAGAAAACGAGCTGGCCAAGAAGGCCAAGGTCGAGGTAACCGGCGACGACATGCGC
GAAGAAGAGGACATGGCCAAGCGCCGTTCGCGCCTGAAGGCGTTGATCACGCTCGGCAAG
ACCCGCGGCTACCTGACGCAGGTCGAGATCTCCGACCACCTGCCCGACAAGCTGGTCGAC
GCCGAGACGATGGAAGTCGTGATCGCCATGTTGAACGACATGGGCATCGCGGTCTACGAG
CAGACGCCCGACGCCGAGACGCTGTTCCAGAACAACGTCACCCCCACCGCCGCCACTGTC
GAGGAAGCCGAGGAAGAAGCCGAGGCCGCGCTCTCCACCGTCGACAGCGAATTCGGCCGC
ACCACCGACCCGGTCCGCATGTACATGCGCGAAATGGGCACCGTCGAGCTGCTGACCCGC
GAAGGCGAGATCGAGATCGCCAAGCGCATCGAAGGCGGCCTGCAGGCCATGATGGAGGCG
ATCAGCGCGTCGCCCGCCACCATCGCCGCGATCCTGGTGATGGGCGAGGACATCCGCGAA
GGCAAGGTCGTCATCTCGACCATCGTCGACGGCTTCTCCAACCCCAACGAAGCCGACGAC
TACGTGGCCGAGGAGGACTTCGACGAGTTCGACGAGGAAGACGACGACGACGGCAAGGGC
GGCTCCAAGGCGCTGACCAAGAAGCTGGAAGAATTGAAGCGCGAGGCGCTGTCGCGCTTC
GAC
>CFBP1801 Xylophilus ampelinus
CACGCCGGCGGCAAGTTCAACCAGAACAGCTACAAGGTCTCGGGCGGCCTGCACGGCGTG
GGCGTGAGTTGCGTCAACGCGCTCAGCAAGATGCTGCGCCTGACGGTGCGGCGCGACGGC
AAGGTCCACGTGCTGGAGTTCAGCAAGGGCTTCGTGCAGAACCGCATCGTCGAGCAGGCG
GGCGGCGTCGAGGTCTCGCCGATGCGCATCATGGGCGACACCGAGAAGCGCGGTACCGAG
GTGCACTTCCTGCCCGACAACGAGATCTTCAAGGAAAACGCCGATTTCCACTACGAGATC
CTGGCCAAGCGCCTGCGCGAACTGAGCTTCCTGAACAACGGCGTGCGCATCCGCCTGAAG
GACGAGCGCAGCGGCAAGGAAGACGATTTCTCCGGCGCCGGCGGCGTGCAGGGTTTCGTC
GAATTCATCAACAAGGGCAAGACCATCCTGCACCCCACGCCCTTCTACGCCGCGGGCGAG
AAGCCGGCCGAGACGTACGGCGGCATCCCCGGCACGCACATCGGCGTGGAAGTGTCGATG
CAGTGGAACAGCGGCTACACCGAGCAGGTGCTCTGTTTCACCAACAACATCCCGCAGCGC
GACGGCGGCACCCACCTGACCGGCCTGCGCGCCGCGATGACCCGGATCATCAACAAGTAC
ATCGAAGAAAACGAGCTGGCCAAGAAGGCCAAGGTCGAGGTAACCGGCGACGACATGCGC
GAAGAAGAGGACATGGCCAAGCGCCGTTCGCGCCTGAAGGCGTTGATCACGCTCGGCAAG
ACCCGCGGCTACCTGACGCAGGTCGAGATCTCCGACCACCTGCCCGACAAGCTGGTCGAC
GCCGAGACGATGGAAGTCGTGATCGCCATGTTGAACGACATGGGCATCGCGGTCTACGAG
CAGACGCCCGACGCCGAGACGCTGTTCCAGAACAACGTCACCCCCACCGCCGCCACTGTC
GAGGAAGCCGAGGAAGAAGCCGAGGCCGCGCTCTCCACCGTCGACAGCGAATTCGGCCGC
ACCACCGACCCGGTCCGCATGTACATGCGCGAAATGGGCACCGTCGAGCTGCTGACCCGC
GAAGGCGAGATCGAGATCGCCAAGCGCATCGAAGGCGGCCTGCAGGCCATGATGGAGGCG
ATCAGCGCGTCGCCCGCCACCATCGCCGCGATCCTGGTGATGGGCGAGGACATCCGCGAA
GGCAAGGTCGTCATCTCGACCATCGTCGACGGCTTCTCCAACCCCAACGAAGCCGACGAC
TACGTGGCCGAGGAGGACTTCGACGAGTTCGACGAGGAAGACGACGACGACGGCAAGGGC
GGCTCCAAGGCGCTGACCAAGAAGCTGGAAGAATTGAAGCGCGAGGCGCTGTCGCGCTTC
GAC
>CFBP1802 Xylophilus ampelinus
CACGCCGGCGGCAAGTTCAACCAGAACAGCTACAAGGTCTCGGGCGGCCTGCACGGCGTG
GGCGTGAGTTGCGTCAACGCGCTCAGCAAGATGCTGCGCCTGACGGTGCGGCGCGACGGC
AAGGTCCACGTGCTGGAGTTCAGCAAGGGCTTCGTGCAGAACCGCATCGTCGAGCAGGCG
GGCGGCGTCGAGGTCTCGCCGATGCGCATCATGGGCGACACCGAGAAGCGCGGTACCGAG
GTGCACTTCCTGCCCGACAACGAGATCTTCAAGGAAAACGCCGATTTCCACTACGAGATC
CTGGCCAAGCGCCTGCGCGAACTGAGCTTCCTGAACAACGGCGTGCGCATCCGCCTGAAG
GACGAGCGCAGCGGCAAGGAAGACGATTTCTCCGGCGCCGGCGGCGTGCAGGGTTTCGTC
GAATTCATCAACAAGGGCAAGACCATCCTGCACCCCACGCCCTTCTACGCCGCGGGCGAG
AAGCCGGCCGAGACGTACGGCGGCATCCCCGGCACGCACATCGGCGTGGAAGTGTCGATG
CAGTGGAACAGCGGCTACACCGAGCAGGTGCTCTGTTTCACCAACAACATCCCGCAGCGC
GACGGCGGCACCCACCTGACCGGCCTGCGCGCCGCGATGACCCGGATCATCAACAAGTAC
ATCGAAGAAAACGAGCTGGCCAAGAAGGCCAAGGTCGAGGTAACCGGCGACGACATGCGC
GAAGAAGAGGACATGGCCAAGCGCCGTTCGCGCCTGAAGGCGTTGATCACGCTCGGCAAG
ACCCGCGGCTACCTGACGCAGGTCGAGATCTCCGACCACCTGCCCGACAAGCTGGTCGAC

GCCGAGACGATGGAAGTCGTGATCGCCATGTTGAACGACATGGGCATCGCGGTCTACGAG
CAGACGCCCGACGCCGAGACGCTGTTCCAGAACAACGTCACCCCCACCGCCGCCACTGTC
GAGGAAGCCGAGGAAGAAGCCGAGGCCGCGCTCTCCACCGTCGACAGCGAATTCGGCCGC
ACCACCGACCCGGTCCGCATGTACATGCGCGAAATGGGCACCGTCGAGCTGCTGACCCGC
GAAGGCGAGATCGAGATCGCCAAGCGCATCGAAGGCGGCCTGCAGGCCATGATGGAGGCG
ATCAGCGCGTCGCCCGCCACCATCGCCGCGATCCTGGTGATGGGCGAGGACATCCGCGAA
GGCAAGGTCGTCATCTCGACCATCGTCGACGGCTTCTCCAACCCCAACGAAGCCGACGAC
TACGTGGCCGAGGAGGACTTCGACGAGTTCGACGAGGAAGACGACGACGACGGCAAGGGC
GGCTCCAAGGCGCTGACCAAGAAGCTGGAAGAATTGAAGCGCGAGGCGCTGTCGCGCTTC
GAC
>CFBP1803 Xylophilus ampelinus
CACGCCGGCGGCAAGTTCAACCAGAACAGCTACAAGGTCTCGGGCGGCCTGCACGGCGTG
GGCGTGAGTTGCGTCAACGCGCTCAGCAAGATGCTGCGCCTGACGGTGCGGCGCGACGGC
AAGGTCCACGTGCTGGAGTTCAGCAAGGGCTTCGTGCAGAACCGCATCGTCGAGCAGGCG
GGCGGCGTCGAGGTCTCGCCGATGCGCATCATGGGCGACACCGAGAAGCGCGGTACCGAG
GTGCACTTCCTGCCCGACAACGAGATCTTCAAGGAAAACGCCGATTTCCACTACGAGATC
CTGGCCAAGCGCCTGCGCGAACTGAGCTTCCTGAACAACGGCGTGCGCATCCGCCTGAAG
GACGAGCGCAGCGGCAAGGAAGACGATTTCTCCGGCGCCGGCGGCGTGCAGGGTTTCGTC
GAATTCATCAACAAGGGCAAGACCATCCTGCACCCCACGCCCTTCTACGCCGCGGGCGAG
AAGCCGGCCGAGACGTACGGCGGCATCCCCGGCACGCACATCGGCGTGGAAGTGTCGATG
CAGTGGAACAGCGGCTACACCGAGCAGGTGCTCTGTTTCACCAACAACATCCCGCAGCGC
GACGGCGGCACCCACCTGACCGGCCTGCGCGCCGCGATGACCCGGATCATCAACAAGTAC
ATCGAAGAAAACGAGCTGGCCAAGAAGGCCAAGGTCGAGGTAACCGGCGACGACATGCGC
GAAGAAGAGGACATGGCCAAGCGCCGTTCGCGCCTGAAGGCGTTGATCACGCTCGGCAAG
ACCCGCGGCTACCTGACGCAGGTCGAGATCTCCGACCACCTGCCCGACAAGCTGGTCGAC
GCCGAGACGATGGAAGTCGTGATCGCCATGTTGAACGACATGGGCATCGCGGTCTACGAG
CAGACGCCCGACGCCGAGACGCTGTTCCAGAACAACGTCACCCCCACCGCCGCCACTGTC
GAGGAAGCCGAGGAAGAAGCCGAGGCCGCGCTCTCCACCGTCGACAGCGAATTCGGCCGC
ACCACCGACCCGGTCCGCATGTACATGCGCGAAATGGGCACCGTCGAGCTGCTGACCCGC
GAAGGCGAGATCGAGATCGCCAAGCGCATCGAAGGCGGCCTGCAGGCCATGATGGAGGCG
ATCAGCGCGTCGCCCGCCACCATCGCCGCGATCCTGGTGATGGGCGAGGACATCCGCGAA
GGCAAGGTCGTCATCTCGACCATCGTCGACGGCTTCTCCAACCCCAACGAAGCCGACGAC
TACGTGGCCGAGGAGGACTTCGACGAGTTCGACGAGGAAGACGACGACGACGGCAAGGGC
GGCTCCAAGGCGCTGACCAAGAAGCTGGAAGAATTGAAGCGCGAGGCGCTGTCGCGCTTC
GAC
>CFBP1833 Xylophilus ampelinus
CACGCCGGCGGCAAGTTCAACCAGAACAGCTACAAGGTCTCGGGCGGCCTGCACGGCGTG
GGCGTGAGTTGCGTCAACGCGCTCAGCAAGATGCTGCGCCTGACGGTGCGGCGCGACGGC
AAGGTCCACGTGCTGGAGTTCAGCAAGGGCTTCGTGCAGAACCGCATCGTCGAGCAGGCG
GGCGGCGTCGAGGTCTCGCCGATGCGCATCATGGGCGACACCGAGAAGCGCGGTACCGAG
GTGCACTTCCTGCCCGACAACGAGATCTTCAAGGAAAACGCCGATTTCCACTACGAGATC
CTGGCCAAGCGCCTGCGCGAACTGAGCTTCCTGAACAACGGCGTGCGCATCCGCCTGAAG
GACGAGCGCAGCGGCAAGGAAGACGATTTCTCCGGCGCCGGCGGCGTGCAGGGTTTCGTC
GAATTCATCAACAAGGGCAAGACCATCCTGCACCCCACGCCCTTCTACGCCGCGGGCGAG
AAGCCGGCCGAGACGTACGGCGGCATCCCCGGCACGCACATCGGCGTGGAAGTGTCGATG
CAGTGGAACAGCGGCTACACCGAGCAGGTGCTCTGTTTCACCAACAACATCCCGCAGCGC
GACGGCGGCACCCACCTGACCGGCCTGCGCGCCGCGATGACCCGGATCATCAACAAGTAC
ATCGAAGAAAACGAGCTGGCCAAGAAGGCCAAGGTCGAGGTAACCGGCGACGACATGCGC
GAAGAAGAGGACATGGCCAAGCGCCGTTCGCGCCTGAAGGCGTTGATCACGCTCGGCAAG
ACCCGCGGCTACCTGACGCAGGTCGAGATCTCCGACCACCTGCCCGACAAGCTGGTCGAC
GCCGAGACGATGGAAGTCGTGATCGCCATGTTGAACGACATGGGCATCGCGGTCTACGAG
CAGACGCCCGACGCCGAGACGCTGTTCCAGAACAACGTCACCCCCACCGCCGCCACTGTC
GAGGAAGCCGAGGAAGAAGCCGAGGCCGCGCTCTCCACCGTCGACAGCGAATTCGGCCGC
ACCACCGACCCGGTCCGCATGTACATGCGCGAAATGGGCACCGTCGAGCTGCTGACCCGC
GAAGGCGAGATCGAGATCGCCAAGCGCATCGAAGGCGGCCTGCAGGCCATGATGGAGGCG
ATCAGCGCGTCGCCCGCCACCATCGCCGCGATCCTGGTGATGGGCGAGGACATCCGCGAA
GGCAAGGTCGTCATCTCGACCATCGTCGACGGCTTCTCCAACCCCAACGAAGCCGACGAC
TACGTGGCCGAGGAGGACTTCGACGAGTTCGACGAGGAAGACGACGACGACGGCAAGGGC

GGCTCCAAGGCGCTGACCAAGAAGCTGGAAGAATTGAAGCGCGAGGCGCTGTCGCGCTTC
GAC
>CFBP1834 Xylophilus ampelinus
CACGCCGGCGGCAAGTTCAACCAGAACAGCTACAAGGTCTCGGGCGGCCTGCACGGCGTG
GGCGTGAGTTGCGTCAACGCGCTCAGCAAGATGCTGCGCCTGACGGTGCGGCGCGACGGC
AAGGTCCACGTGCTGGAGTTCAGCAAGGGCTTCGTGCAGAACCGCATCGTCGAGCAGGCG
GGCGGCGTCGAGGTCTCGCCGATGCGCATCATGGGCGACACCGAGAAGCGCGGTACCGAG
GTGCACTTCCTGCCCGACAACGAGATCTTCAAGGAAAACGCCGATTTCCACTACGAGATC
CTGGCCAAGCGCCTGCGCGAACTGAGCTTCCTGAACAACGGCGTGCGCATCCGCCTGAAG
GACGAGCGCAGCGGCAAGGAAGACGATTTCTCCGGCGCCGGCGGCGTGCAGGGTTTCGTC
GAATTCATCAACAAGGGCAAGACCATCCTGCACCCCACGCCCTTCTACGCCGCGGGCGAG
AAGCCGGCCGAGACGTACGGCGGCATCCCCGGCACGCACATCGGCGTGGAAGTGTCGATG
CAGTGGAACAGCGGCTACACCGAGCAGGTGCTCTGTTTCACCAACAACATCCCGCAGCGC
GACGGCGGCACCCACCTGACCGGCCTGCGCGCCGCGATGACCCGGATCATCAACAAGTAC
ATCGAAGAAAACGAGCTGGCCAAGAAGGCCAAGGTCGAGGTAACCGGCGACGACATGCGC
GAAGAAGAGGACATGGCCAAGCGCCGTTCGCGCCTGAAGGCGTTGATCACGCTCGGCAAG
ACCCGCGGCTACCTGACGCAGGTCGAGATCTCCGACCACCTGCCCGACAAGCTGGTCGAC
GCCGAGACGATGGAAGTCGTGATCGCCATGTTGAACGACATGGGCATCGCGGTCTACGAG
CAGACGCCCGACGCCGAGACGCTGTTCCAGAACAACGTCACCCCCACCGCCGCCACTGTC
GAGGAAGCCGAGGAAGAAGCCGAGGCCGCGCTCTCCACCGTCGACAGCGAATTCGGCCGC
ACCACCGACCCGGTCCGCATGTACATGCGCGAAATGGGCACCGTCGAGCTGCTGACCCGC
GAAGGCGAGATCGAGATCGCCAAGCGCATCGAAGGCGGCCTGCAGGCCATGATGGAGGCG
ATCAGCGCGTCGCCCGCCACCATCGCCGCGATCCTGGTGATGGGCGAGGACATCCGCGAA
GGCAAGGTCGTCATCTCGACCATCGTCGACGGCTTCTCCAACCCCAACGAAGCCGACGAC
TACGTGGCCGAGGAGGACTTCGACGAGTTCGACGAGGAAGACGACGACGACGGCAAGGGC
GGCTCCAAGGCGCTGACCAAGAAGCTGGAAGAATTGAAGCGCGAGGCGCTGTCGCGCTTC
GAC
>CFBP1835 Xylophilus ampelinus
CACGCCGGCGGCAAGTTCAACCAGAACAGCTACAAGGTCTCGGGCGGCCTGCACGGCGTG
GGCGTGAGTTGCGTCAACGCGCTCAGCAAGATGCTGCGCCTGACGGTGCGGCGCGACGGC
AAGGTCCACGTGCTGGAGTTCAGCAAGGGCTTCGTGCAGAACCGCATCGTCGAGCAGGCG
GGCGGCGTCGAGGTCTCGCCGATGCGCATCATGGGCGACACCGAGAAGCGCGGTACCGAG
GTGCACTTCCTGCCCGACAACGAGATCTTCAAGGAAAACGCCGATTTCCACTACGAGATC
CTGGCCAAGCGCCTGCGCGAACTGAGCTTCCTGAACAACGGCGTGCGCATCCGCCTGAAG
GACGAGCGCAGCGGCAAGGAAGACGATTTCTCCGGCGCCGGCGGCGTGCAGGGTTTCGTC
GAATTCATCAACAAGGGCAAGACCATCCTGCACCCCACGCCCTTCTACGCCGCGGGCGAG
AAGCCGGCCGAGACGTACGGCGGCATCCCCGGCACGCACATCGGCGTGGAAGTGTCGATG
CAGTGGAACAGCGGCTACACCGAGCAGGTGCTCTGTTTCACCAACAACATCCCGCAGCGC
GACGGCGGCACCCACCTGACCGGCCTGCGCGCCGCGATGACCCGGATCATCAACAAGTAC
ATCGAAGAAAACGAGCTGGCCAAGAAGGCCAAGGTCGAGGTAACCGGCGACGACATGCGC
GAAGAAGAGGACATGGCCAAGCGCCGTTCGCGCCTGAAGGCGTTGATCACGCTCGGCAAG
ACCCGCGGCTACCTGACGCAGGTCGAGATCTCCGACCACCTGCCCGACAAGCTGGTCGAC
GCCGAGACGATGGAAGTCGTGATCGCCATGTTGAACGACATGGGCATCGCGGTCTACGAG
CAGACGCCCGACGCCGAGACGCTGTTCCAGAACAACGTCACCCCCACCGCCGCCACTGTC
GAGGAAGCCGAGGAAGAAGCCGAGGCCGCGCTCTCCACCGTCGACAGCGAATTCGGCCGC
ACCACCGACCCGGTCCGCATGTACATGCGCGAAATGGGCACCGTCGAGCTGCTGACCCGC
GAAGGCGAGATCGAGATCGCCAAGCGCATCGAAGGCGGCCTGCAGGCCATGATGGAGGCG
ATCAGCGCGTCGCCCGCCACCATCGCCGCGATCCTGGTGATGGGCGAGGACATCCGCGAA
GGCAAGGTCGTCATCTCGACCATCGTCGACGGCTTCTCCAACCCCAACGAAGCCGACGAC
TACGTGGCCGAGGAGGACTTCGACGAGTTCGACGAGGAAGACGACGACGACGGCAAGGGC
GGCTCCAAGGCGCTGACCAAGAAGCTGGAAGAATTGAAGCGCGAGGCGCTGTCGCGCTTC
GAC
>CFBP1836 Xylophilus ampelinus
CACGCCGGCGGCAAGTTCAACCAGAACAGCTACAAGGTCTCGGGCGGCCTGCACGGCGTG
GGCGTGAGTTGCGTCAACGCGCTCAGCAAGATGCTGCGCCTGACGGTGCGGCGCGACGGC
AAGGTCCACGTGCTGGAGTTCAGCAAGGGCTTCGTGCAGAACCGCATCGTCGAGCAGGCG
GGCGGCGTCGAGGTCTCGCCGATGCGCATCATGGGCGACACCGAGAAGCGCGGTACCGAG
GTGCACTTCCTGCCCGACAACGAGATCTTCAAGGAAAACGCCGATTTCCACTACGAGATC

CTGGCCAAGCGCCTGCGCGAACTGAGCTTCCTGAACAACGGCGTGCGCATCCGCCTGAAG
GACGAGCGCAGCGGCAAGGAAGACGATTTCTCCGGCGCCGGCGGCGTGCAGGGTTTCGTC
GAATTCATCAACAAGGGCAAGACCATCCTGCACCCCACGCCCTTCTACGCCGCGGGCGAG
AAGCCGGCCGAGACGTACGGCGGCATCCCCGGCACGCACATCGGCGTGGAAGTGTCGATG
CAGTGGAACAGCGGCTACACCGAGCAGGTGCTCTGTTTCACCAACAACATCCCGCAGCGC
GACGGCGGCACCCACCTGACCGGCCTGCGCGCCGCGATGACCCGGATCATCAACAAGTAC
ATCGAAGAAAACGAGCTGGCCAAGAAGGCCAAGGTCGAGGTAACCGGCGACGACATGCGC
GAAGAAGAGGACATGGCCAAGCGCCGTTCGCGCCTGAAGGCGTTGATCACGCTCGGCAAG
ACCCGCGGCTACCTGACGCAGGTCGAGATCTCCGACCACCTGCCCGACAAGCTGGTCGAC
GCCGAGACGATGGAAGTCGTGATCGCCATGTTGAACGACATGGGCATCGCGGTCTACGAG
CAGACGCCCGACGCCGAGACGCTGTTCCAGAACAACGTCACCCCCACCGCCGCCACTGTC
GAGGAAGCCGAGGAAGAAGCCGAGGCCGCGCTCTCCACCGTCGACAGCGAATTCGGCCGC
ACCACCGACCCGGTCCGCATGTACATGCGCGAAATGGGCACCGTCGAGCTGCTGACCCGC
GAAGGCGAGATCGAGATCGCCAAGCGCATCGAAGGCGGCCTGCAGGCCATGATGGAGGCG
ATCAGCGCGTCGCCCGCCACCATCGCCGCGATCCTGGTGATGGGCGAGGACATCCGCGAA
GGCAAGGTCGTCATCTCGACCATCGTCGACGGCTTCTCCAACCCCAACGAAGCCGACGAC
TACGTGGCCGAGGAGGACTTCGACGAGTTCGACGAGGAAGACGACGACGACGGCAAGGGC
GGCTCCAAGGCGCTGACCAAGAAGCTGGAAGAATTGAAGCGCGAGGCGCTGTCGCGCTTC
GAC
>CFBP1837 Xylophilus ampelinus
CACGCCGGCGGCAAGTTCAACCAGAACAGCTACAAGGTCTCGGGCGGCCTGCACGGCGTG
GGCGTGAGTTGCGTCAACGCGCTCAGCAAGATGCTGCGCCTGACGGTGCGGCGCGACGGC
AAGGTCCACGTGCTGGAGTTCAGCAAGGGCTTCGTGCAGAACCGCATCGTCGAGCAGGCG
GGCGGCGTCGAGGTCTCGCCGATGCGCATCATGGGCGACACCGAGAAGCGCGGTACCGAG
GTGCACTTCCTGCCCGACAACGAGATCTTCAAGGAAAACGCCGATTTCCACTACGAGATC
CTGGCCAAGCGCCTGCGCGAACTGAGCTTCCTGAACAACGGCGTGCGCATCCGCCTGAAG
GACGAGCGCAGCGGCAAGGAAGACGATTTCTCCGGCGCCGGCGGCGTGCAGGGTTTCGTC
GAATTCATCAACAAGGGCAAGACCATCCTGCACCCCACGCCCTTCTACGCCGCGGGCGAG
AAGCCGGCCGAGACGTACGGCGGCATCCCCGGCACGCACATCGGCGTGGAAGTGTCGATG
CAGTGGAACAGCGGCTACACCGAGCAGGTGCTCTGTTTCACCAACAACATCCCGCAGCGC
GACGGCGGCACCCACCTGACCGGCCTGCGCGCCGCGATGACCCGGATCATCAACAAGTAC
ATCGAAGAAAACGAGCTGGCCAAGAAGGCCAAGGTCGAGGTAACCGGCGACGACATGCGC
GAAGAAGAGGACATGGCCAAGCGCCGTTCGCGCCTGAAGGCGTTGATCACGCTCGGCAAG
ACCCGCGGCTACCTGACGCAGGTCGAGATCTCCGACCACCTGCCCGACAAGCTGGTCGAC
GCCGAGACGATGGAAGTCGTGATCGCCATGTTGAACGACATGGGCATCGCGGTCTACGAG
CAGACGCCCGACGCCGAGACGCTGTTCCAGAACAACGTCACCCCCACCGCCGCCACTGTC
GAGGAAGCCGAGGAAGAAGCCGAGGCCGCGCTCTCCACCGTCGACAGCGAATTCGGCCGC
ACCACCGACCCGGTCCGCATGTACATGCGCGAAATGGGCACCGTCGAGCTGCTGACCCGC
GAAGGCGAGATCGAGATCGCCAAGCGCATCGAAGGCGGCCTGCAGGCCATGATGGAGGCG
ATCAGCGCGTCGCCCGCCACCATCGCCGCGATCCTGGTGATGGGCGAGGACATCCGCGAA
GGCAAGGTCGTCATCTCGACCATCGTCGACGGCTTCTCCAACCCCAACGAAGCCGACGAC
TACGTGGCCGAGGAGGACTTCGACGAGTTCGACGAGGAAGACGACGACGACGGCAAGGGC
GGCTCCAAGGCGCTGACCAAGAAGCTGGAAGAATTGAAGCGCGAGGCGCTGTCGCGCTTC
GAC
>CFBP1841 Xylophilus ampelinus
CACGCCGGCGGCAAGTTCAACCAGAACAGCTACAAGGTCTCGGGCGGCCTGCACGGCGTG
GGCGTGAGTTGCGTCAACGCGCTCAGCAAGATGCTGCGCCTGACGGTGCGGCGCGACGGC
AAGGTCCACGTGCTGGAGTTCAGCAAGGGCTTCGTGCAGAACCGCATCGTCGAGCAGGCG
GGCGGCGTCGAGGTCTCGCCGATGCGCATCATGGGCGACACCGAGAAGCGCGGTACCGAG
GTGCACTTCCTGCCCGACAACGAGATCTTCAAGGAAAACGCCGATTTCCACTACGAGATC
CTGGCCAAGCGCCTGCGCGAACTGAGCTTCCTGAACAACGGCGTGCGCATCCGCCTGAAG
GACGAGCGCAGCGGCAAGGAAGACGATTTCTCCGGCGCCGGCGGCGTGCAGGGTTTCGTC
GAATTCATCAACAAGGGCAAGACCATCCTGCACCCCACGCCCTTCTACGCCGCGGGCGAG
AAGCCGGCCGAGACGTACGGCGGCATCCCCGGCACGCACATCGGCGTGGAAGTGTCGATG
CAGTGGAACAGCGGCTACACCGAGCAGGTGCTCTGTTTCACCAACAACATCCCGCAGCGC
GACGGCGGCACCCACCTGACCGGCCTGCGCGCCGCGATGACCCGGATCATCAACAAGTAC
ATCGAAGAAAACGAGCTGGCCAAGAAGGCCAAGGTCGAGGTAACCGGCGACGACATGCGC
GAAGAAGAGGACATGGCCAAGCGCCGTTCGCGCCTGAAGGCGTTGATCACGCTCGGCAAG

ACCCGCGGCTACCTGACGCAGGTCGAGATCTCCGACCACCTGCCCGACAAGCTGGTCGAC
GCCGAGACGATGGAAGTCGTGATCGCCATGTTGAACGACATGGGCATCGCGGTCTACGAG
CAGACGCCCGACGCCGAGACGCTGTTCCAGAACAACGTCACCCCCACCGCCGCCACTGTC
GAGGAAGCCGAGGAAGAAGCCGAGGCCGCGCTCTCCACCGTCGACAGCGAATTCGGCCGC
ACCACCGACCCGGTCCGCATGTACATGCGCGAAATGGGCACCGTCGAGCTGCTGACCCGC
GAAGGCGAGATCGAGATCGCCAAGCGCATCGAAGGCGGCCTGCAGGCCATGATGGAGGCG
ATCAGCGCGTCGCCCGCCACCATCGCCGCGATCCTGGTGATGGGCGAGGACATCCGCGAA
GGCAAGGTCGTCATCTCGACCATCGTCGACGGCTTCTCCAACCCCAACGAAGCCGACGAC
TACGTGGCCGAGGAGGACTTCGACGAGTTCGACGAGGAAGACGACGACGACGGCAAGGGC
GGCTCCAAGGCGCTGACCAAGAAGCTGGAAGAATTGAAGCGCGAGGCGCTGTCGCGCTTC
GAC
>CFBP1842 Xylophilus ampelinus
CACGCCGGCGGCAAGTTCAACCAGAACAGCTACAAGGTCTCGGGCGGCCTGCACGGCGTG
GGCGTGAGTTGCGTCAACGCGCTCAGCAAGATGCTGCGCCTGACGGTGCGGCGCGACGGC
AAGGTCCACGTGCTGGAGTTCAGCAAGGGCTTCGTGCAGAACCGCATCGTCGAGCAGGCG
GGCGGCGTCGAGGTCTCGCCGATGCGCATCATGGGCGACACCGAGAAGCGCGGTACCGAG
GTGCACTTCCTGCCCGACAACGAGATCTTCAAGGAAAACGCCGATTTCCACTACGAGATC
CTGGCCAAGCGCCTGCGCGAACTGAGCTTCCTGAACAACGGCGTGCGCATCCGCCTGAAG
GACGAGCGCAGCGGCAAGGAAGACGATTTCTCCGGCGCCGGCGGCGTGCAGGGTTTCGTC
GAATTCATCAACAAGGGCAAGACCATCCTGCACCCCACGCCCTTCTACGCCGCGGGCGAG
AAGCCGGCCGAGACGTACGGCGGCATCCCCGGCACGCACATCGGCGTGGAAGTGTCGATG
CAGTGGAACAGCGGCTACACCGAGCAGGTGCTCTGTTTCACCAACAACATCCCGCAGCGC
GACGGCGGCACCCACCTGACCGGCCTGCGCGCCGCGATGACCCGGATCATCAACAAGTAC
ATCGAAGAAAACGAGCTGGCCAAGAAGGCCAAGGTCGAGGTAACCGGCGACGACATGCGC
GAAGAAGAGGACATGGCCAAGCGCCGTTCGCGCCTGAAGGCGTTGATCACGCTCGGCAAG
ACCCGCGGCTACCTGACGCAGGTCGAGATCTCCGACCACCTGCCCGACAAGCTGGTCGAC
GCCGAGACGATGGAAGTCGTGATCGCCATGTTGAACGACATGGGCATCGCGGTCTACGAG
CAGACGCCCGACGCCGAGACGCTGTTCCAGAACAACGTCACCCCCACCGCCGCCACTGTC
GAGGAAGCCGAGGAAGAAGCCGAGGCCGCGCTCTCCACCGTCGACAGCGAATTCGGCCGC
ACCACCGACCCGGTCCGCATGTACATGCGCGAAATGGGCACCGTCGAGCTGCTGACCCGC
GAAGGCGAGATCGAGATCGCCAAGCGCATCGAAGGCGGCCTGCAGGCCATGATGGAGGCG
ATCAGCGCGTCGCCCGCCACCATCGCCGCGATCCTGGTGATGGGCGAGGACATCCGCGAA
GGCAAGGTCGTCATCTCGACCATCGTCGACGGCTTCTCCAACCCCAACGAAGCCGACGAC
TACGTGGCCGAGGAGGACTTCGACGAGTTCGACGAGGAAGACGACGACGACGGCAAGGGC
GGCTCCAAGGCGCTGACCAAGAAGCTGGAAGAATTGAAGCGCGAGGCGCTGTCGCGCTTC
GAC
>CFBP1927 Xylophilus ampelinus
CACGCCGGCGGCAAGTTCAACCAGAACAGCTACAAGGTCTCGGGCGGCCTGCACGGCGTG
GGCGTGAGTTGCGTCAACGCGCTCAGCAAGATGCTGCGCCTGACGGTGCGGCGCGACGGC
AAGGTCCACGTGCTGGAGTTCAGCAAGGGCTTCGTGCAGAACCGCATCGTCGAGCAGGCG
GGCGGCGTCGAGGTCTCGCCGATGCGCATCATGGGCGACACCGAGAAGCGCGGTACCGAG
GTGCACTTCCTGCCCGACAACGAGATCTTCAAGGAAAACGCCGATTTCCACTACGAGATC
CTGGCCAAGCGCCTGCGCGAACTGAGCTTCCTGAACAACGGCGTGCGCATCCGCCTGAAG
GACGAGCGCAGCGGCAAGGAAGACGATTTCTCCGGCGCCGGCGGCGTGCAGGGTTTCGTC
GAATTCATCAACAAGGGCAAGACCATCCTGCACCCCACGCCCTTCTACGCCGCGGGCGAG
AAGCCGGCCGAGACGTACGGCGGCATCCCCGGCACGCACATCGGCGTGGAAGTGTCGATG
CAGTGGAACAGCGGCTACACCGAGCAGGTGCTCTGTTTCACCAACAACATCCCGCAGCGC
GACGGCGGCACCCACCTGACCGGCCTGCGCGCCGCGATGACCCGGATCATCAACAAGTAC
ATCGAAGAAAACGAGCTGGCCAAGAAGGCCAAGGTCGAGGTAACCGGCGACGACATGCGC
GAAGAAGAGGACATGGCCAAGCGCCGTTCGCGCCTGAAGGCGTTGATCACGCTCGGCAAG
ACCCGCGGCTACCTGACGCAGGTCGAGATCTCCGACCACCTGCCCGACAAGCTGGTCGAC
GCCGAGACGATGGAAGTCGTGATCGCCATGTTGAACGACATGGGCATCGCGGTCTACGAG
CAGACGCCCGACGCCGAGACGCTGTTCCAGAACAACGTCACCCCCACCGCCGCCACTGTC
GAGGAAGCCGAGGAAGAAGCCGAGGCCGCGCTCTCCACCGTCGACAGCGAATTCGGCCGC
ACCACCGACCCGGTCCGCATGTACATGCGCGAAATGGGCACCGTCGAGCTGCTGACCCGC
GAAGGCGAGATCGAGATCGCCAAGCGCATCGAAGGCGGCCTGCAGGCCATGATGGAGGCG
ATCAGCGCGTCGCCCGCCACCATCGCCGCGATCCTGGTGATGGGCGAGGACATCCGCGAA
GGCAAGGTCGTCATCTCGACCATCGTCGACGGCTTCTCCAACCCCAACGAAGCCGACGAC

TACGTGGCCGAGGAGGACTTCGACGAGTTCGACGAGGAAGACGACGACGACGGCAAGGGC
GGCTCCAAGGCGCTGACCAAGAAGCTGGAAGAATTGAAGCGCGAGGCGCTGTCGCGCTTC
GAC
>CFBP1928 Xylophilus ampelinus
CACGCCGGCGGCAAGTTCAACCAGAACAGCTACAAGGTCTCGGGCGGCCTGCACGGCGTG
GGCGTGAGTTGCGTCAACGCGCTCAGCAAGATGCTGCGCCTGACGGTGCGGCGCGACGGC
AAGGTCCACGTGCTGGAGTTCAGCAAGGGCTTCGTGCAGAACCGCATCGTCGAGCAGGCG
GGCGGCGTCGAGGTCTCGCCGATGCGCATCATGGGCGACACCGAGAAGCGCGGTACCGAG
GTGCACTTCCTGCCCGACAACGAGATCTTCAAGGAAAACGCCGATTTCCACTACGAGATC
CTGGCCAAGCGCCTGCGCGAACTGAGCTTCCTGAACAACGGCGTGCGCATCCGCCTGAAG
GACGAGCGCAGCGGCAAGGAAGACGATTTCTCCGGCGCCGGCGGCGTGCAGGGTTTCGTC
GAATTCATCAACAAGGGCAAGACCATCCTGCACCCCACGCCCTTCTACGCCGCGGGCGAG
AAGCCGGCCGAGACGTACGGCGGCATCCCCGGCACGCACATCGGCGTGGAAGTGTCGATG
CAGTGGAACAGCGGCTACACCGAGCAGGTGCTCTGTTTCACCAACAACATCCCGCAGCGC
GACGGCGGCACCCACCTGACCGGCCTGCGCGCCGCGATGACCCGGATCATCAACAAGTAC
ATCGAAGAAAACGAGCTGGCCAAGAAGGCCAAGGTCGAGGTAACCGGCGACGACATGCGC
GAAGAAGAGGACATGGCCAAGCGCCGTTCGCGCCTGAAGGCGTTGATCACGCTCGGCAAG
ACCCGCGGCTACCTGACGCAGGTCGAGATCTCCGACCACCTGCCCGACAAGCTGGTCGAC
GCCGAGACGATGGAAGTCGTGATCGCCATGTTGAACGACATGGGCATCGCGGTCTACGAG
CAGACGCCCGACGCCGAGACGCTGTTCCAGAACAACGTCACCCCCACCGCCGCCACTGTC
GAGGAAGCCGAGGAAGAAGCCGAGGCCGCGCTCTCCACCGTCGACAGCGAATTCGGCCGC
ACCACCGACCCGGTCCGCATGTACATGCGCGAAATGGGCACCGTCGAGCTGCTGACCCGC
GAAGGCGAGATCGAGATCGCCAAGCGCATCGAAGGCGGCCTGCAGGCCATGATGGAGGCG
ATCAGCGCGTCGCCCGCCACCATCGCCGCGATCCTGGTGATGGGCGAGGACATCCGCGAA
GGCAAGGTCGTCATCTCGACCATCGTCGACGGCTTCTCCAACCCCAACGAAGCCGACGAC
TACGTGGCCGAGGAGGACTTCGACGAGTTCGACGAGGAAGACGACGACGACGGCAAGGGC
GGCTCCAAGGCGCTGACCAAGAAGCTGGAAGAATTGAAGCGCGAGGCGCTGTCGCGCTTC
GAC
>CFBP1938 Xylophilus ampelinus
CACGCCGGCGGCAAGTTCAACCAGAACAGCTACAAGGTCTCGGGCGGCCTGCACGGCGTG
GGCGTGAGTTGCGTCAACGCGCTCAGCAAGATGCTGCGCCTGACGGTGCGGCGCGACGGC
AAGGTCCACGTGCTGGAGTTCAGCAAGGGCTTCGTGCAGAACCGCATCGTCGAGCAGGCG
GGCGGCGTCGAGGTCTCGCCGATGCGCATCATGGGCGACACCGAGAAGCGCGGTACCGAG
GTGCACTTCCTGCCCGACAACGAGATCTTCAAGGAAAACGCCGATTTCCACTACGAGATC
CTGGCCAAGCGCCTGCGCGAACTGAGCTTCCTGAACAACGGCGTGCGCATCCGCCTGAAG
GACGAGCGCAGCGGCAAGGAAGACGATTTCTCCGGCGCCGGCGGCGTGCAGGGTTTCGTC
GAATTCATCAACAAGGGCAAGACCATCCTGCACCCCACGCCCTTCTACGCCGCGGGCGAG
AAGCCGGCCGAGACGTACGGCGGCATCCCCGGCACGCACATCGGCGTGGAAGTGTCGATG
CAGTGGAACAGCGGCTACACCGAGCAGGTGCTCTGTTTCACCAACAACATCCCGCAGCGC
GACGGCGGCACCCACCTGACCGGCCTGCGCGCCGCGATGACCCGGATCATCAACAAGTAC
ATCGAAGAAAACGAGCTGGCCAAGAAGGCCAAGGTCGAGGTAACCGGCGACGACATGCGC
GAAGAAGAGGACATGGCCAAGCGCCGTTCGCGCCTGAAGGCGTTGATCACGCTCGGCAAG
ACCCGCGGCTACCTGACGCAGGTCGAGATCTCCGACCACCTGCCCGACAAGCTGGTCGAC
GCCGAGACGATGGAAGTCGTGATCGCCATGTTGAACGACATGGGCATCGCGGTCTACGAG
CAGACGCCCGACGCCGAGACGCTGTTCCAGAACAACGTCACCCCCACCGCCGCCACTGTC
GAGGAAGCCGAGGAAGAAGCCGAGGCCGCGCTCTCCACCGTCGACAGCGAATTCGGCCGC
ACCACCGACCCGGTCCGCATGTACATGCGCGAAATGGGCACCGTCGAGCTGCTGACCCGC
GAAGGCGAGATCGAGATCGCCAAGCGCATCGAAGGCGGCCTGCAGGCCATGATGGAGGCG
ATCAGCGCGTCGCCCGCCACCATCGCCGCGATCCTGGTGATGGGCGAGGACATCCGCGAA
GGCAAGGTCGTCATCTCGACCATCGTCGACGGCTTCTCCAACCCCAACGAAGCCGACGAC
TACGTGGCCGAGGAGGACTTCGACGAGTTCGACGAGGAAGACGACGACGACGGCAAGGGC
GGCTCCAAGGCGCTGACCAAGAAGCTGGAAGAATTGAAGCGCGAGGCGCTGTCGCGCTTC
GAC
>CFBP1942 Xylophilus ampelinus
CACGCCGGCGGCAAGTTCAACCAGAACAGCTACAAGGTCTCGGGCGGCCTGCACGGCGTG
GGCGTGAGTTGCGTCAACGCGCTCAGCAAGATGCTGCGCCTGACGGTGCGGCGCGACGGC
AAGGTCCACGTGCTGGAGTTCAGCAAGGGCTTCGTGCAGAACCGCATCGTCGAGCAGGCG
GGCGGCGTCGAGGTCTCGCCGATGCGCATCATGGGCGACACCGAGAAGCGCGGTACCGAG

GTGCACTTCCTGCCCGACAACGAGATCTTCAAGGAAAACGCCGATTTCCACTACGAGATC
CTGGCCAAGCGCCTGCGCGAACTGAGCTTCCTGAACAACGGCGTGCGCATCCGCCTGAAG
GACGAGCGCAGCGGCAAGGAAGACGATTTCTCCGGCGCCGGCGGCGTGCAGGGTTTCGTC
GAATTCATCAACAAGGGCAAGACCATCCTGCACCCCACGCCCTTCTACGCCGCGGGCGAG
AAGCCGGCCGAGACGTACGGCGGCATCCCCGGCACGCACATCGGCGTGGAAGTGTCGATG
CAGTGGAACAGCGGCTACACCGAGCAGGTGCTCTGTTTCACCAACAACATCCCGCAGCGC
GACGGCGGCACCCACCTGACCGGCCTGCGCGCCGCGATGACCCGGATCATCAACAAGTAC
ATCGAAGAAAACGAGCTGGCCAAGAAGGCCAAGGTCGAGGTAACCGGCGACGACATGCGC
GAAGAAGAGGACATGGCCAAGCGCCGTTCGCGCCTGAAGGCGTTGATCACGCTCGGCAAG
ACCCGCGGCTACCTGACGCAGGTCGAGATCTCCGACCACCTGCCCGACAAGCTGGTCGAC
GCCGAGACGATGGAAGTCGTGATCGCCATGTTGAACGACATGGGCATCGCGGTCTACGAG
CAGACGCCCGACGCCGAGACGCTGTTCCAGAACAACGTCACCCCCACCGCCGCCACTGTC
GAGGAAGCCGAGGAAGAAGCCGAGGCCGCGCTCTCCACCGTCGACAGCGAATTCGGCCGC
ACCACCGACCCGGTCCGCATGTACATGCGCGAAATGGGCACCGTCGAGCTGCTGACCCGC
GAAGGCGAGATCGAGATCGCCAAGCGCATCGAAGGCGGCCTGCAGGCCATGATGGAGGCG
ATCAGCGCGTCGCCCGCCACCATCGCCGCGATCCTGGTGATGGGCGAGGACATCCGCGAA
GGCAAGGTCGTCATCTCGACCATCGTCGACGGCTTCTCCAACCCCAACGAAGCCGACGAC
TACGTGGCCGAGGAGGACTTCGACGAGTTCGACGAGGAAGACGACGACGACGGCAAGGGC
GGCTCCAAGGCGCTGACCAAGAAGCTGGAAGAATTGAAGCGCGAGGCGCTGTCGCGCTTC
GAC
>CFBP2059 Xylophilus ampelinus
CACGCCGGCGGCAAGTTCAACCAGAACAGCTACAAGGTCTCGGGCGGCCTGCACGGCGTG
GGCGTGAGTTGCGTCAACGCGCTCAGCAAGATGCTGCGCCTGACGGTGCGGCGCGACGGC
AAGGTCCACGTGCTGGAGTTCAGCAAGGGCTTCGTGCAGAACCGCATCGTCGAGCAGGCG
GGCGGCGTCGAGGTCTCGCCGATGCGCATCATGGGCGACACCGAGAAGCGCGGTACCGAG
GTGCACTTCCTGCCCGACAACGAGATCTTCAAGGAAAACGCCGATTTCCACTACGAGATC
CTGGCCAAGCGCCTGCGCGAACTGAGCTTCCTGAACAACGGCGTGCGCATCCGCCTGAAG
GACGAGCGCAGCGGCAAGGAAGACGATTTCTCCGGCGCCGGCGGCGTGCAGGGTTTCGTC
GAATTCATCAACAAGGGCAAGACCATCCTGCACCCCACGCCCTTCTACGCCGCGGGCGAG
AAGCCGGCCGAGACGTACGGCGGCATCCCCGGCACGCACATCGGCGTGGAAGTGTCGATG
CAGTGGAACAGCGGCTACACCGAGCAGGTGCTCTGTTTCACCAACAACATCCCGCAGCGC
GACGGCGGCACCCACCTGACCGGCCTGCGCGCCGCGATGACCCGGATCATCAACAAGTAC
ATCGAAGAAAACGAGCTGGCCAAGAAGGCCAAGGTCGAGGTAACCGGCGACGACATGCGC
GAAGAAGAGGACATGGCCAAGCGCCGTTCGCGCCTGAAGGCGTTGATCACGCTCGGCAAG
ACCCGCGGCTACCTGACGCAGGTCGAGATCTCCGACCACCTGCCCGACAAGCTGGTCGAC
GCCGAGACGATGGAAGTCGTGATCGCCATGTTGAACGACATGGGCATCGCGGTCTACGAG
CAGACGCCCGACGCCGAGACGCTGTTCCAGAACAACGTCACCCCCACCGCCGCCACTGTC
GAGGAAGCCGAGGAAGAAGCCGAGGCCGCGCTCTCCACCGTCGACAGCGAATTCGGCCGC
ACCACCGACCCGGTCCGCATGTACATGCGCGAAATGGGCACCGTCGAGCTGCTGACCCGC
GAAGGCGAGATCGAGATCGCCAAGCGCATCGAAGGCGGCCTGCAGGCCATGATGGAGGCG
ATCAGCGCGTCGCCCGCCACCATCGCCGCGATCCTGGTGATGGGCGAGGACATCCGCGAA
GGCAAGGTCGTCATCTCGACCATCGTCGACGGCTTCTCCAACCCCAACGAAGCCGACGAC
TACGTGGCCGAGGAGGACTTCGACGAGTTCGACGAGGAAGACGACGACGACGGCAAGGGC
GGCTCCAAGGCGCTGACCAAGAAGCTGGAAGAATTGAAGCGCGAGGCGCTGTCGCGCTTC
GAC
>CFBP2060 Xylophilus ampelinus
CACGCCGGCGGCAAGTTCAACCAGAACAGCTACAAGGTCTCGGGCGGCCTGCACGGCGTG
GGCGTGAGTTGCGTCAACGCGCTCAGCAAGATGCTGCGCCTGACGGTGCGGCGCGACGGC
AAGGTCCACGTGCTGGAGTTCAGCAAGGGCTTCGTGCAGAACCGCATCGTCGAGCAGGCG
GGCGGCGTCGAGGTCTCGCCGATGCGCATCATGGGCGACACCGAGAAGCGCGGTACCGAG
GTGCACTTCCTGCCCGACAACGAGATCTTCAAGGAAAACGCCGATTTCCACTACGAGATC
CTGGCCAAGCGCCTGCGCGAACTGAGCTTCCTGAACAACGGCGTGCGCATCCGCCTGAAG
GACGAGCGCAGCGGCAAGGAAGACGATTTCTCCGGCGCCGGCGGCGTGCAGGGTTTCGTC
GAATTCATCAACAAGGGCAAGACCATCCTGCACCCCACGCCCTTCTACGCCGCGGGCGAG
AAGCCGGCCGAGACGTACGGCGGCATCCCCGGCACGCACATCGGCGTGGAAGTGTCGATG
CAGTGGAACAGCGGCTACACCGAGCAGGTGCTCTGTTTCACCAACAACATCCCGCAGCGC
GACGGCGGCACCCACCTGACCGGCCTGCGCGCCGCGATGACCCGGATCATCAACAAGTAC
ATCGAAGAAAACGAGCTGGCCAAGAAGGCCAAGGTCGAGGTAACCGGCGACGACATGCGC

GAAGAAGAGGACATGGCCAAGCGCCGTTCGCGCCTGAAGGCGTTGATCACGCTCGGCAAG
ACCCGCGGCTACCTGACGCAGGTCGAGATCTCCGACCACCTGCCCGACAAGCTGGTCGAC
GCCGAGACGATGGAAGTCGTGATCGCCATGTTGAACGACATGGGCATCGCGGTCTACGAG
CAGACGCCCGACGCCGAGACGCTGTTCCAGAACAACGTCACCCCCACCGCCGCCACTGTC
GAGGAAGCCGAGGAAGAAGCCGAGGCCGCGCTCTCCACCGTCGACAGCGAATTCGGCCGC
ACCACCGACCCGGTCCGCATGTACATGCGCGAAATGGGCACCGTCGAGCTGCTGACCCGC
GAAGGCGAGATCGAGATCGCCAAGCGCATCGAAGGCGGCCTGCAGGCCATGATGGAGGCG
ATCAGCGCGTCGCCCGCCACCATCGCCGCGATCCTGGTGATGGGCGAGGACATCCGCGAA
GGCAAGGTCGTCATCTCGACCATCGTCGACGGCTTCTCCAACCCCAACGAAGCCGACGAC
TACGTGGCCGAGGAGGACTTCGACGAGTTCGACGAGGAAGACGACGACGACGGCAAGGGC
GGCTCCAAGGCGCTGACCAAGAAGCTGGAAGAATTGAAGCGCGAGGCGCTGTCGCGCTTC
GAC
>CFBP2061 Xylophilus ampelinus
CACGCCGGCGGCAAGTTCAACCAGAACAGCTACAAGGTCTCGGGCGGCCTGCACGGCGTG
GGCGTGAGTTGCGTCAACGCGCTCAGCAAGATGCTGCGCCTGACGGTGCGGCGCGACGGC
AAGGTCCACGTGCTGGAGTTCAGCAAGGGCTTCGTGCAGAACCGCATCGTCGAGCAGGCG
GGCGGCGTCGAGGTCTCGCCGATGCGCATCATGGGCGACACCGAGAAGCGCGGTACCGAG
GTGCACTTCCTGCCCGACAACGAGATCTTCAAGGAAAACGCCGATTTCCACTACGAGATC
CTGGCCAAGCGCCTGCGCGAACTGAGCTTCCTGAACAACGGCGTGCGCATCCGCCTGAAG
GACGAGCGCAGCGGCAAGGAAGACGATTTCTCCGGCGCCGGCGGCGTGCAGGGTTTCGTC
GAATTCATCAACAAGGGCAAGACCATCCTGCACCCCACGCCCTTCTACGCCGCGGGCGAG
AAGCCGGCCGAGACGTACGGCGGCATCCCCGGCACGCACATCGGCGTGGAAGTGTCGATG
CAGTGGAACAGCGGCTACACCGAGCAGGTGCTCTGTTTCACCAACAACATCCCGCAGCGC
GACGGCGGCACCCACCTGACCGGCCTGCGCGCCGCGATGACCCGGATCATCAACAAGTAC
ATCGAAGAAAACGAGCTGGCCAAGAAGGCCAAGGTCGAGGTAACCGGCGACGACATGCGC
GAAGAAGAGGACATGGCCAAGCGCCGTTCGCGCCTGAAGGCGTTGATCACGCTCGGCAAG
ACCCGCGGCTACCTGACGCAGGTCGAGATCTCCGACCACCTGCCCGACAAGCTGGTCGAC
GCCGAGACGATGGAAGTCGTGATCGCCATGTTGAACGACATGGGCATCGCGGTCTACGAG
CAGACGCCCGACGCCGAGACGCTGTTCCAGAACAACGTCACCCCCACCGCCGCCACTGTC
GAGGAAGCCGAGGAAGAAGCCGAGGCCGCGCTCTCCACCGTCGACAGCGAATTCGGCCGC
ACCACCGACCCGGTCCGCATGTACATGCGCGAAATGGGCACCGTCGAGCTGCTGACCCGC
GAAGGCGAGATCGAGATCGCCAAGCGCATCGAAGGCGGCCTGCAGGCCATGATGGAGGCG
ATCAGCGCGTCGCCCGCCACCATCGCCGCGATCCTGGTGATGGGCGAGGACATCCGCGAA
GGCAAGGTCGTCATCTCGACCATCGTCGACGGCTTCTCCAACCCCAACGAAGCCGACGAC
TACGTGGCCGAGGAGGACTTCGACGAGTTCGACGAGGAAGACGACGACGACGGCAAGGGC
GGCTCCAAGGCGCTGACCAAGAAGCTGGAAGAATTGAAGCGCGAGGCGCTGTCGCGCTTC
GAC
>CFBP2098 Xylophilus ampelinus
CACGCCGGCGGCAAGTTCAACCAGAACAGCTACAAGGTCTCGGGCGGCCTGCACGGCGTG
GGCGTGAGTTGCGTCAACGCGCTCAGCAAGATGCTGCGCCTGACGGTGCGGCGCGACGGC
AAGGTCCACGTGCTGGAGTTCAGCAAGGGCTTCGTGCAGAACCGCATCGTCGAGCAGGCG
GGCGGCGTCGAGGTCTCGCCGATGCGCATCATGGGCGACACCGAGAAGCGCGGTACCGAG
GTGCACTTCCTGCCCGACAACGAGATCTTCAAGGAAAACGCCGATTTCCACTACGAGATC
CTGGCCAAGCGCCTGCGCGAACTGAGCTTCCTGAACAACGGCGTGCGCATCCGCCTGAAG
GACGAGCGCAGCGGCAAGGAAGACGATTTCTCCGGCGCCGGCGGCGTGCAGGGTTTCGTC
GAATTCATCAACAAGGGCAAGACCATCCTGCACCCCACGCCCTTCTACGCCGCGGGCGAG
AAGCCGGCCGAGACGTACGGCGGCATCCCCGGCACGCACATCGGCGTGGAAGTGTCGATG
CAGTGGAACAGCGGCTACACCGAGCAGGTGCTCTGTTTCACCAACAACATCCCGCAGCGC
GACGGCGGCACCCACCTGACCGGCCTGCGCGCCGCGATGACCCGGATCATCAACAAGTAC
ATCGAAGAAAACGAGCTGGCCAAGAAGGCCAAGGTCGAGGTAACCGGCGACGACATGCGC
GAAGAAGAGGACATGGCCAAGCGCCGTTCGCGCCTGAAGGCGTTGATCACGCTCGGCAAG
ACCCGCGGCTACCTGACGCAGGTCGAGATCTCCGACCACCTGCCCGACAAGCTGGTCGAC
GCCGAGACGATGGAAGTCGTGATCGCCATGTTGAACGACATGGGCATCGCGGTCTACGAG
CAGACGCCCGACGCCGAGACGCTGTTCCAGAACAACGTCACCCCCACCGCCGCCACTGTC
GAGGAAGCCGAGGAAGAAGCCGAGGCCGCGCTCTCCACCGTCGACAGCGAATTCGGCCGC
ACCACCGACCCGGTCCGCATGTACATGCGCGAAATGGGCACCGTCGAGCTGCTGACCCGC
GAAGGCGAGATCGAGATCGCCAAGCGCATCGAAGGCGGCCTGCAGGCCATGATGGAGGCG
ATCAGCGCGTCGCCCGCCACCATCGCCGCGATCCTGGTGATGGGCGAGGACATCCGCGAA

GGCAAGGTCGTCATCTCGACCATCGTCGACGGCTTCTCCAACCCCAACGAAGCCGACGAC
TACGTGGCCGAGGAGGACTTCGACGAGTTCGACGAGGAAGACGACGACGACGGCAAGGGC
GGCTCCAAGGCGCTGACCAAGAAGCTGGAAGAATTGAAGCGCGAGGCGCTGTCGCGCTTC
GAC
>CFBP2266 Xylophilus ampelinus
CACGCCGGCGGCAAGTTCAACCAGAACAGCTACAAGGTCTCGGGCGGCCTGCACGGCGTG
GGCGTGAGTTGCGTCAACGCGCTCAGCAAGATGCTGCGCCTGACGGTGCGGCGCGACGGC
AAGGTCCACGTGCTGGAGTTCAGCAAGGGCTTCGTGCAGAACCGCATCGTCGAGCAGGCG
GGCGGCGTCGAGGTCTCGCCGATGCGCATCATGGGCGACACCGAGAAGCGCGGTACCGAG
GTGCACTTCCTGCCCGACAACGAGATCTTCAAGGAAAACGCCGATTTCCACTACGAGATC
CTGGCCAAGCGCCTGCGCGAACTGAGCTTCCTGAACAACGGCGTGCGCATCCGCCTGAAG
GACGAGCGCAGCGGCAAGGAAGACGATTTCTCCGGCGCCGGCGGCGTGCAGGGTTTCGTC
GAATTCATCAACAAGGGCAAGACCATCCTGCACCCCACGCCCTTCTACGCCGCGGGCGAG
AAGCCGGCCGAGACGTACGGCGGCATCCCCGGCACGCACATCGGCGTGGAAGTGTCGATG
CAGTGGAACAGCGGCTACACCGAGCAGGTGCTCTGTTTCACCAACAACATCCCGCAGCGC
GACGGCGGCACCCACCTGACCGGCCTGCGCGCCGCGATGACCCGGATCATCAACAAGTAC
ATCGAAGAAAACGAGCTGGCCAAGAAGGCCAAGGTCGAGGTAACCGGCGACGACATGCGC
GAAGAAGAGGACATGGCCAAGCGCCGTTCGCGCCTGAAGGCGTTGATCACGCTCGGCAAG
ACCCGCGGCTACCTGACGCAGGTCGAGATCTCCGACCACCTGCCCGACAAGCTGGTCGAC
GCCGAGACGATGGAAGTCGTGATCGCCATGTTGAACGACATGGGCATCGCGGTCTACGAG
CAGACGCCCGACGCCGAGACGCTGTTCCAGAACAACGTCACCCCCACCGCCGCCACTGTC
GAGGAAGCCGAGGAAGAAGCCGAGGCCGCGCTCTCCACCGTCGACAGCGAATTCGGCCGC
ACCACCGACCCGGTCCGCATGTACATGCGCGAAATGGGCACCGTCGAGCTGCTGACCCGC
GAAGGCGAGATCGAGATCGCCAAGCGCATCGAAGGCGGCCTGCAGGCCATGATGGAGGCG
ATCAGCGCGTCGCCCGCCACCATCGCCGCGATCCTGGTGATGGGCGAGGACATCCGCGAA
GGCAAGGTCGTCATCTCGACCATCGTCGACGGCTTCTCCAACCCCAACGAAGCCGACGAC
TACGTGGCCGAGGAGGACTTCGACGAGTTCGACGAGGAAGACGACGACGACGGCAAGGGC
GGCTCCAAGGCGCTGACCAAGAAGCTGGAAGAATTGAAGCGCGAGGCGCTGTCGCGCTTC
GAC
>CFBP2289 Xylophilus ampelinus
CACGCCGGCGGCAAGTTCAACCAGAACAGCTACAAGGTCTCGGGCGGCCTGCACGGCGTG
GGCGTGAGTTGCGTCAACGCGCTCAGCAAGATGCTGCGCCTGACGGTGCGGCGCGACGGC
AAGGTCCACGTGCTGGAGTTCAGCAAGGGCTTCGTGCAGAACCGCATCGTCGAGCAGGCG
GGCGGCGTCGAGGTCTCGCCGATGCGCATCATGGGCGACACCGAGAAGCGCGGTACCGAG
GTGCACTTCCTGCCCGACAACGAGATCTTCAAGGAAAACGCCGATTTCCACTACGAGATC
CTGGCCAAGCGCCTGCGCGAACTGAGCTTCCTGAACAACGGCGTGCGCATCCGCCTGAAG
GACGAGCGCAGCGGCAAGGAAGACGATTTCTCCGGCGCCGGCGGCGTGCAGGGTTTCGTC
GAATTCATCAACAAGGGCAAGACCATCCTGCACCCCACGCCCTTCTACGCCGCGGGCGAG
AAGCCGGCCGAGACGTACGGCGGCATCCCCGGCACGCACATCGGCGTGGAAGTGTCGATG
CAGTGGAACAGCGGCTACACCGAGCAGGTGCTCTGTTTCACCAACAACATCCCGCAGCGC
GACGGCGGCACCCACCTGACCGGCCTGCGCGCCGCGATGACCCGGATCATCAACAAGTAC
ATCGAAGAAAACGAGCTGGCCAAGAAGGCCAAGGTCGAGGTAACCGGCGACGACATGCGC
GAAGAAGAGGACATGGCCAAGCGCCGTTCGCGCCTGAAGGCGTTGATCACGCTCGGCAAG
ACCCGCGGCTACCTGACGCAGGTCGAGATCTCCGACCACCTGCCCGACAAGCTGGTCGAC
GCCGAGACGATGGAAGTCGTGATCGCCATGTTGAACGACATGGGCATCGCGGTCTACGAG
CAGACGCCCGACGCCGAGACGCTGTTCCAGAACAACGTCACCCCCACCGCCGCCACTGTC
GAGGAAGCCGAGGAAGAAGCCGAGGCCGCGCTCTCCACCGTCGACAGCGAATTCGGCCGC
ACCACCGACCCGGTCCGCATGTACATGCGCGAAATGGGCACCGTCGAGCTGCTGACCCGC
GAAGGCGAGATCGAGATCGCCAAGCGCATCGAAGGCGGCCTGCAGGCCATGATGGAGGCG
ATCAGCGCGTCGCCCGCCACCATCGCCGCGATCCTGGTGATGGGCGAGGACATCCGCGAA
GGCAAGGTCGTCATCTCGACCATCGTCGACGGCTTCTCCAACCCCAACGAAGCCGACGAC
TACGTGGCCGAGGAGGACTTCGACGAGTTCGACGAGGAAGACGACGACGACGGCAAGGGC
GGCTCCAAGGCGCTGACCAAGAAGCTGGAAGAATTGAAGCGCGAGGCGCTGTCGCGCTTC
GAC
>CFBP2291 Xylophilus ampelinus
CACGCCGGCGGCAAGTTCAACCAGAACAGCTACAAGGTCTCGGGCGGCCTGCACGGCGTG
GGCGTGAGTTGCGTCAACGCGCTCAGCAAGATGCTGCGCCTGACGGTGCGGCGCGACGGC
AAGGTCCACGTGCTGGAGTTCAGCAAGGGCTTCGTGCAGAACCGCATCGTCGAGCAGGCG

GGCGGCGTCGAGGTCTCGCCGATGCGCATCATGGGCGACACCGAGAAGCGCGGTACCGAG
GTGCACTTCCTGCCCGACAACGAGATCTTCAAGGAAAACGCCGATTTCCACTACGAGATC
CTGGCCAAGCGCCTGCGCGAACTGAGCTTCCTGAACAACGGCGTGCGCATCCGCCTGAAG
GACGAGCGCAGCGGCAAGGAAGACGATTTCTCCGGCGCCGGCGGCGTGCAGGGTTTCGTC
GAATTCATCAACAAGGGCAAGACCATCCTGCACCCCACGCCCTTCTACGCCGCGGGCGAG
AAGCCGGCCGAGACGTACGGCGGCATCCCCGGCACGCACATCGGCGTGGAAGTGTCGATG
CAGTGGAACAGCGGCTACACCGAGCAGGTGCTCTGTTTCACCAACAACATCCCGCAGCGC
GACGGCGGCACCCACCTGACCGGCCTGCGCGCCGCGATGACCCGGATCATCAACAAGTAC
ATCGAAGAAAACGAGCTGGCCAAGAAGGCCAAGGTCGAGGTAACCGGCGACGACATGCGC
GAAGAAGAGGACATGGCCAAGCGCCGTTCGCGCCTGAAGGCGTTGATCACGCTCGGCAAG
ACCCGCGGCTACCTGACGCAGGTCGAGATCTCCGACCACCTGCCCGACAAGCTGGTCGAC
GCCGAGACGATGGAAGTCGTGATCGCCATGTTGAACGACATGGGCATCGCGGTCTACGAG
CAGACGCCCGACGCCGAGACGCTGTTCCAGAACAACGTCACCCCCACCGCCGCCACTGTC
GAGGAAGCCGAGGAAGAAGCCGAGGCCGCGCTCTCCACCGTCGACAGCGAATTCGGCCGC
ACCACCGACCCGGTCCGCATGTACATGCGCGAAATGGGCACCGTCGAGCTGCTGACCCGC
GAAGGCGAGATCGAGATCGCCAAGCGCATCGAAGGCGGCCTGCAGGCCATGATGGAGGCG
ATCAGCGCGTCGCCCGCCACCATCGCCGCGATCCTGGTGATGGGCGAGGACATCCGCGAA
GGCAAGGTCGTCATCTCGACCATCGTCGACGGCTTCTCCAACCCCAACGAAGCCGACGAC
TACGTGGCCGAGGAGGACTTCGACGAGTTCGACGAGGAAGACGACGACGACGGCAAGGGC
GGCTCCAAGGCGCTGACCAAGAAGCTGGAAGAATTGAAGCGCGAGGCGCTGTCGCGCTTC
GAC
>CFBP2292 Xylophilus ampelinus
CACGCCGGCGGCAAGTTCAACCAGAACAGCTACAAGGTCTCGGGCGGCCTGCACGGCGTG
GGCGTGAGTTGCGTCAACGCGCTCAGCAAGATGCTGCGCCTGACGGTGCGGCGCGACGGC
AAGGTCCACGTGCTGGAGTTCAGCAAGGGCTTCGTGCAGAACCGCATCGTCGAGCAGGCG
GGCGGCGTCGAGGTCTCGCCGATGCGCATCATGGGCGACACCGAGAAGCGCGGTACCGAG
GTGCACTTCCTGCCCGACAACGAGATCTTCAAGGAAAACGCCGATTTCCACTACGAGATC
CTGGCCAAGCGCCTGCGCGAACTGAGCTTCCTGAACAACGGCGTGCGCATCCGCCTGAAG
GACGAGCGCAGCGGCAAGGAAGACGATTTCTCCGGCGCCGGCGGCGTGCAGGGTTTCGTC
GAATTCATCAACAAGGGCAAGACCATCCTGCACCCCACGCCCTTCTACGCCGCGGGCGAG
AAGCCGGCCGAGACGTACGGCGGCATCCCCGGCACGCACATCGGCGTGGAAGTGTCGATG
CAGTGGAACAGCGGCTACACCGAGCAGGTGCTCTGTTTCACCAACAACATCCCGCAGCGC
GACGGCGGCACCCACCTGACCGGCCTGCGCGCCGCGATGACCCGGATCATCAACAAGTAC
ATCGAAGAAAACGAGCTGGCCAAGAAGGCCAAGGTCGAGGTAACCGGCGACGACATGCGC
GAAGAAGAGGACATGGCCAAGCGCCGTTCGCGCCTGAAGGCGTTGATCACGCTCGGCAAG
ACCCGCGGCTACCTGACGCAGGTCGAGATCTCCGACCACCTGCCCGACAAGCTGGTCGAC
GCCGAGACGATGGAAGTCGTGATCGCCATGTTGAACGACATGGGCATCGCGGTCTACGAG
CAGACGCCCGACGCCGAGACGCTGTTCCAGAACAACGTCACCCCCACCGCCGCCACTGTC
GAGGAAGCCGAGGAAGAAGCCGAGGCCGCGCTCTCCACCGTCGACAGCGAATTCGGCCGC
ACCACCGACCCGGTCCGCATGTACATGCGCGAAATGGGCACCGTCGAGCTGCTGACCCGC
GAAGGCGAGATCGAGATCGCCAAGCGCATCGAAGGCGGCCTGCAGGCCATGATGGAGGCG
ATCAGCGCGTCGCCCGCCACCATCGCCGCGATCCTGGTGATGGGCGAGGACATCCGCGAA
GGCAAGGTCGTCATCTCGACCATCGTCGACGGCTTCTCCAACCCCAACGAAGCCGACGAC
TACGTGGCCGAGGAGGACTTCGACGAGTTCGACGAGGAAGACGACGACGACGGCAAGGGC
GGCTCCAAGGCGCTGACCAAGAAGCTGGAAGAATTGAAGCGCGAGGCGCTGTCGCGCTTC
GAC
>CFBP2293 Xylophilus ampelinus
CACGCCGGCGGCAAGTTCAACCAGAACAGCTACAAGGTCTCGGGCGGCCTGCACGGCGTG
GGCGTGAGTTGCGTCAACGCGCTCAGCAAGATGCTGCGCCTGACGGTGCGGCGCGACGGC
AAGGTCCACGTGCTGGAGTTCAGCAAGGGCTTCGTGCAGAACCGCATCGTCGAGCAGGCG
GGCGGCGTCGAGGTCTCGCCGATGCGCATCATGGGCGACACCGAGAAGCGCGGTACCGAG
GTGCACTTCCTGCCCGACAACGAGATCTTCAAGGAAAACGCCGATTTCCACTACGAGATC
CTGGCCAAGCGCCTGCGCGAACTGAGCTTCCTGAACAACGGCGTGCGCATCCGCCTGAAG
GACGAGCGCAGCGGCAAGGAAGACGATTTCTCCGGCGCCGGCGGCGTGCAGGGTTTCGTC
GAATTCATCAACAAGGGCAAGACCATCCTGCACCCCACGCCCTTCTACGCCGCGGGCGAG
AAGCCGGCCGAGACGTACGGCGGCATCCCCGGCACGCACATCGGCGTGGAAGTGTCGATG
CAGTGGAACAGCGGCTACACCGAGCAGGTGCTCTGTTTCACCAACAACATCCCGCAGCGC
GACGGCGGCACCCACCTGACCGGCCTGCGCGCCGCGATGACCCGGATCATCAACAAGTAC

ATCGAAGAAAACGAGCTGGCCAAGAAGGCCAAGGTCGAGGTAACCGGCGACGACATGCGC
GAAGAAGAGGACATGGCCAAGCGCCGTTCGCGCCTGAAGGCGTTGATCACGCTCGGCAAG
ACCCGCGGCTACCTGACGCAGGTCGAGATCTCCGACCACCTGCCCGACAAGCTGGTCGAC
GCCGAGACGATGGAAGTCGTGATCGCCATGTTGAACGACATGGGCATCGCGGTCTACGAG
CAGACGCCCGACGCCGAGACGCTGTTCCAGAACAACGTCACCCCCACCGCCGCCACTGTC
GAGGAAGCCGAGGAAGAAGCCGAGGCCGCGCTCTCCACCGTCGACAGCGAATTCGGCCGC
ACCACCGACCCGGTCCGCATGTACATGCGCGAAATGGGCACCGTCGAGCTGCTGACCCGC
GAAGGCGAGATCGAGATCGCCAAGCGCATCGAAGGCGGCCTGCAGGCCATGATGGAGGCG
ATCAGCGCGTCGCCCGCCACCATCGCCGCGATCCTGGTGATGGGCGAGGACATCCGCGAA
GGCAAGGTCGTCATCTCGACCATCGTCGACGGCTTCTCCAACCCCAACGAAGCCGACGAC
TACGTGGCCGAGGAGGACTTCGACGAGTTCGACGAGGAAGACGACGACGACGGCAAGGGC
GGCTCCAAGGCGCTGACCAAGAAGCTGGAAGAATTGAAGCGCGAGGCGCTGTCGCGCTTC
GAC
>CFBP2294 Xylophilus ampelinus
CACGCCGGCGGCAAGTTCAACCAGAACAGCTACAAGGTCTCGGGCGGCCTGCACGGCGTG
GGCGTGAGTTGCGTCAACGCGCTCAGCAAGATGCTGCGCCTGACGGTGCGGCGCGACGGC
AAGGTCCACGTGCTGGAGTTCAGCAAGGGCTTCGTGCAGAACCGCATCGTCGAGCAGGCG
GGCGGCGTCGAGGTCTCGCCGATGCGCATCATGGGCGACACCGAGAAGCGCGGTACCGAG
GTGCACTTCCTGCCCGACAACGAGATCTTCAAGGAAAACGCCGATTTCCACTACGAGATC
CTGGCCAAGCGCCTGCGCGAACTGAGCTTCCTGAACAACGGCGTGCGCATCCGCCTGAAG
GACGAGCGCAGCGGCAAGGAAGACGATTTCTCCGGCGCCGGCGGCGTGCAGGGTTTCGTC
GAATTCATCAACAAGGGCAAGACCATCCTGCACCCCACGCCCTTCTACGCCGCGGGCGAG
AAGCCGGCCGAGACGTACGGCGGCATCCCCGGCACGCACATCGGCGTGGAAGTGTCGATG
CAGTGGAACAGCGGCTACACCGAGCAGGTGCTCTGTTTCACCAACAACATCCCGCAGCGC
GACGGCGGCACCCACCTGACCGGCCTGCGCGCCGCGATGACCCGGATCATCAACAAGTAC
ATCGAAGAAAACGAGCTGGCCAAGAAGGCCAAGGTCGAGGTAACCGGCGACGACATGCGC
GAAGAAGAGGACATGGCCAAGCGCCGTTCGCGCCTGAAGGCGTTGATCACGCTCGGCAAG
ACCCGCGGCTACCTGACGCAGGTCGAGATCTCCGACCACCTGCCCGACAAGCTGGTCGAC
GCCGAGACGATGGAAGTCGTGATCGCCATGTTGAACGACATGGGCATCGCGGTCTACGAG
CAGACGCCCGACGCCGAGACGCTGTTCCAGAACAACGTCACCCCCACCGCCGCCACTGTC
GAGGAAGCCGAGGAAGAAGCCGAGGCCGCGCTCTCCACCGTCGACAGCGAATTCGGCCGC
ACCACCGACCCGGTCCGCATGTACATGCGCGAAATGGGCACCGTCGAGCTGCTGACCCGC
GAAGGCGAGATCGAGATCGCCAAGCGCATCGAAGGCGGCCTGCAGGCCATGATGGAGGCG
ATCAGCGCGTCGCCCGCCACCATCGCCGCGATCCTGGTGATGGGCGAGGACATCCGCGAA
GGCAAGGTCGTCATCTCGACCATCGTCGACGGCTTCTCCAACCCCAACGAAGCCGACGAC
TACGTGGCCGAGGAGGACTTCGACGAGTTCGACGAGGAAGACGACGACGACGGCAAGGGC
GGCTCCAAGGCGCTGACCAAGAAGCTGGAAGAATTGAAGCGCGAGGCGCTGTCGCGCTTC
GAC
>CFBP2295 Xylophilus ampelinus
CACGCCGGCGGCAAGTTCAACCAGAACAGCTACAAGGTCTCGGGCGGCCTGCACGGCGTG
GGCGTGAGTTGCGTCAACGCGCTCAGCAAGATGCTGCGCCTGACGGTGCGGCGCGACGGC
AAGGTCCACGTGCTGGAGTTCAGCAAGGGCTTCGTGCAGAACCGCATCGTCGAGCAGGCG
GGCGGCGTCGAGGTCTCGCCGATGCGCATCATGGGCGACACCGAGAAGCGCGGTACCGAG
GTGCACTTCCTGCCCGACAACGAGATCTTCAAGGAAAACGCCGATTTCCACTACGAGATC
CTGGCCAAGCGCCTGCGCGAACTGAGCTTCCTGAACAACGGCGTGCGCATCCGCCTGAAG
GACGAGCGCAGCGGCAAGGAAGACGATTTCTCCGGCGCCGGCGGCGTGCAGGGTTTCGTC
GAATTCATCAACAAGGGCAAGACCATCCTGCACCCCACGCCCTTCTACGCCGCGGGCGAG
AAGCCGGCCGAGACGTACGGCGGCATCCCCGGCACGCACATCGGCGTGGAAGTGTCGATG
CAGTGGAACAGCGGCTACACCGAGCAGGTGCTCTGTTTCACCAACAACATCCCGCAGCGC
GACGGCGGCACCCACCTGACCGGCCTGCGCGCCGCGATGACCCGGATCATCAACAAGTAC
ATCGAAGAAAACGAGCTGGCCAAGAAGGCCAAGGTCGAGGTAACCGGCGACGACATGCGC
GAAGAAGAGGACATGGCCAAGCGCCGTTCGCGCCTGAAGGCGTTGATCACGCTCGGCAAG
ACCCGCGGCTACCTGACGCAGGTCGAGATCTCCGACCACCTGCCCGACAAGCTGGTCGAC
GCCGAGACGATGGAAGTCGTGATCGCCATGTTGAACGACATGGGCATCGCGGTCTACGAG
CAGACGCCCGACGCCGAGACGCTGTTCCAGAACAACGTCACCCCCACCGCCGCCACTGTC
GAGGAAGCCGAGGAAGAAGCCGAGGCCGCGCTCTCCACCGTCGACAGCGAATTCGGCCGC
ACCACCGACCCGGTCCGCATGTACATGCGCGAAATGGGCACCGTCGAGCTGCTGACCCGC
GAAGGCGAGATCGAGATCGCCAAGCGCATCGAAGGCGGCCTGCAGGCCATGATGGAGGCG

ATCAGCGCGTCGCCCGCCACCATCGCCGCGATCCTGGTGATGGGCGAGGACATCCGCGAA
GGCAAGGTCGTCATCTCGACCATCGTCGACGGCTTCTCCAACCCCAACGAAGCCGACGAC
TACGTGGCCGAGGAGGACTTCGACGAGTTCGACGAGGAAGACGACGACGACGGCAAGGGC
GGCTCCAAGGCGCTGACCAAGAAGCTGGAAGAATTGAAGCGCGAGGCGCTGTCGCGCTTC
GAC
>CFBP2358 Xylophilus ampelinus
CACGCCGGCGGCAAGTTCAACCAGAACAGCTACAAGGTCTCGGGCGGCCTGCACGGCGTG
GGCGTGAGTTGCGTCAACGCGCTCAGCAAGATGCTGCGCCTGACGGTGCGGCGCGACGGC
AAGGTCCACGTGCTGGAGTTCAGCAAGGGCTTCGTGCAGAACCGCATCGTCGAGCAGGCG
GGCGGCGTCGAGGTCTCGCCGATGCGCATCATGGGCGACACCGAGAAGCGCGGTACCGAG
GTGCACTTCCTGCCCGACAACGAGATCTTCAAGGAAAACGCCGATTTCCACTACGAGATC
CTGGCCAAGCGCCTGCGCGAACTGAGCTTCCTGAACAACGGCGTGCGCATCCGCCTGAAG
GACGAGCGCAGCGGCAAGGAAGACGATTTCTCCGGCGCCGGCGGCGTGCAGGGTTTCGTC
GAATTCATCAACAAGGGCAAGACCATCCTGCACCCCACGCCCTTCTACGCCGCGGGCGAG
AAGCCGGCCGAGACGTACGGCGGCATCCCCGGCACGCACATCGGCGTGGAAGTGTCGATG
CAGTGGAACAGCGGCTACACCGAGCAGGTGCTCTGTTTCACCAACAACATCCCGCAGCGC
GACGGCGGCACCCACCTGACCGGCCTGCGCGCCGCGATGACCCGGATCATCAACAAGTAC
ATCGAAGAAAACGAGCTGGCCAAGAAGGCCAAGGTCGAGGTAACCGGCGACGACATGCGC
GAAGAAGAGGACATGGCCAAGCGCCGTTCGCGCCTGAAGGCGTTGATCACGCTCGGCAAG
ACCCGCGGCTACCTGACGCAGGTCGAGATCTCCGACCACCTGCCCGACAAGCTGGTCGAC
GCCGAGACGATGGAAGTCGTGATCGCCATGTTGAACGACATGGGCATCGCGGTCTACGAG
CAGACGCCCGACGCCGAGACGCTGTTCCAGAACAACGTCACCCCCACCGCCGCCACTGTC
GAGGAAGCCGAGGAAGAAGCCGAGGCCGCGCTCTCCACCGTCGACAGCGAATTCGGCCGC
ACCACCGACCCGGTCCGCATGTACATGCGCGAAATGGGCACCGTCGAGCTGCTGACCCGC
GAAGGCGAGATCGAGATCGCCAAGCGCATCGAAGGCGGCCTGCAGGCCATGATGGAGGCG
ATCAGCGCGTCGCCCGCCACCATCGCCGCGATCCTGGTGATGGGCGAGGACATCCGCGAA
GGCAAGGTCGTCATCTCGACCATCGTCGACGGCTTCTCCAACCCCAACGAAGCCGACGAC
TACGTGGCCGAGGAGGACTTCGACGAGTTCGACGAGGAAGACGACGACGACGGCAAGGGC
GGCTCCAAGGCGCTGACCAAGAAGCTGGAAGAATTGAAGCGCGAGGCGCTGTCGCGCTTC
GAC
>CFBP2393 Xylophilus ampelinus
CACGCCGGCGGCAAGTTCAACCAGAACAGCTACAAGGTCTCGGGCGGCCTGCACGGCGTG
GGCGTGAGTTGCGTCAACGCGCTCAGCAAGATGCTGCGCCTGACGGTGCGGCGCGACGGC
AAGGTCCACGTGCTGGAGTTCAGCAAGGGCTTCGTGCAGAACCGCATCGTCGAGCAGGCG
GGCGGCGTCGAGGTCTCGCCGATGCGCATCATGGGCGACACCGAGAAGCGCGGTACCGAG
GTGCACTTCCTGCCCGACAACGAGATCTTCAAGGAAAACGCCGATTTCCACTACGAGATC
CTGGCCAAGCGCCTGCGCGAACTGAGCTTCCTGAACAACGGCGTGCGCATCCGCCTGAAG
GACGAGCGCAGCGGCAAGGAAGACGATTTCTCCGGCGCCGGCGGCGTGCAGGGTTTCGTC
GAATTCATCAACAAGGGCAAGACCATCCTGCACCCCACGCCCTTCTACGCCGCGGGCGAG
AAGCCGGCCGAGACGTACGGCGGCATCCCCGGCACGCACATCGGCGTGGAAGTGTCGATG
CAGTGGAACAGCGGCTACACCGAGCAGGTGCTCTGTTTCACCAACAACATCCCGCAGCGC
GACGGCGGCACCCACCTGACCGGCCTGCGCGCCGCGATGACCCGGATCATCAACAAGTAC
ATCGAAGAAAACGAGCTGGCCAAGAAGGCCAAGGTCGAGGTAACCGGCGACGACATGCGC
GAAGAAGAGGACATGGCCAAGCGCCGTTCGCGCCTGAAGGCGTTGATCACGCTCGGCAAG
ACCCGCGGCTACCTGACGCAGGTCGAGATCTCCGACCACCTGCCCGACAAGCTGGTCGAC
GCCGAGACGATGGAAGTCGTGATCGCCATGTTGAACGACATGGGCATCGCGGTCTACGAG
CAGACGCCCGACGCCGAGACGCTGTTCCAGAACAACGTCACCCCCACCGCCGCCACTGTC
GAGGAAGCCGAGGAAGAAGCCGAGGCCGCGCTCTCCACCGTCGACAGCGAATTCGGCCGC
ACCACCGACCCGGTCCGCATGTACATGCGCGAAATGGGCACCGTCGAGCTGCTGACCCGC
GAAGGCGAGATCGAGATCGCCAAGCGCATCGAAGGCGGCCTGCAGGCCATGATGGAGGCG
ATCAGCGCGTCGCCCGCCACCATCGCCGCGATCCTGGTGATGGGCGAGGACATCCGCGAA
GGCAAGGTCGTCATCTCGACCATCGTCGACGGCTTCTCCAACCCCAACGAAGCCGACGAC
TACGTGGCCGAGGAGGACTTCGACGAGTTCGACGAGGAAGACGACGACGACGGCAAGGGC
GGCTCCAAGGCGCTGACCAAGAAGCTGGAAGAATTGAAGCGCGAGGCGCTGTCGCGCTTC
GAC
>CFBP2394 Xylophilus ampelinus
CACGCCGGCGGCAAGTTCAACCAGAACAGCTACAAGGTCTCGGGCGGCCTGCACGGCGTG
GGCGTGAGTTGCGTCAACGCGCTCAGCAAGATGCTGCGCCTGACGGTGCGGCGCGACGGC

AAGGTCCACGTGCTGGAGTTCAGCAAGGGCTTCGTGCAGAACCGCATCGTCGAGCAGGCG
GGCGGCGTCGAGGTCTCGCCGATGCGCATCATGGGCGACACCGAGAAGCGCGGTACCGAG
GTGCACTTCCTGCCCGACAACGAGATCTTCAAGGAAAACGCCGATTTCCACTACGAGATC
CTGGCCAAGCGCCTGCGCGAACTGAGCTTCCTGAACAACGGCGTGCGCATCCGCCTGAAG
GACGAGCGCAGCGGCAAGGAAGACGATTTCTCCGGCGCCGGCGGCGTGCAGGGTTTCGTC
GAATTCATCAACAAGGGCAAGACCATCCTGCACCCCACGCCCTTCTACGCCGCGGGCGAG
AAGCCGGCCGAGACGTACGGCGGCATCCCCGGCACGCACATCGGCGTGGAAGTGTCGATG
CAGTGGAACAGCGGCTACACCGAGCAGGTGCTCTGTTTCACCAACAACATCCCGCAGCGC
GACGGCGGCACCCACCTGACCGGCCTGCGCGCCGCGATGACCCGGATCATCAACAAGTAC
ATCGAAGAAAACGAGCTGGCCAAGAAGGCCAAGGTCGAGGTAACCGGCGACGACATGCGC
GAAGAAGAGGACATGGCCAAGCGCCGTTCGCGCCTGAAGGCGTTGATCACGCTCGGCAAG
ACCCGCGGCTACCTGACGCAGGTCGAGATCTCCGACCACCTGCCCGACAAGCTGGTCGAC
GCCGAGACGATGGAAGTCGTGATCGCCATGTTGAACGACATGGGCATCGCGGTCTACGAG
CAGACGCCCGACGCCGAGACGCTGTTCCAGAACAACGTCACCCCCACCGCCGCCACTGTC
GAGGAAGCCGAGGAAGAAGCCGAGGCCGCGCTCTCCACCGTCGACAGCGAATTCGGCCGC
ACCACCGACCCGGTCCGCATGTACATGCGCGAAATGGGCACCGTCGAGCTGCTGACCCGC
GAAGGCGAGATCGAGATCGCCAAGCGCATCGAAGGCGGCCTGCAGGCCATGATGGAGGCG
ATCAGCGCGTCGCCCGCCACCATCGCCGCGATCCTGGTGATGGGCGAGGACATCCGCGAA
GGCAAGGTCGTCATCTCGACCATCGTCGACGGCTTCTCCAACCCCAACGAAGCCGACGAC
TACGTGGCCGAGGAGGACTTCGACGAGTTCGACGAGGAAGACGACGACGACGGCAAGGGC
GGCTCCAAGGCGCTGACCAAGAAGCTGGAAGAATTGAAGCGCGAGGCGCTGTCGCGCTTC
GAC
>CFBP2399 Xylophilus ampelinus
CACGCCGGCGGCAAGTTCAACCAGAACAGCTACAAGGTCTCGGGCGGCCTGCACGGCGTG
GGCGTGAGTTGCGTCAACGCGCTCAGCAAGATGCTGCGCCTGACGGTGCGGCGCGACGGC
AAGGTCCACGTGCTGGAGTTCAGCAAGGGCTTCGTGCAGAACCGCATCGTCGAGCAGGCG
GGCGGCGTCGAGGTCTCGCCGATGCGCATCATGGGCGACACCGAGAAGCGCGGTACCGAG
GTGCACTTCCTGCCCGACAACGAGATCTTCAAGGAAAACGCCGATTTCCACTACGAGATC
CTGGCCAAGCGCCTGCGCGAACTGAGCTTCCTGAACAACGGCGTGCGCATCCGCCTGAAG
GACGAGCGCAGCGGCAAGGAAGACGATTTCTCCGGCGCCGGCGGCGTGCAGGGTTTCGTC
GAATTCATCAACAAGGGCAAGACCATCCTGCACCCCACGCCCTTCTACGCCGCGGGCGAG
AAGCCGGCCGAGACGTACGGCGGCATCCCCGGCACGCACATCGGCGTGGAAGTGTCGATG
CAGTGGAACAGCGGCTACACCGAGCAGGTGCTCTGTTTCACCAACAACATCCCGCAGCGC
GACGGCGGCACCCACCTGACCGGCCTGCGCGCCGCGATGACCCGGATCATCAACAAGTAC
ATCGAAGAAAACGAGCTGGCCAAGAAGGCCAAGGTCGAGGTAACCGGCGACGACATGCGC
GAAGAAGAGGACATGGCCAAGCGCCGTTCGCGCCTGAAGGCGTTGATCACGCTCGGCAAG
ACCCGCGGCTACCTGACGCAGGTCGAGATCTCCGACCACCTGCCCGACAAGCTGGTCGAC
GCCGAGACGATGGAAGTCGTGATCGCCATGTTGAACGACATGGGCATCGCGGTCTACGAG
CAGACGCCCGACGCCGAGACGCTGTTCCAGAACAACGTCACCCCCACCGCCGCCACTGTC
GAGGAAGCCGAGGAAGAAGCCGAGGCCGCGCTCTCCACCGTCGACAGCGAATTCGGCCGC
ACCACCGACCCGGTCCGCATGTACATGCGCGAAATGGGCACCGTCGAGCTGCTGACCCGC
GAAGGCGAGATCGAGATCGCCAAGCGCATCGAAGGCGGCCTGCAGGCCATGATGGAGGCG
ATCAGCGCGTCGCCCGCCACCATCGCCGCGATCCTGGTGATGGGCGAGGACATCCGCGAA
GGCAAGGTCGTCATCTCGACCATCGTCGACGGCTTCTCCAACCCCAACGAAGCCGACGAC
TACGTGGCCGAGGAGGACTTCGACGAGTTCGACGAGGAAGACGACGACGACGGCAAGGGC
GGCTCCAAGGCGCTGACCAAGAAGCTGGAAGAATTGAAGCGCGAGGCGCTGTCGCGCTTC
GAC
>CFBP2400 Xylophilus ampelinus
CACGCCGGCGGCAAGTTCAACCAGAACAGCTACAAGGTCTCGGGCGGCCTGCACGGCGTG
GGCGTGAGTTGCGTCAACGCGCTCAGCAAGATGCTGCGCCTGACGGTGCGGCGCGACGGC
AAGGTCCACGTGCTGGAGTTCAGCAAGGGCTTCGTGCAGAACCGCATCGTCGAGCAGGCG
GGCGGCGTCGAGGTCTCGCCGATGCGCATCATGGGCGACACCGAGAAGCGCGGTACCGAG
GTGCACTTCCTGCCCGACAACGAGATCTTCAAGGAAAACGCCGATTTCCACTACGAGATC
CTGGCCAAGCGCCTGCGCGAACTGAGCTTCCTGAACAACGGCGTGCGCATCCGCCTGAAG
GACGAGCGCAGCGGCAAGGAAGACGATTTCTCCGGCGCCGGCGGCGTGCAGGGTTTCGTC
GAATTCATCAACAAGGGCAAGACCATCCTGCACCCCACGCCCTTCTACGCCGCGGGCGAG
AAGCCGGCCGAGACGTACGGCGGCATCCCCGGCACGCACATCGGCGTGGAAGTGTCGATG
CAGTGGAACAGCGGCTACACCGAGCAGGTGCTCTGTTTCACCAACAACATCCCGCAGCGC

GACGGCGGCACCCACCTGACCGGCCTGCGCGCCGCGATGACCCGGATCATCAACAAGTAC
ATCGAAGAAAACGAGCTGGCCAAGAAGGCCAAGGTCGAGGTAACCGGCGACGACATGCGC
GAAGAAGAGGACATGGCCAAGCGCCGTTCGCGCCTGAAGGCGTTGATCACGCTCGGCAAG
ACCCGCGGCTACCTGACGCAGGTCGAGATCTCCGACCACCTGCCCGACAAGCTGGTCGAC
GCCGAGACGATGGAAGTCGTGATCGCCATGTTGAACGACATGGGCATCGCGGTCTACGAG
CAGACGCCCGACGCCGAGACGCTGTTCCAGAACAACGTCACCCCCACCGCCGCCACTGTC
GAGGAAGCCGAGGAAGAAGCCGAGGCCGCGCTCTCCACCGTCGACAGCGAATTCGGCCGC
ACCACCGACCCGGTCCGCATGTACATGCGCGAAATGGGCACCGTCGAGCTGCTGACCCGC
GAAGGCGAGATCGAGATCGCCAAGCGCATCGAAGGCGGCCTGCAGGCCATGATGGAGGCG
ATCAGCGCGTCGCCCGCCACCATCGCCGCGATCCTGGTGATGGGCGAGGACATCCGCGAA
GGCAAGGTCGTCATCTCGACCATCGTCGACGGCTTCTCCAACCCCAACGAAGCCGACGAC
TACGTGGCCGAGGAGGACTTCGACGAGTTCGACGAGGAAGACGACGACGACGGCAAGGGC
GGCTCCAAGGCGCTGACCAAGAAGCTGGAAGAATTGAAGCGCGAGGCGCTGTCGCGCTTC
GAC
>CFBP2730 Xylophilus ampelinus
CACGCCGGCGGCAAGTTCAACCAGAACAGCTACAAGGTCTCGGGCGGCCTGCACGGCGTG
GGCGTGAGTTGCGTCAACGCGCTCAGCAAGATGCTGCGCCTGACGGTGCGGCGCGACGGC
AAGGTCCACGTGCTGGAGTTCAGCAAGGGCTTCGTGCAGAACCGCATCGTCGAGCAGGCG
GGCGGCGTCGAGGTCTCGCCGATGCGCATCATGGGCGACACCGAGAAGCGCGGTACCGAG
GTGCACTTCCTGCCCGACAACGAGATCTTCAAGGAAAACGCCGATTTCCACTACGAGATC
CTGGCCAAGCGCCTGCGCGAACTGAGCTTCCTGAACAACGGCGTGCGCATCCGCCTGAAG
GACGAGCGCAGCGGCAAGGAAGACGATTTCTCCGGCGCCGGCGGCGTGCAGGGTTTCGTC
GAATTCATCAACAAGGGCAAGACCATCCTGCACCCCACGCCCTTCTACGCCGCGGGCGAG
AAGCCGGCCGAGACGTACGGCGGCATCCCCGGCACGCACATCGGCGTGGAAGTGTCGATG
CAGTGGAACAGCGGCTACACCGAGCAGGTGCTCTGTTTCACCAACAACATCCCGCAGCGC
GACGGCGGCACCCACCTGACCGGCCTGCGCGCCGCGATGACCCGGATCATCAACAAGTAC
ATCGAAGAAAACGAGCTGGCCAAGAAGGCCAAGGTCGAGGTAACCGGCGACGACATGCGC
GAAGAAGAGGACATGGCCAAGCGCCGTTCGCGCCTGAAGGCGTTGATCACGCTCGGCAAG
ACCCGCGGCTACCTGACGCAGGTCGAGATCTCCGACCACCTGCCCGACAAGCTGGTCGAC
GCCGAGACGATGGAAGTCGTGATCGCCATGTTGAACGACATGGGCATCGCGGTCTACGAG
CAGACGCCCGACGCCGAGACGCTGTTCCAGAACAACGTCACCCCCACCGCCGCCACTGTC
GAGGAAGCCGAGGAAGAAGCCGAGGCCGCGCTCTCCACCGTCGACAGCGAATTCGGCCGC
ACCACCGACCCGGTCCGCATGTACATGCGCGAAATGGGCACCGTCGAGCTGCTGACCCGC
GAAGGCGAGATCGAGATCGCCAAGCGCATCGAAGGCGGCCTGCAGGCCATGATGGAGGCG
ATCAGCGCGTCGCCCGCCACCATCGCCGCGATCCTGGTGATGGGCGAGGACATCCGCGAA
GGCAAGGTCGTCATCTCGACCATCGTCGACGGCTTCTCCAACCCCAACGAAGCCGACGAC
TACGTGGCCGAGGAGGACTTCGACGAGTTCGACGAGGAAGACGACGACGACGGCAAGGGC
GGCTCCAAGGCGCTGACCAAGAAGCTGGAAGAATTGAAGCGCGAGGCGCTGTCGCGCTTC
GAC
>CFBP2731 Xylophilus ampelinus
CACGCCGGCGGCAAGTTCAACCAGAACAGCTACAAGGTCTCGGGCGGCCTGCACGGCGTG
GGCGTGAGTTGCGTCAACGCGCTCAGCAAGATGCTGCGCCTGACGGTGCGGCGCGACGGC
AAGGTCCACGTGCTGGAGTTCAGCAAGGGCTTCGTGCAGAACCGCATCGTCGAGCAGGCG
GGCGGCGTCGAGGTCTCGCCGATGCGCATCATGGGCGACACCGAGAAGCGCGGTACCGAG
GTGCACTTCCTGCCCGACAACGAGATCTTCAAGGAAAACGCCGATTTCCACTACGAGATC
CTGGCCAAGCGCCTGCGCGAACTGAGCTTCCTGAACAACGGCGTGCGCATCCGCCTGAAG
GACGAGCGCAGCGGCAAGGAAGACGATTTCTCCGGCGCCGGCGGCGTGCAGGGTTTCGTC
GAATTCATCAACAAGGGCAAGACCATCCTGCACCCCACGCCCTTCTACGCCGCGGGCGAG
AAGCCGGCCGAGACGTACGGCGGCATCCCCGGCACGCACATCGGCGTGGAAGTGTCGATG
CAGTGGAACAGCGGCTACACCGAGCAGGTGCTCTGTTTCACCAACAACATCCCGCAGCGC
GACGGCGGCACCCACCTGACCGGCCTGCGCGCCGCGATGACCCGGATCATCAACAAGTAC
ATCGAAGAAAACGAGCTGGCCAAGAAGGCCAAGGTCGAGGTAACCGGCGACGACATGCGC
GAAGAAGAGGACATGGCCAAGCGCCGTTCGCGCCTGAAGGCGTTGATCACGCTCGGCAAG
ACCCGCGGCTACCTGACGCAGGTCGAGATCTCCGACCACCTGCCCGACAAGCTGGTCGAC
GCCGAGACGATGGAAGTCGTGATCGCCATGTTGAACGACATGGGCATCGCGGTCTACGAG
CAGACGCCCGACGCCGAGACGCTGTTCCAGAACAACGTCACCCCCACCGCCGCCACTGTC
GAGGAAGCCGAGGAAGAAGCCGAGGCCGCGCTCTCCACCGTCGACAGCGAATTCGGCCGC
ACCACCGACCCGGTCCGCATGTACATGCGCGAAATGGGCACCGTCGAGCTGCTGACCCGC

GAAGGCGAGATCGAGATCGCCAAGCGCATCGAAGGCGGCCTGCAGGCCATGATGGAGGCG
ATCAGCGCGTCGCCCGCCACCATCGCCGCGATCCTGGTGATGGGCGAGGACATCCGCGAA
GGCAAGGTCGTCATCTCGACCATCGTCGACGGCTTCTCCAACCCCAACGAAGCCGACGAC
TACGTGGCCGAGGAGGACTTCGACGAGTTCGACGAGGAAGACGACGACGACGGCAAGGGC
GGCTCCAAGGCGCTGACCAAGAAGCTGGAAGAATTGAAGCGCGAGGCGCTGTCGCGCTTC
GAC
>CFBP2748 Xylophilus ampelinus
CACGCCGGCGGCAAGTTCAACCAGAACAGCTACAAGGTCTCGGGCGGCCTGCACGGCGTG
GGCGTGAGTTGCGTCAACGCGCTCAGCAAGATGCTGCGCCTGACGGTGCGGCGCGACGGC
AAGGTCCACGTGCTGGAGTTCAGCAAGGGCTTCGTGCAGAACCGCATCGTCGAGCAGGCG
GGCGGCGTCGAGGTCTCGCCGATGCGCATCATGGGCGACACCGAGAAGCGCGGTACCGAG
GTGCACTTCCTGCCCGACAACGAGATCTTCAAGGAAAACGCCGATTTCCACTACGAGATC
CTGGCCAAGCGCCTGCGCGAACTGAGCTTCCTGAACAACGGCGTGCGCATCCGCCTGAAG
GACGAGCGCAGCGGCAAGGAAGACGATTTCTCCGGCGCCGGCGGCGTGCAGGGTTTCGTC
GAATTCATCAACAAGGGCAAGACCATCCTGCACCCCACGCCCTTCTACGCCGCGGGCGAG
AAGCCGGCCGAGACGTACGGCGGCATCCCCGGCACGCACATCGGCGTGGAAGTGTCGATG
CAGTGGAACAGCGGCTACACCGAGCAGGTGCTCTGTTTCACCAACAACATCCCGCAGCGC
GACGGCGGCACCCACCTGACCGGCCTGCGCGCCGCGATGACCCGGATCATCAACAAGTAC
ATCGAAGAAAACGAGCTGGCCAAGAAGGCCAAGGTCGAGGTAACCGGCGACGACATGCGC
GAAGAAGAGGACATGGCCAAGCGCCGTTCGCGCCTGAAGGCGTTGATCACGCTCGGCAAG
ACCCGCGGCTACCTGACGCAGGTCGAGATCTCCGACCACCTGCCCGACAAGCTGGTCGAC
GCCGAGACGATGGAAGTCGTGATCGCCATGTTGAACGACATGGGCATCGCGGTCTACGAG
CAGACGCCCGACGCCGAGACGCTGTTCCAGAACAACGTCACCCCCACCGCCGCCACTGTC
GAGGAAGCCGAGGAAGAAGCCGAGGCCGCGCTCTCCACCGTCGACAGCGAATTCGGCCGC
ACCACCGACCCGGTCCGCATGTACATGCGCGAAATGGGCACCGTCGAGCTGCTGACCCGC
GAAGGCGAGATCGAGATCGCCAAGCGCATCGAAGGCGGCCTGCAGGCCATGATGGAGGCG
ATCAGCGCGTCGCCCGCCACCATCGCCGCGATCCTGGTGATGGGCGAGGACATCCGCGAA
GGCAAGGTCGTCATCTCGACCATCGTCGACGGCTTCTCCAACCCCAACGAAGCCGACGAC
TACGTGGCCGAGGAGGACTTCGACGAGTTCGACGAGGAAGACGACGACGACGGCAAGGGC
GGCTCCAAGGCGCTGACCAAGAAGCTGGAAGAATTGAAGCGCGAGGCGCTGTCGCGCTTC
GAC
>CFBP2875 Xylophilus ampelinus
CACGCCGGCGGCAAGTTCAACCAGAACAGCTACAAGGTCTCGGGCGGCCTGCACGGCGTG
GGCGTGAGTTGCGTCAACGCGCTCAGCAAGATGCTGCGCCTGACGGTGCGGCGCGACGGC
AAGGTCCACGTGCTGGAGTTCAGCAAGGGCTTCGTGCAGAACCGCATCGTCGAGCAGGCG
GGCGGCGTCGAGGTCTCGCCGATGCGCATCATGGGCGACACCGAGAAGCGCGGTACCGAG
GTGCACTTCCTGCCCGACAACGAGATCTTCAAGGAAAACGCCGATTTCCACTACGAGATC
CTGGCCAAGCGCCTGCGCGAACTGAGCTTCCTGAACAACGGCGTGCGCATCCGCCTGAAG
GACGAGCGCAGCGGCAAGGAAGACGATTTCTCCGGCGCCGGCGGCGTGCAGGGTTTCGTC
GAATTCATCAACAAGGGCAAGACCATCCTGCACCCCACGCCCTTCTACGCCGCGGGCGAG
AAGCCGGCCGAGACGTACGGCGGCATCCCCGGCACGCACATCGGCGTGGAAGTGTCGATG
CAGTGGAACAGCGGCTACACCGAGCAGGTGCTCTGTTTCACCAACAACATCCCGCAGCGC
GACGGCGGCACCCACCTGACCGGCCTGCGCGCCGCGATGACCCGGATCATCAACAAGTAC
ATCGAAGAAAACGAGCTGGCCAAGAAGGCCAAGGTCGAGGTAACCGGCGACGACATGCGC
GAAGAAGAGGACATGGCCAAGCGCCGTTCGCGCCTGAAGGCGTTGATCACGCTCGGCAAG
ACCCGCGGCTACCTGACGCAGGTCGAGATCTCCGACCACCTGCCCGACAAGCTGGTCGAC
GCCGAGACGATGGAAGTCGTGATCGCCATGTTGAACGACATGGGCATCGCGGTCTACGAG
CAGACGCCCGACGCCGAGACGCTGTTCCAGAACAACGTCACCCCCACCGCCGCCACTGTC
GAGGAAGCCGAGGAAGAAGCCGAGGCCGCGCTCTCCACCGTCGACAGCGAATTCGGCCGC
ACCACCGACCCGGTCCGCATGTACATGCGCGAAATGGGCACCGTCGAGCTGCTGACCCGC
GAAGGCGAGATCGAGATCGCCAAGCGCATCGAAGGCGGCCTGCAGGCCATGATGGAGGCG
ATCAGCGCGTCGCCCGCCACCATCGCCGCGATCCTGGTGATGGGCGAGGACATCCGCGAA
GGCAAGGTCGTCATCTCGACCATCGTCGACGGCTTCTCCAACCCCAACGAAGCCGACGAC
TACGTGGCCGAGGAGGACTTCGACGAGTTCGACGAGGAAGACGACGACGACGGCAAGGGC
GGCTCCAAGGCGCTGACCAAGAAGCTGGAAGAATTGAAGCGCGAGGCGCTGTCGCGCTTC
GAC
>CFBP2876 Xylophilus ampelinus
CACGCCGGCGGCAAGTTCAACCAGAACAGCTACAAGGTCTCGGGCGGCCTGCACGGCGTG

GGCGTGAGTTGCGTCAACGCGCTCAGCAAGATGCTGCGCCTGACGGTGCGGCGCGACGGC
AAGGTCCACGTGCTGGAGTTCAGCAAGGGCTTCGTGCAGAACCGCATCGTCGAGCAGGCG
GGCGGCGTCGAGGTCTCGCCGATGCGCATCATGGGCGACACCGAGAAGCGCGGTACCGAG
GTGCACTTCCTGCCCGACAACGAGATCTTCAAGGAAAACGCCGATTTCCACTACGAGATC
CTGGCCAAGCGCCTGCGCGAACTGAGCTTCCTGAACAACGGCGTGCGCATCCGCCTGAAG
GACGAGCGCAGCGGCAAGGAAGACGATTTCTCCGGCGCCGGCGGCGTGCAGGGTTTCGTC
GAATTCATCAACAAGGGCAAGACCATCCTGCACCCCACGCCCTTCTACGCCGCGGGCGAG
AAGCCGGCCGAGACGTACGGCGGCATCCCCGGCACGCACATCGGCGTGGAAGTGTCGATG
CAGTGGAACAGCGGCTACACCGAGCAGGTGCTCTGTTTCACCAACAACATCCCGCAGCGC
GACGGCGGCACCCACCTGACCGGCCTGCGCGCCGCGATGACCCGGATCATCAACAAGTAC
ATCGAAGAAAACGAGCTGGCCAAGAAGGCCAAGGTCGAGGTAACCGGCGACGACATGCGC
GAAGAAGAGGACATGGCCAAGCGCCGTTCGCGCCTGAAGGCGTTGATCACGCTCGGCAAG
ACCCGCGGCTACCTGACGCAGGTCGAGATCTCCGACCACCTGCCCGACAAGCTGGTCGAC
GCCGAGACGATGGAAGTCGTGATCGCCATGTTGAACGACATGGGCATCGCGGTCTACGAG
CAGACGCCCGACGCCGAGACGCTGTTCCAGAACAACGTCACCCCCACCGCCGCCACTGTC
GAGGAAGCCGAGGAAGAAGCCGAGGCCGCGCTCTCCACCGTCGACAGCGAATTCGGCCGC
ACCACCGACCCGGTCCGCATGTACATGCGCGAAATGGGCACCGTCGAGCTGCTGACCCGC
GAAGGCGAGATCGAGATCGCCAAGCGCATCGAAGGCGGCCTGCAGGCCATGATGGAGGCG
ATCAGCGCGTCGCCCGCCACCATCGCCGCGATCCTGGTGATGGGCGAGGACATCCGCGAA
GGCAAGGTCGTCATCTCGACCATCGTCGACGGCTTCTCCAACCCCAACGAAGCCGACGAC
TACGTGGCCGAGGAGGACTTCGACGAGTTCGACGAGGAAGACGACGACGACGGCAAGGGC
GGCTCCAAGGCGCTGACCAAGAAGCTGGAAGAATTGAAGCGCGAGGCGCTGTCGCGCTTC
GAC
>CFBP2877 Xylophilus ampelinus
CACGCCGGCGGCAAGTTCAACCAGAACAGCTACAAGGTCTCGGGCGGCCTGCACGGCGTG
GGCGTGAGTTGCGTCAACGCGCTCAGCAAGATGCTGCGCCTGACGGTGCGGCGCGACGGC
AAGGTCCACGTGCTGGAGTTCAGCAAGGGCTTCGTGCAGAACCGCATCGTCGAGCAGGCG
GGCGGCGTCGAGGTCTCGCCGATGCGCATCATGGGCGACACCGAGAAGCGCGGTACCGAG
GTGCACTTCCTGCCCGACAACGAGATCTTCAAGGAAAACGCCGATTTCCACTACGAGATC
CTGGCCAAGCGCCTGCGCGAACTGAGCTTCCTGAACAACGGCGTGCGCATCCGCCTGAAG
GACGAGCGCAGCGGCAAGGAAGACGATTTCTCCGGCGCCGGCGGCGTGCAGGGTTTCGTC
GAATTCATCAACAAGGGCAAGACCATCCTGCACCCCACGCCCTTCTACGCCGCGGGCGAG
AAGCCGGCCGAGACGTACGGCGGCATCCCCGGCACGCACATCGGCGTGGAAGTGTCGATG
CAGTGGAACAGCGGCTACACCGAGCAGGTGCTCTGTTTCACCAACAACATCCCGCAGCGC
GACGGCGGCACCCACCTGACCGGCCTGCGCGCCGCGATGACCCGGATCATCAACAAGTAC
ATCGAAGAAAACGAGCTGGCCAAGAAGGCCAAGGTCGAGGTAACCGGCGACGACATGCGC
GAAGAAGAGGACATGGCCAAGCGCCGTTCGCGCCTGAAGGCGTTGATCACGCTCGGCAAG
ACCCGCGGCTACCTGACGCAGGTCGAGATCTCCGACCACCTGCCCGACAAGCTGGTCGAC
GCCGAGACGATGGAAGTCGTGATCGCCATGTTGAACGACATGGGCATCGCGGTCTACGAG
CAGACGCCCGACGCCGAGACGCTGTTCCAGAACAACGTCACCCCCACCGCCGCCACTGTC
GAGGAAGCCGAGGAAGAAGCCGAGGCCGCGCTCTCCACCGTCGACAGCGAATTCGGCCGC
ACCACCGACCCGGTCCGCATGTACATGCGCGAAATGGGCACCGTCGAGCTGCTGACCCGC
GAAGGCGAGATCGAGATCGCCAAGCGCATCGAAGGCGGCCTGCAGGCCATGATGGAGGCG
ATCAGCGCGTCGCCCGCCACCATCGCCGCGATCCTGGTGATGGGCGAGGACATCCGCGAA
GGCAAGGTCGTCATCTCGACCATCGTCGACGGCTTCTCCAACCCCAACGAAGCCGACGAC
TACGTGGCCGAGGAGGACTTCGACGAGTTCGACGAGGAAGACGACGACGACGGCAAGGGC
GGCTCCAAGGCGCTGACCAAGAAGCTGGAAGAATTGAAGCGCGAGGCGCTGTCGCGCTTC
GAC
>CFBP3478 Xylophilus ampelinus
CACGCCGGCGGCAAGTTCAACCAGAACAGCTACAAGGTCTCGGGCGGCCTGCACGGCGTG
GGCGTGAGTTGCGTCAACGCGCTCAGCAAGATGCTGCGCCTGACGGTGCGGCGCGACGGC
AAGGTCCACGTGCTGGAGTTCAGCAAGGGCTTCGTGCAGAACCGCATCGTCGAGCAGGCG
GGCGGCGTCGAGGTCTCGCCGATGCGCATCATGGGCGACACCGAGAAGCGCGGTACCGAG
GTGCACTTCCTGCCCGACAACGAGATCTTCAAGGAAAACGCCGATTTCCACTACGAGATC
CTGGCCAAGCGCCTGCGCGAACTGAGCTTCCTGAACAACGGCGTGCGCATCCGCCTGAAG
GACGAGCGCAGCGGCAAGGAAGACGATTTCTCCGGCGCCGGCGGCGTGCAGGGTTTCGTC
GAATTCATCAACAAGGGCAAGACCATCCTGCACCCCACGCCCTTCTACGCCGCGGGCGAG
AAGCCGGCCGAGACGTACGGCGGCATCCCCGGCACGCACATCGGCGTGGAAGTGTCGATG

CAGTGGAACAGCGGCTACACCGAGCAGGTGCTCTGTTTCACCAACAACATCCCGCAGCGC
GACGGCGGCACCCACCTGACCGGCCTGCGCGCCGCGATGACCCGGATCATCAACAAGTAC
ATCGAAGAAAACGAGCTGGCCAAGAAGGCCAAGGTCGAGGTAACCGGCGACGACATGCGC
GAAGAAGAGGACATGGCCAAGCGCCGTTCGCGCCTGAAGGCGTTGATCACGCTCGGCAAG
ACCCGCGGCTACCTGACGCAGGTCGAGATCTCCGACCACCTGCCCGACAAGCTGGTCGAC
GCCGAGACGATGGAAGTCGTGATCGCCATGTTGAACGACATGGGCATCGCGGTCTACGAG
CAGACGCCCGACGCCGAGACGCTGTTCCAGAACAACGTCACCCCCACCGCCGCCACTGTC
GAGGAAGCCGAGGAAGAAGCCGAGGCCGCGCTCTCCACCGTCGACAGCGAATTCGGCCGC
ACCACCGACCCGGTCCGCATGTACATGCGCGAAATGGGCACCGTCGAGCTGCTGACCCGC
GAAGGCGAGATCGAGATCGCCAAGCGCATCGAAGGCGGCCTGCAGGCCATGATGGAGGCG
ATCAGCGCGTCGCCCGCCACCATCGCCGCGATCCTGGTGATGGGCGAGGACATCCGCGAA
GGCAAGGTCGTCATCTCGACCATCGTCGACGGCTTCTCCAACCCCAACGAAGCCGACGAC
TACGTGGCCGAGGAGGACTTCGACGAGTTCGACGAGGAAGACGACGACGACGGCAAGGGC
GGCTCCAAGGCGCTGACCAAGAAGCTGGAAGAATTGAAGCGCGAGGCGCTGTCGCGCTTC
GAC
>CFBP3674T Xylophilus ampelinus
CACGCCGGCGGCAAGTTCAACCAGAACAGCTACAAGGTCTCGGGCGGCCTGCACGGCGTG
GGCGTGAGTTGCGTCAACGCGCTCAGCAAGATGCTGCGCCTGACGGTGCGGCGCGACGGC
AAGGTCCACGTGCTGGAGTTCAGCAAGGGCTTCGTGCAGAACCGCATCGTCGAGCAGGCG
GGCGGCGTCGAGGTCTCGCCGATGCGCATCATGGGCGACACCGAGAAGCGCGGTACCGAG
GTGCACTTCCTGCCCGACAACGAGATCTTCAAGGAAAACGCCGATTTCCACTACGAGATC
CTGGCCAAGCGCCTGCGCGAACTGAGCTTCCTGAACAACGGCGTGCGCATCCGCCTGAAG
GACGAGCGCAGCGGCAAGGAAGACGATTTCTCCGGCGCCGGCGGCGTGCAGGGTTTCGTC
GAATTCATCAACAAGGGCAAGACCATCCTGCACCCCACGCCCTTCTACGCCGCGGGCGAG
AAGCCGGCCGAGACGTACGGCGGCATCCCCGGCACGCACATCGGCGTGGAAGTGTCGATG
CAGTGGAACAGCGGCTACACCGAGCAGGTGCTCTGTTTCACCAACAACATCCCGCAGCGC
GACGGCGGCACCCACCTGACCGGCCTGCGCGCCGCGATGACCCGGATCATCAACAAGTAC
ATCGAAGAAAACGAGCTGGCCAAGAAGGCCAAGGTCGAGGTAACCGGCGACGACATGCGC
GAAGAAGAGGACATGGCCAAGCGCCGTTCGCGCCTGAAGGCGTTGATCACGCTCGGCAAG
ACCCGCGGCTACCTGACGCAGGTCGAGATCTCCGACCACCTGCCCGACAAGCTGGTCGAC
GCCGAGACGATGGAAGTCGTGATCGCCATGTTGAACGACATGGGCATCGCGGTCTACGAG
CAGACGCCCGACGCCGAGACGCTGTTCCAGAACAACGTCACCCCCACCGCCGCCACTGTC
GAGGAAGCCGAGGAAGAAGCCGAGGCCGCGCTCTCCACCGTCGACAGCGAATTCGGCCGC
ACCACCGACCCGGTCCGCATGTACATGCGCGAAATGGGCACCGTCGAGCTGCTGACCCGC
GAAGGCGAGATCGAGATCGCCAAGCGCATCGAAGGCGGCATGCAGGCCATGATGGAGGCG
ATCAGCGCGTCGCCCGCCACCATCGCCGCGATCCTGGTGATGGGCGAGGACATCCGCGAA
GGCAAGGTCGTCATCTCGACCATCGTCGACGGCTTCTCCAACCCCAACGAAGCCGACGAC
TACGTGGCCGAGGAGGACTTCGACGAGTTCGACGAGGAAGACGACGACGACGGCAAGGGC
GGCTCCAAGGCGCTGACCAAGAAGCTGGAAGAATTGAAGCGCGAGGCGCTGTCGCGCTTC
GAC
>CFBP3675 Xylophilus ampelinus
CACGCCGGCGGCAAGTTCAACCAGAACAGCTACAAGGTCTCGGGCGGCCTGCACGGCGTG
GGCGTGAGTTGCGTCAACGCGCTCAGCAAGATGCTGCGCCTGACGGTGCGGCGCGACGGC
AAGGTCCACGTGCTGGAGTTCAGCAAGGGCTTCGTGCAGAACCGCATCGTCGAGCAGGCG
GGCGGCGTCGAGGTCTCGCCGATGCGCATCATGGGCGACACCGAGAAGCGCGGTACCGAG
GTGCACTTCCTGCCCGACAACGAGATCTTCAAGGAAAACGCCGATTTCCACTACGAGATC
CTGGCCAAGCGCCTGCGCGAACTGAGCTTCCTGAACAACGGCGTGCGCATCCGCCTGAAG
GACGAGCGCAGCGGCAAGGAAGACGATTTCTCCGGCGCCGGCGGCGTGCAGGGTTTCGTC
GAATTCATCAACAAGGGCAAGACCATCCTGCACCCCACGCCCTTCTACGCCGCGGGCGAG
AAGCCGGCCGAGACGTACGGCGGCATCCCCGGCACGCACATCGGCGTGGAAGTGTCGATG
CAGTGGAACAGCGGCTACACCGAGCAGGTGCTCTGTTTCACCAACAACATCCCGCAGCGC
GACGGCGGCACCCACCTGACCGGCCTGCGCGCCGCGATGACCCGGATCATCAACAAGTAC
ATCGAAGAAAACGAGCTGGCCAAGAAGGCCAAGGTCGAGGTAACCGGCGACGACATGCGC
GAAGAAGAGGACATGGCCAAGCGCCGTTCGCGCCTGAAGGCGTTGATCACGCTCGGCAAG
ACCCGCGGCTACCTGACGCAGGTCGAGATCTCCGACCACCTGCCCGACAAGCTGGTCGAC
GCCGAGACGATGGAAGTCGTGATCGCCATGTTGAACGACATGGGCATCGCGGTCTACGAG
CAGACGCCCGACGCCGAGACGCTGTTCCAGAACAACGTCACCCCCACCGCCGCCACTGTC
GAGGAAGCCGAGGAAGAAGCCGAGGCCGCGCTCTCCACCGTCGACAGCGAATTCGGCCGC

ACCACCGACCCGGTCCGCATGTACATGCGCGAAATGGGCACCGTCGAGCTGCTGACCCGC
GAAGGCGAGATCGAGATCGCCAAGCGCATCGAAGGCGGCATGCAGGCCATGATGGAGGCG
ATCAGCGCGTCGCCCGCCACCATCGCCGCGATCCTGGTGATGGGCGAGGACATCCGCGAA
GGCAAGGTCGTCATCTCGACCATCGTCGACGGCTTCTCCAACCCCAACGAAGCCGACGAC
TACGTGGCCGAGGAGGACTTCGACGAGTTCGACGAGGAAGACGACGACGACGGCAAGGGC
GGCTCCAAGGCGCTGACCAAGAAGCTGGAAGAATTGAAGCGCGAGGCGCTGTCGCGCTTC
GAC
>CFBP3677 Xylophilus ampelinus
CACGCCGGCGGCAAGTTCAACCAGAACAGCTACAAGGTCTCGGGCGGCCTGCACGGCGTG
GGCGTGAGTTGCGTCAACGCGCTCAGCAAGATGCTGCGCCTGACGGTGCGGCGCGACGGC
AAGGTCCACGTGCTGGAGTTCAGCAAGGGCTTCGTGCAGAACCGCATCGTCGAGCAGGCG
GGCGGCGTCGAGGTCTCGCCGATGCGCATCATGGGCGACACCGAGAAGCGCGGTACCGAG
GTGCACTTCCTGCCCGACAACGAGATCTTCAAGGAAAACGCCGATTTCCACTACGAGATC
CTGGCCAAGCGCCTGCGCGAACTGAGCTTCCTGAACAACGGCGTGCGCATCCGCCTGAAG
GACGAGCGCAGCGGCAAGGAAGACGATTTCTCCGGCGCCGGCGGCGTGCAGGGTTTCGTC
GAATTCATCAACAAGGGCAAGACCATCCTGCACCCCACGCCCTTCTACGCCGCGGGCGAG
AAGCCGGCCGAGACGTACGGCGGCATCCCCGGCACGCACATCGGCGTGGAAGTGTCGATG
CAGTGGAACAGCGGCTACACCGAGCAGGTGCTCTGTTTCACCAACAACATCCCGCAGCGC
GACGGCGGCACCCACCTGACCGGCCTGCGCGCCGCGATGACCCGGATCATCAACAAGTAC
ATCGAAGAAAACGAGCTGGCCAAGAAGGCCAAGGTCGAGGTAACCGGCGACGACATGCGC
GAAGAAGAGGACATGGCCAAGCGCCGTTCGCGCCTGAAGGCGTTGATCACGCTCGGCAAG
ACCCGCGGCTACCTGACGCAGGTCGAGATCTCCGACCACCTGCCCGACAAGCTGGTCGAC
GCCGAGACGATGGAAGTCGTGATCGCCATGTTGAACGACATGGGCATCGCGGTCTACGAG
CAGACGCCCGACGCCGAGACGCTGTTCCAGAACAACGTCACCCCCACCGCCGCCACTGTC
GAGGAAGCCGAGGAAGAAGCCGAGGCCGCGCTCTCCACCGTCGACAGCGAATTCGGCCGC
ACCACCGACCCGGTCCGCATGTACATGCGCGAAATGGGCACCGTCGAGCTGCTGACCCGC
GAAGGCGAGATCGAGATCGCCAAGCGCATCGAAGGCGGCATGCAGGCCATGATGGAGGCG
ATCAGCGCGTCGCCCGCCACCATCGCCGCGATCCTGGTGATGGGCGAGGACATCCGCGAA
GGCAAGGTCGTCATCTCGACCATCGTCGACGGCTTCTCCAACCCCAACGAAGCCGACGAC
TACGTGGCCGAGGAGGACTTCGACGAGTTCGACGAGGAAGACGACGACGACGGCAAGGGC
GGCTCCAAGGCGCTGACCAAGAAGCTGGAAGAATTGAAGCGCGAGGCGCTGTCGCGCTTC
GAC
>CFBP3678 Xylophilus ampelinus
CACGCCGGCGGCAAGTTCAACCAGAACAGCTACAAGGTCTCGGGCGGCCTGCACGGCGTG
GGCGTGAGTTGCGTCAACGCGCTCAGCAAGATGCTGCGCCTGACGGTGCGGCGCGACGGC
AAGGTCCACGTGCTGGAGTTCAGCAAGGGCTTCGTGCAGAACCGCATCGTCGAGCAGGCG
GGCGGCGTCGAGGTCTCGCCGATGCGCATCATGGGCGACACCGAGAAGCGCGGTACCGAG
GTGCACTTCCTGCCCGACAACGAGATCTTCAAGGAAAACGCCGATTTCCACTACGAGATC
CTGGCCAAGCGCCTGCGCGAACTGAGCTTCCTGAACAACGGCGTGCGCATCCGCCTGAAG
GACGAGCGCAGCGGCAAGGAAGACGATTTCTCCGGCGCCGGCGGCGTGCAGGGTTTCGTC
GAATTCATCAACAAGGGCAAGACCATCCTGCACCCCACGCCCTTCTACGCCGCGGGCGAG
AAGCCGGCCGAGACGTACGGCGGCATCCCCGGCACGCACATCGGCGTGGAAGTGTCGATG
CAGTGGAACAGCGGCTACACCGAGCAGGTGCTCTGTTTCACCAACAACATCCCGCAGCGC
GACGGCGGCACCCACCTGACCGGCCTGCGCGCCGCGATGACCCGGATCATCAACAAGTAC
ATCGAAGAAAACGAGCTGGCCAAGAAGGCCAAGGTCGAGGTAACCGGCGACGACATGCGC
GAAGAAGAGGACATGGCCAAGCGCCGTTCGCGCCTGAAGGCGTTGATCACGCTCGGCAAG
ACCCGCGGCTACCTGACGCAGGTCGAGATCTCCGACCACCTGCCCGACAAGCTGGTCGAC
GCCGAGACGATGGAAGTCGTGATCGCCATGTTGAACGACATGGGCATCGCGGTCTACGAG
CAGACGCCCGACGCCGAGACGCTGTTCCAGAACAACGTCACCCCCACCGCCGCCACTGTC
GAGGAAGCCGAGGAAGAAGCCGAGGCCGCGCTCTCCACCGTCGACAGCGAATTCGGCCGC
ACCACCGACCCGGTCCGCATGTACATGCGCGAAATGGGCACCGTCGAGCTGCTGACCCGC
GAAGGCGAGATCGAGATCGCCAAGCGCATCGAAGGCGGCATGCAGGCCATGATGGAGGCG
ATCAGCGCGTCGCCCGCCACCATCGCCGCGATCCTGGTGATGGGCGAGGACATCCGCGAA
GGCAAGGTCGTCATCTCGACCATCGTCGACGGCTTCTCCAACCCCAACGAAGCCGACGAC
TACGTGGCCGAGGAGGACTTCGACGAGTTCGACGAGGAAGACGACGACGACGGCAAGGGC
GGCTCCAAGGCGCTGACCAAGAAGCTGGAAGAATTGAAGCGCGAGGCGCTGTCGCGCTTC
GAC
>CFBP3679 Xylophilus ampelinus

CACGCCGGCGGCAAGTTCAACCAGAACAGCTACAAGGTCTCGGGCGGCCTGCACGGCGTG
GGCGTGAGTTGCGTCAACGCGCTCAGCAAGATGCTGCGCCTGACGGTGCGGCGCGACGGC
AAGGTCCACGTGCTGGAGTTCAGCAAGGGCTTCGTGCAGAACCGCATCGTCGAGCAGGCG
GGCGGCGTCGAGGTCTCGCCGATGCGCATCATGGGCGACACCGAGAAGCGCGGTACCGAG
GTGCACTTCCTGCCCGACAACGAGATCTTCAAGGAAAACGCCGATTTCCACTACGAGATC
CTGGCCAAGCGCCTGCGCGAACTGAGCTTCCTGAACAACGGCGTGCGCATCCGCCTGAAG
GACGAGCGCAGCGGCAAGGAAGACGATTTCTCCGGCGCCGGCGGCGTGCAGGGTTTCGTC
GAATTCATCAACAAGGGCAAGACCATCCTGCACCCCACGCCCTTCTACGCCGCGGGCGAG
AAGCCGGCCGAGACGTACGGCGGCATCCCCGGCACGCACATCGGCGTGGAAGTGTCGATG
CAGTGGAACAGCGGCTACACCGAGCAGGTGCTCTGTTTCACCAACAACATCCCGCAGCGC
GACGGCGGCACCCACCTGACCGGCCTGCGCGCCGCGATGACCCGGATCATCAACAAGTAC
ATCGAAGAAAACGAGCTGGCCAAGAAGGCCAAGGTCGAGGTAACCGGCGACGACATGCGC
GAAGAAGAGGACATGGCCAAGCGCCGTTCGCGCCTGAAGGCGTTGATCACGCTCGGCAAG
ACCCGCGGCTACCTGACGCAGGTCGAGATCTCCGACCACCTGCCCGACAAGCTGGTCGAC
GCCGAGACGATGGAAGTCGTGATCGCCATGTTGAACGACATGGGCATCGCGGTCTACGAG
CAGACGCCCGACGCCGAGACGCTGTTCCAGAACAACGTCACCCCCACCGCCGCCACTGTC
GAGGAAGCCGAGGAAGAAGCCGAGGCCGCGCTCTCCACCGTCGACAGCGAATTCGGCCGC
ACCACCGACCCGGTCCGCATGTACATGCGCGAAATGGGCACCGTCGAGCTGCTGACCCGC
GAAGGCGAGATCGAGATCGCCAAGCGCATCGAAGGCGGCATGCAGGCCATGATGGAGGCG
ATCAGCGCGTCGCCCGCCACCATCGCCGCGATCCTGGTGATGGGCGAGGACATCCGCGAA
GGCAAGGTCGTCATCTCGACCATCGTCGACGGCTTCTCCAACCCCAACGAAGCCGACGAC
TACGTGGCCGAGGAGGACTTCGACGAGTTCGACGAGGAAGACGACGACGACGGCAAGGGC
GGCTCCAAGGCGCTGACCAAGAAGCTGGAAGAATTGAAGCGCGAGGCGCTGTCGCGCTTC
GAC
>CFBP3681 Xylophilus ampelinus
CACGCCGGCGGCAAGTTCAACCAGAACAGCTACAAGGTCTCGGGCGGCCTGCACGGCGTG
GGCGTGAGTTGCGTCAACGCGCTCAGCAAGATGCTGCGCCTGACGGTGCGGCGCGACGGC
AAGGTCCACGTGCTGGAGTTCAGCAAGGGCTTCGTGCAGAACCGCATCGTCGAGCAGGCG
GGCGGCGTCGAGGTCTCGCCGATGCGCATCATGGGCGACACCGAGAAGCGCGGTACCGAG
GTGCACTTCCTGCCCGACAACGAGATCTTCAAGGAAAACGCCGATTTCCACTACGAGATC
CTGGCCAAGCGCCTGCGCGAACTGAGCTTCCTGAACAACGGCGTGCGCATCCGCCTGAAG
GACGAGCGCAGCGGCAAGGAAGACGATTTCTCCGGCGCCGGCGGCGTGCAGGGTTTCGTC
GAATTCATCAACAAGGGCAAGACCATCCTGCACCCCACGCCCTTCTACGCCGCGGGCGAG
AAGCCGGCCGAGACGTACGGCGGCATCCCCGGCACGCACATCGGCGTGGAAGTGTCGATG
CAGTGGAACAGCGGCTACACCGAGCAGGTGCTCTGTTTCACCAACAACATCCCGCAGCGC
GACGGCGGCACCCACCTGACCGGCCTGCGCGCCGCGATGACCCGGATCATCAACAAGTAC
ATCGAAGAAAACGAGCTGGCCAAGAAGGCCAAGGTCGAGGTAACCGGCGACGACATGCGC
GAAGAAGAGGACATGGCCAAGCGCCGTTCGCGCCTGAAGGCGTTGATCACGCTCGGCAAG
ACCCGCGGCTACCTGACGCAGGTCGAGATCTCCGACCACCTGCCCGACAAGCTGGTCGAC
GCCGAGACGATGGAAGTCGTGATCGCCATGTTGAACGACATGGGCATCGCGGTCTACGAG
CAGACGCCCGACGCCGAGACGCTGTTCCAGAACAACGTCACCCCCACCGCCGCCACTGTC
GAGGAAGCCGAGGAAGAAGCCGAGGCCGCGCTCTCCACCGTCGACAGCGAATTCGGCCGC
ACCACCGACCCGGTCCGCATGTACATGCGCGAAATGGGCACCGTCGAGCTGCTGACCCGC
GAAGGCGAGATCGAGATCGCCAAGCGCATCGAAGGCGGCCTGCAGGCCATGATGGAGGCG
ATCAGCGCGTCGCCCGCCACCATCGCCGCGATCCTGGTGATGGGCGAGGACATCCGCGAA
GGCAAGGTCGTCATCTCGACCATCGTCGACGGCTTCTCCAACCCCAACGAAGCCGACGAC
TACGTGGCCGAGGAGGACTTCGACGAGTTCGACGAGGAAGACGACGACGACGGCAAGGGC
GGCTCCAAGGCGCTGACCAAGAAGCTGGAAGAATTGAAGCGCGAGGCGCTGTCGCGCTTC
GAC
>CFBP3682 Xylophilus ampelinus
CACGCCGGCGGCAAGTTCAACCAGAACAGCTACAAGGTCTCGGGCGGCCTGCACGGCGTG
GGCGTGAGTTGCGTCAACGCGCTCAGCAAGATGCTGCGCCTGACGGTGCGGCGCGACGGC
AAGGTCCACGTGCTGGAGTTCAGCAAGGGCTTCGTGCAGAACCGCATCGTCGAGCAGGCG
GGCGGCGTCGAGGTCTCGCCGATGCGCATCATGGGCGACACCGAGAAGCGCGGTACCGAG
GTGCACTTCCTGCCCGACAACGAGATCTTCAAGGAAAACGCCGATTTCCACTACGAGATC
CTGGCCAAGCGCCTGCGCGAACTGAGCTTCCTGAACAACGGCGTGCGCATCCGCCTGAAG
GACGAGCGCAGCGGCAAGGAAGACGATTTCTCCGGCGCCGGCGGCGTGCAGGGTTTCGTC
GAATTCATCAACAAGGGCAAGACCATCCTGCACCCCACGCCCTTCTACGCCGCGGGCGAG

AAGCCGGCCGAGACGTACGGCGGCATCCCCGGCACGCACATCGGCGTGGAAGTGTCGATG
CAGTGGAACAGCGGCTACACCGAGCAGGTGCTCTGTTTCACCAACAACATCCCGCAGCGC
GACGGCGGCACCCACCTGACCGGCCTGCGCGCCGCGATGACCCGGATCATCAACAAGTAC
ATCGAAGAAAACGAGCTGGCCAAGAAGGCCAAGGTCGAGGTAACCGGCGACGACATGCGC
GAAGAAGAGGACATGGCCAAGCGCCGTTCGCGCCTGAAGGCGTTGATCACGCTCGGCAAG
ACCCGCGGCTACCTGACGCAGGTCGAGATCTCCGACCACCTGCCCGACAAGCTGGTCGAC
GCCGAGACGATGGAAGTCGTGATCGCCATGTTGAACGACATGGGCATCGCGGTCTACGAG
CAGACGCCCGACGCCGAGACGCTGTTCCAGAACAACGTCACCCCCACCGCCGCCACTGTC
GAGGAAGCCGAGGAAGAAGCCGAGGCCGCGCTCTCCACCGTCGACAGCGAATTCGGCCGC
ACCACCGACCCGGTCCGCATGTACATGCGCGAAATGGGCACCGTCGAGCTGCTGACCCGC
GAAGGCGAGATCGAGATCGCCAAGCGCATCGAAGGCGGCATGCAGGCCATGATGGAGGCG
ATCAGCGCGTCGCCCGCCACCATCGCCGCGATCCTGGTGATGGGCGAGGACATCCGCGAA
GGCAAGGTCGTCATCTCGACCATCGTCGACGGCTTCTCCAACCCCAACGAAGCCGACGAC
TACGTGGCCGAGGAGGACTTCGACGAGTTCGACGAGGAAGACGACGACGACGGCAAGGGC
GGCTCCAAGGCGCTGACCAAGAAGCTGGAAGAATTGAAGCGCGAGGCGCTGTCGCGCTTC
GAC
>CFBP3683 Xylophilus ampelinus
CACGCCGGCGGCAAGTTCAACCAGAACAGCTACAAGGTCTCGGGCGGCCTGCACGGCGTG
GGCGTGAGTTGCGTCAACGCGCTCAGCAAGATGCTGCGCCTGACGGTGCGGCGCGACGGC
AAGGTCCACGTGCTGGAGTTCAGCAAGGGCTTCGTGCAGAACCGCATCGTCGAGCAGGCG
GGCGGCGTCGAGGTCTCGCCGATGCGCATCATGGGCGACACCGAGAAGCGCGGTACCGAG
GTGCACTTCCTGCCCGACAACGAGATCTTCAAGGAAAACGCCGATTTCCACTACGAGATC
CTGGCCAAGCGCCTGCGCGAACTGAGCTTCCTGAACAACGGCGTGCGCATCCGCCTGAAG
GACGAGCGCAGCGGCAAGGAAGACGATTTCTCCGGCGCCGGCGGCGTGCAGGGTTTCGTC
GAATTCATCAACAAGGGCAAGACCATCCTGCACCCCACGCCCTTCTACGCCGCGGGCGAG
AAGCCGGCCGAGACGTACGGCGGCATCCCCGGCACGCACATCGGCGTGGAAGTGTCGATG
CAGTGGAACAGCGGCTACACCGAGCAGGTGCTCTGTTTCACCAACAACATCCCGCAGCGC
GACGGCGGCACCCACCTGACCGGCCTGCGCGCCGCGATGACCCGGATCATCAACAAGTAC
ATCGAAGAAAACGAGCTGGCCAAGAAGGCCAAGGTCGAGGTAACCGGCGACGACATGCGC
GAAGAAGAGGACATGGCCAAGCGCCGTTCGCGCCTGAAGGCGTTGATCACGCTCGGCAAG
ACCCGCGGCTACCTGACGCAGGTCGAGATCTCCGACCACCTGCCCGACAAGCTGGTCGAC
GCCGAGACGATGGAAGTCGTGATCGCCATGTTGAACGACATGGGCATCGCGGTCTACGAG
CAGACGCCCGACGCCGAGACGCTGTTCCAGAACAACGTCACCCCCACCGCCGCCACTGTC
GAGGAAGCCGAGGAAGAAGCCGAGGCCGCGCTCTCCACCGTCGACAGCGAATTCGGCCGC
ACCACCGACCCGGTCCGCATGTACATGCGCGAAATGGGCACCGTCGAGCTGCTGACCCGC
GAAGGCGAGATCGAGATCGCCAAGCGCATCGAAGGCGGCATGCAGGCCATGATGGAGGCG
ATCAGCGCGTCGCCCGCCACCATCGCCGCGATCCTGGTGATGGGCGAGGACATCCGCGAA
GGCAAGGTCGTCATCTCGACCATCGTCGACGGCTTCTCCAACCCCAACGAAGCCGACGAC
TACGTGGCCGAGGAGGACTTCGACGAGTTCGACGAGGAAGACGACGACGACGGCAAGGGC
GGCTCCAAGGCGCTGACCAAGAAGCTGGAAGAATTGAAGCGCGAGGCGCTGTCGCGCTTC
GAC
>CFBP3684 Xylophilus ampelinus
CACGCCGGCGGCAAGTTCAACCAGAACAGCTACAAGGTCTCGGGCGGCCTGCACGGCGTG
GGCGTGAGTTGCGTCAACGCGCTCAGCAAGATGCTGCGCCTGACGGTGCGGCGCGACGGC
AAGGTCCACGTGCTGGAGTTCAGCAAGGGCTTCGTGCAGAACCGCATCGTCGAGCAGGCG
GGCGGCGTCGAGGTCTCGCCGATGCGCATCATGGGCGACACCGAGAAGCGCGGTACCGAG
GTGCACTTCCTGCCCGACAACGAGATCTTCAAGGAAAACGCCGATTTCCACTACGAGATC
CTGGCCAAGCGCCTGCGCGAACTGAGCTTCCTGAACAACGGCGTGCGCATCCGCCTGAAG
GACGAGCGCAGCGGCAAGGAAGACGATTTCTCCGGCGCCGGCGGCGTGCAGGGTTTCGTC
GAATTCATCAACAAGGGCAAGACCATCCTGCACCCCACGCCCTTCTACGCCGCGGGCGAG
AAGCCGGCCGAGACGTACGGCGGCATCCCCGGCACGCACATCGGCGTGGAAGTGTCGATG
CAGTGGAACAGCGGCTACACCGAGCAGGTGCTCTGTTTCACCAACAACATCCCGCAGCGC
GACGGCGGCACCCACCTGACCGGCCTGCGCGCCGCGATGACCCGGATCATCAACAAGTAC
ATCGAAGAAAACGAGCTGGCCAAGAAGGCCAAGGTCGAGGTAACCGGCGACGACATGCGC
GAAGAAGAGGACATGGCCAAGCGCCGTTCGCGCCTGAAGGCGTTGATCACGCTCGGCAAG
ACCCGCGGCTACCTGACGCAGGTCGAGATCTCCGACCACCTGCCCGACAAGCTGGTCGAC
GCCGAGACGATGGAAGTCGTGATCGCCATGTTGAACGACATGGGCATCGCGGTCTACGAG
CAGACGCCCGACGCCGAGACGCTGTTCCAGAACAACGTCACCCCCACCGCCGCCACTGTC

GAGGAAGCCGAGGAAGAAGCCGAGGCCGCGCTCTCCACCGTCGACAGCGAATTCGGCCGC
ACCACCGACCCGGTCCGCATGTACATGCGCGAAATGGGCACCGTCGAGCTGCTGACCCGC
GAAGGCGAGATCGAGATCGCCAAGCGCATCGAAGGCGGCATGCAGGCCATGATGGAGGCG
ATCAGCGCGTCGCCCGCCACCATCGCCGCGATCCTGGTGATGGGCGAGGACATCCGCGAA
GGCAAGGTCGTCATCTCGACCATCGTCGACGGCTTCTCCAACCCCAACGAAGCCGACGAC
TACGTGGCCGAGGAGGACTTCGACGAGTTCGACGAGGAAGACGACGACGACGGCAAGGGC
GGCTCCAAGGCGCTGACCAAGAAGCTGGAAGAATTGAAGCGCGAGGCGCTGTCGCGCTTC
GAC
>CFBP3685 Xylophilus ampelinus
CACGCCGGCGGCAAGTTCAACCAGAACAGCTACAAGGTCTCGGGCGGCCTGCACGGCGTG
GGCGTGAGTTGCGTCAACGCGCTCAGCAAGATGCTGCGCCTGACGGTGCGGCGCGACGGC
AAGGTCCACGTGCTGGAGTTCAGCAAGGGCTTCGTGCAGAACCGCATCGTCGAGCAGGCG
GGCGGCGTCGAGGTCTCGCCGATGCGCATCATGGGCGACACCGAGAAGCGCGGTACCGAG
GTGCACTTCCTGCCCGACAACGAGATCTTCAAGGAAAACGCCGATTTCCACTACGAGATC
CTGGCCAAGCGCCTGCGCGAACTGAGCTTCCTGAACAACGGCGTGCGCATCCGCCTGAAG
GACGAGCGCAGCGGCAAGGAAGACGATTTCTCCGGCGCCGGCGGCGTGCAGGGTTTCGTC
GAATTCATCAACAAGGGCAAGACCATCCTGCACCCCACGCCCTTCTACGCCGCGGGCGAG
AAGCCGGCCGAGACGTACGGCGGCATCCCCGGCACGCACATCGGCGTGGAAGTGTCGATG
CAGTGGAACAGCGGCTACACCGAGCAGGTGCTCTGTTTCACCAACAACATCCCGCAGCGC
GACGGCGGCACCCACCTGACCGGCCTGCGCGCCGCGATGACCCGGATCATCAACAAGTAC
ATCGAAGAAAACGAGCTGGCCAAGAAGGCCAAGGTCGAGGTAACCGGCGACGACATGCGC
GAAGAAGAGGACATGGCCAAGCGCCGTTCGCGCCTGAAGGCGTTGATCACGCTCGGCAAG
ACCCGCGGCTACCTGACGCAGGTCGAGATCTCCGACCACCTGCCCGACAAGCTGGTCGAC
GCCGAGACGATGGAAGTCGTGATCGCCATGTTGAACGACATGGGCATCGCGGTCTACGAG
CAGACGCCCGACGCCGAGACGCTGTTCCAGAACAACGTCACCCCCACCGCCGCCACTGTC
GAGGAAGCCGAGGAAGAAGCCGAGGCCGCGCTCTCCACCGTCGACAGCGAATTCGGCCGC
ACCACCGACCCGGTCCGCATGTACATGCGCGAAATGGGCACCGTCGAGCTGCTGACCCGC
GAAGGCGAGATCGAGATCGCCAAGCGCATCGAAGGCGGCATGCAGGCCATGATGGAGGCG
ATCAGCGCGTCGCCCGCCACCATCGCCGCGATCCTGGTGATGGGCGAGGACATCCGCGAA
GGCAAGGTCGTCATCTCGACCATCGTCGACGGCTTCTCCAACCCCAACGAAGCCGACGAC
TACGTGGCCGAGGAGGACTTCGACGAGTTCGACGAGGAAGACGACGACGACGGCAAGGGC
GGCTCCAAGGCGCTGACCAAGAAGCTGGAAGAATTGAAGCGCGAGGCGCTGTCGCGCTTC
GAC
>CFBP3686 Xylophilus ampelinus
CACGCCGGCGGCAAGTTCAACCAGAACAGCTACAAGGTCTCGGGCGGCCTGCACGGCGTG
GGCGTGAGTTGCGTCAACGCGCTCAGCAAGATGCTGCGCCTGACGGTGCGGCGCGACGGC
AAGGTCCACGTGCTGGAGTTCAGCAAGGGCTTCGTGCAGAACCGCATCGTCGAGCAGGCG
GGCGGCGTCGAGGTCTCGCCGATGCGCATCATGGGCGACACCGAGAAGCGCGGTACCGAG
GTGCACTTCCTGCCCGACAACGAGATCTTCAAGGAAAACGCCGATTTCCACTACGAGATC
CTGGCCAAGCGCCTGCGCGAACTGAGCTTCCTGAACAACGGCGTGCGCATCCGCCTGAAG
GACGAGCGCAGCGGCAAGGAAGACGATTTCTCCGGCGCCGGCGGCGTGCAGGGTTTCGTC
GAATTCATCAACAAGGGCAAGACCATCCTGCACCCCACGCCCTTCTACGCCGCGGGCGAG
AAGCCGGCCGAGACGTACGGCGGCATCCCCGGCACGCACATCGGCGTGGAAGTGTCGATG
CAGTGGAACAGCGGCTACACCGAGCAGGTGCTCTGTTTCACCAACAACATCCCGCAGCGC
GACGGCGGCACCCACCTGACCGGCCTGCGCGCCGCGATGACCCGGATCATCAACAAGTAC
ATCGAAGAAAACGAGCTGGCCAAGAAGGCCAAGGTCGAGGTAACCGGCGACGACATGCGC
GAAGAAGAGGACATGGCCAAGCGCCGTTCGCGCCTGAAGGCGTTGATCACGCTCGGCAAG
ACCCGCGGCTACCTGACGCAGGTCGAGATCTCCGACCACCTGCCCGACAAGCTGGTCGAC
GCCGAGACGATGGAAGTCGTGATCGCCATGTTGAACGACATGGGCATCGCGGTCTACGAG
CAGACGCCCGACGCCGAGACGCTGTTCCAGAACAACGTCACCCCCACCGCCGCCACTGTC
GAGGAAGCCGAGGAAGAAGCCGAGGCCGCGCTCTCCACCGTCGACAGCGAATTCGGCCGC
ACCACCGACCCGGTCCGCATGTACATGCGCGAAATGGGCACCGTCGAGCTGCTGACCCGC
GAAGGCGAGATCGAGATCGCCAAGCGCATCGAAGGCGGCATGCAGGCCATGATGGAGGCG
ATCAGCGCGTCGCCCGCCACCATCGCCGCGATCCTGGTGATGGGCGAGGACATCCGCGAA
GGCAAGGTCGTCATCTCGACCATCGTCGACGGCTTCTCCAACCCCAACGAAGCCGACGAC
TACGTGGCCGAGGAGGACTTCGACGAGTTCGACGAGGAAGACGACGACGACGGCAAGGGC
GGCTCCAAGGCGCTGACCAAGAAGCTGGAAGAATTGAAGCGCGAGGCGCTGTCGCGCTTC
GAC

>CFBP3688 Xylophilus ampelinus
CACGCCGGCGGCAAGTTCAACCAGAACAGCTACAAGGTCTCGGGCGGCCTGCACGGCGTG
GGCGTGAGTTGCGTCAACGCGCTCAGCAAGATGCTGCGCCTGACGGTGCGGCGCGACGGC
AAGGTCCACGTGCTGGAGTTCAGCAAGGGCTTCGTGCAGAACCGCATCGTCGAGCAGGCG
GGCGGCGTCGAGGTCTCGCCGATGCGCATCATGGGCGACACCGAGAAGCGCGGTACCGAG
GTGCACTTCCTGCCCGACAACGAGATCTTCAAGGAAAACGCCGATTTCCACTACGAGATC
CTGGCCAAGCGCCTGCGCGAACTGAGCTTCCTGAACAACGGCGTGCGCATCCGCCTGAAG
GACGAGCGCAGCGGCAAGGAAGACGATTTCTCCGGCGCCGGCGGCGTGCAGGGTTTCGTC
GAATTCATCAACAAGGGCAAGACCATCCTGCACCCCACGCCCTTCTACGCCGCGGGCGAG
AAGCCGGCCGAGACGTACGGCGGCATCCCCGGCACGCACATCGGCGTGGAAGTGTCGATG
CAGTGGAACAGCGGCTACACCGAGCAGGTGCTCTGTTTCACCAACAACATCCCGCAGCGC
GACGGCGGCACCCACCTGACCGGCCTGCGCGCCGCGATGACCCGGATCATCAACAAGTAC
ATCGAAGAAAACGAGCTGGCCAAGAAGGCCAAGGTCGAGGTAACCGGCGACGACATGCGC
GAAGAAGAGGACATGGCCAAGCGCCGTTCGCGCCTGAAGGCGTTGATCACGCTCGGCAAG
ACCCGCGGCTACCTGACGCAGGTCGAGATCTCCGACCACCTGCCCGACAAGCTGGTCGAC
GCCGAGACGATGGAAGTCGTGATCGCCATGTTGAACGACATGGGCATCGCGGTCTACGAG
CAGACGCCCGACGCCGAGACGCTGTTCCAGAACAACGTCACCCCCACCGCCGCCACTGTC
GAGGAAGCCGAGGAAGAAGCCGAGGCCGCGCTCTCCACCGTCGACAGCGAATTCGGCCGC
ACCACCGACCCGGTCCGCATGTACATGCGCGAAATGGGCACCGTCGAGCTGCTGACCCGC
GAAGGCGAGATCGAGATCGCCAAGCGCATCGAAGGCGGCATGCAGGCCATGATGGAGGCG
ATCAGCGCGTCGCCCGCCACCATCGCCGCGATCCTGGTGATGGGCGAGGACATCCGCGAA
GGCAAGGTCGTCATCTCGACCATCGTCGACGGCTTCTCCAACCCCAACGAAGCCGACGAC
TACGTGGCCGAGGAGGACTTCGACGAGTTCGACGAGGAAGACGACGACGACGGCAAGGGC
GGCTCCAAGGCGCTGACCAAGAAGCTGGAAGAATTGAAGCGCGAGGCGCTGTCGCGCTTC
GAC
>CFBP3689 Xylophilus ampelinus
CACGCCGGCGGCAAGTTCAACCAGAACAGCTACAAGGTCTCGGGCGGCCTGCACGGCGTG
GGCGTGAGTTGCGTCAACGCGCTCAGCAAGATGCTGCGCCTGACGGTGCGGCGCGACGGC
AAGGTCCACGTGCTGGAGTTCAGCAAGGGCTTCGTGCAGAACCGCATCGTCGAGCAGGCG
GGCGGCGTCGAGGTCTCGCCGATGCGCATCATGGGCGACACCGAGAAGCGCGGTACCGAG
GTGCACTTCCTGCCCGACAACGAGATCTTCAAGGAAAACGCCGATTTCCACTACGAGATC
CTGGCCAAGCGCCTGCGCGAACTGAGCTTCCTGAACAACGGCGTGCGCATCCGCCTGAAG
GACGAGCGCAGCGGCAAGGAAGACGATTTCTCCGGCGCCGGCGGCGTGCAGGGTTTCGTC
GAATTCATCAACAAGGGCAAGACCATCCTGCACCCCACGCCCTTCTACGCCGCGGGCGAG
AAGCCGGCCGAGACGTACGGCGGCATCCCCGGCACGCACATCGGCGTGGAAGTGTCGATG
CAGTGGAACAGCGGCTACACCGAGCAGGTGCTCTGTTTCACCAACAACATCCCGCAGCGC
GACGGCGGCACCCACCTGACCGGCCTGCGCGCCGCGATGACCCGGATCATCAACAAGTAC
ATCGAAGAAAACGAGCTGGCCAAGAAGGCCAAGGTCGAGGTAACCGGCGACGACATGCGC
GAAGAAGAGGACATGGCCAAGCGCCGTTCGCGCCTGAAGGCGTTGATCACGCTCGGCAAG
ACCCGCGGCTACCTGACGCAGGTCGAGATCTCCGACCACCTGCCCGACAAGCTGGTCGAC
GCCGAGACGATGGAAGTCGTGATCGCCATGTTGAACGACATGGGCATCGCGGTCTACGAG
CAGACGCCCGACGCCGAGACGCTGTTCCAGAACAACGTCACCCCCACCGCCGCCACTGTC
GAGGAAGCCGAGGAAGAAGCCGAGGCCGCGCTCTCCACCGTCGACAGCGAATTCGGCCGC
ACCACCGACCCGGTCCGCATGTACATGCGCGAAATGGGCACCGTCGAGCTGCTGACCCGC
GAAGGCGAGATCGAGATCGCCAAGCGCATCGAAGGCGGCATGCAGGCCATGATGGAGGCG
ATCAGCGCGTCGCCCGCCACCATCGCCGCGATCCTGGTGATGGGCGAGGACATCCGCGAA
GGCAAGGTCGTCATCTCGACCATCGTCGACGGCTTCTCCAACCCCAACGAAGCCGACGAC
TACGTGGCCGAGGAGGACTTCGACGAGTTCGACGAGGAAGACGACGACGACGGCAAGGGC
GGCTCCAAGGCGCTGACCAAGAAGCTGGAAGAATTGAAGCGCGAGGCGCTGTCGCGCTTC
GAC
>CFBP3690 Xylophilus ampelinus
CACGCCGGCGGCAAGTTCAACCAGAACAGCTACAAGGTCTCGGGCGGCCTGCACGGCGTG
GGCGTGAGTTGCGTCAACGCGCTCAGCAAGATGCTGCGCCTGACGGTGCGGCGCGACGGC
AAGGTCCACGTGCTGGAGTTCAGCAAGGGCTTCGTGCAGAACCGCATCGTCGAGCAGGCG
GGCGGCGTCGAGGTCTCGCCGATGCGCATCATGGGCGACACCGAGAAGCGCGGTACCGAG
GTGCACTTCCTGCCCGACAACGAGATCTTCAAGGAAAACGCCGATTTCCACTACGAGATC
CTGGCCAAGCGCCTGCGCGAACTGAGCTTCCTGAACAACGGCGTGCGCATCCGCCTGAAG
GACGAGCGCAGCGGCAAGGAAGACGATTTCTCCGGCGCCGGCGGCGTGCAGGGTTTCGTC

GAATTCATCAACAAGGGCAAGACCATCCTGCACCCCACGCCCTTCTACGCCGCGGGCGAG
AAGCCGGCCGAGACGTACGGCGGCATCCCCGGCACGCACATCGGCGTGGAAGTGTCGATG
CAGTGGAACAGCGGCTACACCGAGCAGGTGCTCTGTTTCACCAACAACATCCCGCAGCGC
GACGGCGGCACCCACCTGACCGGCCTGCGCGCCGCGATGACCCGGATCATCAACAAGTAC
ATCGAAGAAAACGAGCTGGCCAAGAAGGCCAAGGTCGAGGTAACCGGCGACGACATGCGC
GAAGAAGAGGACATGGCCAAGCGCCGTTCGCGCCTGAAGGCGTTGATCACGCTCGGCAAG
ACCCGCGGCTACCTGACGCAGGTCGAGATCTCCGACCACCTGCCCGACAAGCTGGTCGAC
GCCGAGACGATGGAAGTCGTGATCGCCATGTTGAACGACATGGGCATCGCGGTCTACGAG
CAGACGCCCGACGCCGAGACGCTGTTCCAGAACAACGTCACCCCCACCGCCGCCACTGTC
GAGGAAGCCGAGGAAGAAGCCGAGGCCGCGCTCTCCACCGTCGACAGCGAATTCGGCCGC
ACCACCGACCCGGTCCGCATGTACATGCGCGAAATGGGCACCGTCGAGCTGCTGACCCGC
GAAGGCGAGATCGAGATCGCCAAGCGCATCGAAGGCGGCATGCAGGCCATGATGGAGGCG
ATCAGCGCGTCGCCCGCCACCATCGCCGCGATCCTGGTGATGGGCGAGGACATCCGCGAA
GGCAAGGTCGTCATCTCGACCATCGTCGACGGCTTCTCCAACCCCAACGAAGCCGACGAC
TACGTGGCCGAGGAGGACTTCGACGAGTTCGACGAGGAAGACGACGACGACGGCAAGGGC
GGCTCCAAGGCGCTGACCAAGAAGCTGGAAGAATTGAAGCGCGAGGCGCTGTCGCGCTTC
GAC
>CFBP3691 Xylophilus ampelinus
CACGCCGGCGGCAAGTTCAACCAGAACAGCTACAAGGTCTCGGGCGGCCTGCACGGCGTG
GGCGTGAGTTGCGTCAACGCGCTCAGCAAGATGCTGCGCCTGACGGTGCGGCGCGACGGC
AAGGTCCACGTGCTGGAGTTCAGCAAGGGCTTCGTGCAGAACCGCATCGTCGAGCAGGCG
GGCGGCGTCGAGGTCTCGCCGATGCGCATCATGGGCGACACCGAGAAGCGCGGTACCGAG
GTGCACTTCCTGCCCGACAACGAGATCTTCAAGGAAAACGCCGATTTCCACTACGAGATC
CTGGCCAAGCGCCTGCGCGAACTGAGCTTCCTGAACAACGGCGTGCGCATCCGCCTGAAG
GACGAGCGCAGCGGCAAGGAAGACGATTTCTCCGGCGCCGGCGGCGTGCAGGGTTTCGTC
GAATTCATCAACAAGGGCAAGACCATCCTGCACCCCACGCCCTTCTACGCCGCGGGCGAG
AAGCCGGCCGAGACGTACGGCGGCATCCCCGGCACGCACATCGGCGTGGAAGTGTCGATG
CAGTGGAACAGCGGCTACACCGAGCAGGTGCTCTGTTTCACCAACAACATCCCGCAGCGC
GACGGCGGCACCCACCTGACCGGCCTGCGCGCCGCGATGACCCGGATCATCAACAAGTAC
ATCGAAGAAAACGAGCTGGCCAAGAAGGCCAAGGTCGAGGTAACCGGCGACGACATGCGC
GAAGAAGAGGACATGGCCAAGCGCCGTTCGCGCCTGAAGGCGTTGATCACGCTCGGCAAG
ACCCGCGGCTACCTGACGCAGGTCGAGATCTCCGACCACCTGCCCGACAAGCTGGTCGAC
GCCGAGACGATGGAAGTCGTGATCGCCATGTTGAACGACATGGGCATCGCGGTCTACGAG
CAGACGCCCGACGCCGAGACGCTGTTCCAGAACAACGTCACCCCCACCGCCGCCACTGTC
GAGGAAGCCGAGGAAGAAGCCGAGGCCGCGCTCTCCACCGTCGACAGCGAATTCGGCCGC
ACCACCGACCCGGTCCGCATGTACATGCGCGAAATGGGCACCGTCGAGCTGCTGACCCGC
GAAGGCGAGATCGAGATCGCCAAGCGCATCGAAGGCGGCATGCAGGCCATGATGGAGGCG
ATCAGCGCGTCGCCCGCCACCATCGCCGCGATCCTGGTGATGGGCGAGGACATCCGCGAA
GGCAAGGTCGTCATCTCGACCATCGTCGACGGCTTCTCCAACCCCAACGAAGCCGACGAC
TACGTGGCCGAGGAGGACTTCGACGAGTTCGACGAGGAAGACGACGACGACGGCAAGGGC
GGCTCCAAGGCGCTGACCAAGAAGCTGGAAGAATTGAAGCGCGAGGCGCTGTCGCGCTTC
GAC
>CFBP3692 Xylophilus ampelinus
CACGCCGGCGGCAAGTTCAACCAGAACAGCTACAAGGTCTCGGGCGGCCTGCACGGCGTG
GGCGTGAGTTGCGTCAACGCGCTCAGCAAGATGCTGCGCCTGACGGTGCGGCGCGACGGC
AAGGTCCACGTGCTGGAGTTCAGCAAGGGCTTCGTGCAGAACCGCATCGTCGAGCAGGCG
GGCGGCGTCGAGGTCTCGCCGATGCGCATCATGGGCGACACCGAGAAGCGCGGTACCGAG
GTGCACTTCCTGCCCGACAACGAGATCTTCAAGGAAAACGCCGATTTCCACTACGAGATC
CTGGCCAAGCGCCTGCGCGAACTGAGCTTCCTGAACAACGGCGTGCGCATCCGCCTGAAG
GACGAGCGCAGCGGCAAGGAAGACGATTTCTCCGGCGCCGGCGGCGTGCAGGGTTTCGTC
GAATTCATCAACAAGGGCAAGACCATCCTGCACCCCACGCCCTTCTACGCCGCGGGCGAG
AAGCCGGCCGAGACGTACGGCGGCATCCCCGGCACGCACATCGGCGTGGAAGTGTCGATG
CAGTGGAACAGCGGCTACACCGAGCAGGTGCTCTGTTTCACCAACAACATCCCGCAGCGC
GACGGCGGCACCCACCTGACCGGCCTGCGCGCCGCGATGACCCGGATCATCAACAAGTAC
ATCGAAGAAAACGAGCTGGCCAAGAAGGCCAAGGTCGAGGTAACCGGCGACGACATGCGC
GAAGAAGAGGACATGGCCAAGCGCCGTTCGCGCCTGAAGGCGTTGATCACGCTCGGCAAG
ACCCGCGGCTACCTGACGCAGGTCGAGATCTCCGACCACCTGCCCGACAAGCTGGTCGAC
GCCGAGACGATGGAAGTCGTGATCGCCATGTTGAACGACATGGGCATCGCGGTCTACGAG

CAGACGCCCGACGCCGAGACGCTGTTCCAGAACAACGTCACCCCCACCGCCGCCACTGTC
GAGGAAGCCGAGGAAGAAGCCGAGGCCGCGCTCTCCACCGTCGACAGCGAATTCGGCCGC
ACCACCGACCCGGTCCGCATGTACATGCGCGAAATGGGCACCGTCGAGCTGCTGACCCGC
GAAGGCGAGATCGAGATCGCCAAGCGCATCGAAGGCGGCATGCAGGCCATGATGGAGGCG
ATCAGCGCGTCGCCCGCCACCATCGCCGCGATCCTGGTGATGGGCGAGGACATCCGCGAA
GGCAAGGTCGTCATCTCGACCATCGTCGACGGCTTCTCCAACCCCAACGAAGCCGACGAC
TACGTGGCCGAGGAGGACTTCGACGAGTTCGACGAGGAAGACGACGACGACGGCAAGGGC
GGCTCCAAGGCGCTGACCAAGAAGCTGGAAGAATTGAAGCGCGAGGCGCTGTCGCGCTTC
GAC
>CFBP4717 Xylophilus ampelinus
CACGCCGGCGGCAAGTTCAACCAGAACAGCTACAAGGTCTCGGGCGGCCTGCACGGCGTG
GGCGTGAGTTGCGTCAACGCGCTCAGCAAGATGCTGCGCCTGACGGTGCGGCGCGACGGC
AAGGTCCACGTGCTGGAGTTCAGCAAGGGCTTCGTGCAGAACCGCATCGTCGAGCAGGCG
GGCGGCGTCGAGGTCTCGCCGATGCGCATCATGGGCGACACCGAGAAGCGCGGTACCGAG
GTGCACTTCCTGCCCGACAACGAGATCTTCAAGGAAAACGCCGATTTCCACTACGAGATC
CTGGCCAAGCGCCTGCGCGAACTGAGCTTCCTGAACAACGGCGTGCGCATCCGCCTGAAG
GACGAGCGCAGCGGCAAGGAAGACGATTTCTCCGGCGCCGGCGGCGTGCAGGGTTTCGTC
GAATTCATCAACAAGGGCAAGACCATCCTGCACCCCACGCCCTTCTACGCCGCGGGCGAG
AAGCCGGCCGAGACGTACGGCGGCATCCCCGGCACGCACATCGGCGTGGAAGTGTCGATG
CAGTGGAACAGCGGCTACACCGAGCAGGTGCTCTGTTTCACCAACAACATCCCGCAGCGC
GACGGCGGCACCCACCTGACCGGCCTGCGCGCCGCGATGACCCGGATCATCAACAAGTAC
ATCGAAGAAAACGAGCTGGCCAAGAAGGCCAAGGTCGAGGTAACCGGCGACGACATGCGC
GAAGAAGAGGACATGGCCAAGCGCCGTTCGCGCCTGAAGGCGTTGATCACGCTCGGCAAG
ACCCGCGGCTACCTGACGCAGGTCGAGATCTCCGACCACCTGCCCGACAAGCTGGTCGAC
GCCGAGACGATGGAAGTCGTGATCGCCATGTTGAACGACATGGGCATCGCGGTCTACGAG
CAGACGCCCGACGCCGAGACGCTGTTCCAGAACAACGTCACCCCCACCGCCGCCACTGTC
GAGGAAGCCGAGGAAGAAGCCGAGGCCGCGCTCTCCACCGTCGACAGCGAATTCGGCCGC
ACCACCGACCCGGTCCGCATGTACATGCGCGAAATGGGCACCGTCGAGCTGCTGACCCGC
GAAGGCGAGATCGAGATCGCCAAGCGCATCGAAGGCGGCCTGCAGGCCATGATGGAGGCG
ATCAGCGCGTCGCCCGCCACCATCGCCGCGATCCTGGTGATGGGCGAGGACATCCGCGAA
GGCAAGGTCGTCATCTCGACCATCGTCGACGGCTTCTCCAACCCCAACGAAGCCGACGAC
TACGTGGCCGAGGAGGACTTCGACGAGTTCGACGAGGAAGACGACGACGACGGCAAGGGC
GGCTCCAAGGCGCTGACCAAGAAGCTGGAAGAATTGAAGCGCGAGGCGCTGTCGCGCTTC
GAC
>CFBP4718 Xylophilus ampelinus
CACGCCGGCGGCAAGTTCAACCAGAACAGCTACAAGGTCTCGGGCGGCCTGCACGGCGTG
GGCGTGAGTTGCGTCAACGCGCTCAGCAAGATGCTGCGCCTGACGGTGCGGCGCGACGGC
AAGGTCCACGTGCTGGAGTTCAGCAAGGGCTTCGTGCAGAACCGCATCGTCGAGCAGGCG
GGCGGCGTCGAGGTCTCGCCGATGCGCATCATGGGCGACACCGAGAAGCGCGGTACCGAG
GTGCACTTCCTGCCCGACAACGAGATCTTCAAGGAAAACGCCGATTTCCACTACGAGATC
CTGGCCAAGCGCCTGCGCGAACTGAGCTTCCTGAACAACGGCGTGCGCATCCGCCTGAAG
GACGAGCGCAGCGGCAAGGAAGACGATTTCTCCGGCGCCGGCGGCGTGCAGGGTTTCGTC
GAATTCATCAACAAGGGCAAGACCATCCTGCACCCCACGCCCTTCTACGCCGCGGGCGAG
AAGCCGGCCGAGACGTACGGCGGCATCCCCGGCACGCACATCGGCGTGGAAGTGTCGATG
CAGTGGAACAGCGGCTACACCGAGCAGGTGCTCTGTTTCACCAACAACATCCCGCAGCGC
GACGGCGGCACCCACCTGACCGGCCTGCGCGCCGCGATGACCCGGATCATCAACAAGTAC
ATCGAAGAAAACGAGCTGGCCAAGAAGGCCAAGGTCGAGGTAACCGGCGACGACATGCGC
GAAGAAGAGGACATGGCCAAGCGCCGTTCGCGCCTGAAGGCGTTGATCACGCTCGGCAAG
ACCCGCGGCTACCTGACGCAGGTCGAGATCTCCGACCACCTGCCCGACAAGCTGGTCGAC
GCCGAGACGATGGAAGTCGTGATCGCCATGTTGAACGACATGGGCATCGCGGTCTACGAG
CAGACGCCCGACGCCGAGACGCTGTTCCAGAACAACGTCACCCCCACCGCCGCCACTGTC
GAGGAAGCCGAGGAAGAAGCCGAGGCCGCGCTCTCCACCGTCGACAGCGAATTCGGCCGC
ACCACCGACCCGGTCCGCATGTACATGCGCGAAATGGGCACCGTCGAGCTGCTGACCCGC
GAAGGCGAGATCGAGATCGCCAAGCGCATCGAAGGCGGCCTGCAGGCCATGATGGAGGCG
ATCAGCGCGTCGCCCGCCACCATCGCCGCGATCCTGGTGATGGGCGAGGACATCCGCGAA
GGCAAGGTCGTCATCTCGACCATCGTCGACGGCTTCTCCAACCCCAACGAAGCCGACGAC
TACGTGGCCGAGGAGGACTTCGACGAGTTCGACGAGGAAGACGACGACGACGGCAAGGGC
GGCTCCAAGGCGCTGACCAAGAAGCTGGAAGAATTGAAGCGCGAGGCGCTGTCGCGCTTC

GAC
>CFBP4864 Xylophilus ampelinus
CACGCCGGCGGCAAGTTCAACCAGAACAGCTACAAGGTCTCGGGCGGCCTGCACGGCGTG
GGCGTGAGTTGCGTCAACGCGCTCAGCAAGATGCTGCGCCTGACGGTGCGGCGCGACGGC
AAGGTCCACGTGCTGGAGTTCAGCAAGGGCTTCGTGCAGAACCGCATCGTCGAGCAGGCG
GGCGGCGTCGAGGTCTCGCCGATGCGCATCATGGGCGACACCGAGAAGCGCGGTACCGAG
GTGCACTTCCTGCCCGACAACGAGATCTTCAAGGAAAACGCCGATTTCCACTACGAGATC
CTGGCCAAGCGCCTGCGCGAACTGAGCTTCCTGAACAACGGCGTGCGCATCCGCCTGAAG
GACGAGCGCAGCGGCAAGGAAGACGATTTCTCCGGCGCCGGCGGCGTGCAGGGTTTCGTC
GAATTCATCAACAAGGGCAAGACCATCCTGCACCCCACGCCCTTCTACGCCGCGGGCGAG
AAGCCGGCCGAGACGTACGGCGGCATCCCCGGCACGCACATCGGCGTGGAAGTGTCGATG
CAGTGGAACAGCGGCTACACCGAGCAGGTGCTCTGTTTCACCAACAACATCCCGCAGCGC
GACGGCGGCACCCACCTGACCGGCCTGCGCGCCGCGATGACCCGGATCATCAACAAGTAC
ATCGAAGAAAACGAGCTGGCCAAGAAGGCCAAGGTCGAGGTAACCGGCGACGACATGCGC
GAAGAAGAGGACATGGCCAAGCGCCGTTCGCGCCTGAAGGCGTTGATCACGCTCGGCAAG
ACCCGCGGCTACCTGACGCAGGTCGAGATCTCCGACCACCTGCCCGACAAGCTGGTCGAC
GCCGAGACGATGGAAGTCGTGATCGCCATGTTGAACGACATGGGCATCGCGGTCTACGAG
CAGACGCCCGACGCCGAGACGCTGTTCCAGAACAACGTCACCCCCACCGCCGCCACTGTC
GAGGAAGCCGAGGAAGAAGCCGAGGCCGCGCTCTCCACCGTCGACAGCGAATTCGGCCGC
ACCACCGACCCGGTCCGCATGTACATGCGCGAAATGGGCACCGTCGAGCTGCTGACCCGC
GAAGGCGAGATCGAGATCGCCAAGCGCATCGAAGGCGGCCTGCAGGCCATGATGGAGGCG
ATCAGCGCGTCGCCCGCCACCATCGCCGCGATCCTGGTGATGGGCGAGGACATCCGCGAA
GGCAAGGTCGTCATCTCGACCATCGTCGACGGCTTCTCCAACCCCAACGAAGCCGACGAC
TACGTGGCCGAGGAGGACTTCGACGAGTTCGACGAGGAAGACGACGACGACGGCAAGGGC
GGCTCCAAGGCGCTGACCAAGAAGCTGGAAGAATTGAAGCGCGAGGCGCTGTCGCGCTTC
GAC
>CFBP4866 Xylophilus ampelinus
CACGCCGGCGGCAAGTTCAACCAGAACAGCTACAAGGTCTCGGGCGGCCTGCACGGCGTG
GGCGTGAGTTGCGTCAACGCGCTCAGCAAGATGCTGCGCCTGACGGTGCGGCGCGACGGC
AAGGTCCACGTGCTGGAGTTCAGCAAGGGCTTCGTGCAGAACCGCATCGTCGAGCAGGCG
GGCGGCGTCGAGGTCTCGCCGATGCGCATCATGGGCGACACCGAGAAGCGCGGTACCGAG
GTGCACTTCCTGCCCGACAACGAGATCTTCAAGGAAAACGCCGATTTCCACTACGAGATC
CTGGCCAAGCGCCTGCGCGAACTGAGCTTCCTGAACAACGGCGTGCGCATCCGCCTGAAG
GACGAGCGCAGCGGCAAGGAAGACGATTTCTCCGGCGCCGGCGGCGTGCAGGGTTTCGTC
GAATTCATCAACAAGGGCAAGACCATCCTGCACCCCACGCCCTTCTACGCCGCGGGCGAG
AAGCCGGCCGAGACGTACGGCGGCATCCCCGGCACGCACATCGGCGTGGAAGTGTCGATG
CAGTGGAACAGCGGCTACACCGAGCAGGTGCTCTGTTTCACCAACAACATCCCGCAGCGC
GACGGCGGCACCCACCTGACCGGCCTGCGCGCCGCGATGACCCGGATCATCAACAAGTAC
ATCGAAGAAAACGAGCTGGCCAAGAAGGCCAAGGTCGAGGTAACCGGCGACGACATGCGC
GAAGAAGAGGACATGGCCAAGCGCCGTTCGCGCCTGAAGGCGTTGATCACGCTCGGCAAG
ACCCGCGGCTACCTGACGCAGGTCGAGATCTCCGACCACCTGCCCGACAAGCTGGTCGAC
GCCGAGACGATGGAAGTCGTGATCGCCATGTTGAACGACATGGGCATCGCGGTCTACGAG
CAGACGCCCGACGCCGAGACGCTGTTCCAGAACAACGTCACCCCCACCGCCGCCACTGTC
GAGGAAGCCGAGGAAGAAGCCGAGGCCGCGCTCTCCACCGTCGACAGCGAATTCGGCCGC
ACCACCGACCCGGTCCGCATGTACATGCGCGAAATGGGCACCGTCGAGCTGCTGACCCGC
GAAGGCGAGATCGAGATCGCCAAGCGCATCGAAGGCGGCCTGCAGGCCATGATGGAGGCG
ATCAGCGCGTCGCCCGCCACCATCGCCGCGATCCTGGTGATGGGCGAGGACATCCGCGAA
GGCAAGGTCGTCATCTCGACCATCGTCGACGGCTTCTCCAACCCCAACGAAGCCGACGAC
TACGTGGCCGAGGAGGACTTCGACGAGTTCGACGAGGAAGACGACGACGACGGCAAGGGC
GGCTCCAAGGCGCTGACCAAGAAGCTGGAAGAATTGAAGCGCGAGGCGCTGTCGCGCTTC
GAC
>CFBP4867 Xylophilus ampelinus
CACGCCGGCGGCAAGTTCAACCAGAACAGCTACAAGGTCTCGGGCGGCCTGCACGGCGTG
GGCGTGAGTTGCGTCAACGCGCTCAGCAAGATGCTGCGCCTGACGGTGCGGCGCGACGGC
AAGGTCCACGTGCTGGAGTTCAGCAAGGGCTTCGTGCAGAACCGCATCGTCGAGCAGGCG
GGCGGCGTCGAGGTCTCGCCGATGCGCATCATGGGCGACACCGAGAAGCGCGGTACCGAG
GTGCACTTCCTGCCCGACAACGAGATCTTCAAGGAAAACGCCGATTTCCACTACGAGATC
CTGGCCAAGCGCCTGCGCGAACTGAGCTTCCTGAACAACGGCGTGCGCATCCGCCTGAAG

GACGAGCGCAGCGGCAAGGAAGACGATTTCTCCGGCGCCGGCGGCGTGCAGGGTTTCGTC
GAATTCATCAACAAGGGCAAGACCATCCTGCACCCCACGCCCTTCTACGCCGCGGGCGAG
AAGCCGGCCGAGACGTACGGCGGCATCCCCGGCACGCACATCGGCGTGGAAGTGTCGATG
CAGTGGAACAGCGGCTACACCGAGCAGGTGCTCTGTTTCACCAACAACATCCCGCAGCGC
GACGGCGGCACCCACCTGACCGGCCTGCGCGCCGCGATGACCCGGATCATCAACAAGTAC
ATCGAAGAAAACGAGCTGGCCAAGAAGGCCAAGGTCGAGGTAACCGGCGACGACATGCGC
GAAGAAGAGGACATGGCCAAGCGCCGTTCGCGCCTGAAGGCGTTGATCACGCTCGGCAAG
ACCCGCGGCTACCTGACGCAGGTCGAGATCTCCGACCACCTGCCCGACAAGCTGGTCGAC
GCCGAGACGATGGAAGTCGTGATCGCCATGTTGAACGACATGGGCATCGCGGTCTACGAG
CAGACGCCCGACGCCGAGACGCTGTTCCAGAACAACGTCACCCCCACCGCCGCCACTGTC
GAGGAAGCCGAGGAAGAAGCCGAGGCCGCGCTCTCCACCGTCGACAGCGAATTCGGCCGC
ACCACCGACCCGGTCCGCATGTACATGCGCGAAATGGGCACCGTCGAGCTGCTGACCCGC
GAAGGCGAGATCGAGATCGCCAAGCGCATCGAAGGCGGCCTGCAGGCCATGATGGAGGCG
ATCAGCGCGTCGCCCGCCACCATCGCCGCGATCCTGGTGATGGGCGAGGACATCCGCGAA
GGCAAGGTCGTCATCTCGACCATCGTCGACGGCTTCTCCAACCCCAACGAAGCCGACGAC
TACGTGGCCGAGGAGGACTTCGACGAGTTCGACGAGGAAGACGACGACGACGGCAAGGGC
GGCTCCAAGGCGCTGACCAAGAAGCTGGAAGAATTGAAGCGCGAGGCGCTGTCGCGCTTC
GAC
>CFBP4868 Xylophilus ampelinus
CACGCCGGCGGCAAGTTCAACCAGAACAGCTACAAGGTCTCGGGCGGCCTGCACGGCGTG
GGCGTGAGTTGCGTCAACGCGCTCAGCAAGATGCTGCGCCTGACGGTGCGGCGCGACGGC
AAGGTCCACGTGCTGGAGTTCAGCAAGGGCTTCGTGCAGAACCGCATCGTCGAGCAGGCG
GGCGGCGTCGAGGTCTCGCCGATGCGCATCATGGGCGACACCGAGAAGCGCGGTACCGAG
GTGCACTTCCTGCCCGACAACGAGATCTTCAAGGAAAACGCCGATTTCCACTACGAGATC
CTGGCCAAGCGCCTGCGCGAACTGAGCTTCCTGAACAACGGCGTGCGCATCCGCCTGAAG
GACGAGCGCAGCGGCAAGGAAGACGATTTCTCCGGCGCCGGCGGCGTGCAGGGTTTCGTC
GAATTCATCAACAAGGGCAAGACCATCCTGCACCCCACGCCCTTCTACGCCGCGGGCGAG
AAGCCGGCCGAGACGTACGGCGGCATCCCCGGCACGCACATCGGCGTGGAAGTGTCGATG
CAGTGGAACAGCGGCTACACCGAGCAGGTGCTCTGTTTCACCAACAACATCCCGCAGCGC
GACGGCGGCACCCACCTGACCGGCCTGCGCGCCGCGATGACCCGGATCATCAACAAGTAC
ATCGAAGAAAACGAGCTGGCCAAGAAGGCCAAGGTCGAGGTAACCGGCGACGACATGCGC
GAAGAAGAGGACATGGCCAAGCGCCGTTCGCGCCTGAAGGCGTTGATCACGCTCGGCAAG
ACCCGCGGCTACCTGACGCAGGTCGAGATCTCCGACCACCTGCCCGACAAGCTGGTCGAC
GCCGAGACGATGGAAGTCGTGATCGCCATGTTGAACGACATGGGCATCGCGGTCTACGAG
CAGACGCCCGACGCCGAGACGCTGTTCCAGAACAACGTCACCCCCACCGCCGCCACTGTC
GAGGAAGCCGAGGAAGAAGCCGAGGCCGCGCTCTCCACCGTCGACAGCGAATTCGGCCGC
ACCACCGACCCGGTCCGCATGTACATGCGCGAAATGGGCACCGTCGAGCTGCTGACCCGC
GAAGGCGAGATCGAGATCGCCAAGCGCATCGAAGGCGGCCTGCAGGCCATGATGGAGGCG
ATCAGCGCGTCGCCCGCCACCATCGCCGCGATCCTGGTGATGGGCGAGGACATCCGCGAA
GGCAAGGTCGTCATCTCGACCATCGTCGACGGCTTCTCCAACCCCAACGAAGCCGACGAC
TACGTGGCCGAGGAGGACTTCGACGAGTTCGACGAGGAAGACGACGACGACGGCAAGGGC
GGCTCCAAGGCGCTGACCAAGAAGCTGGAAGAATTGAAGCGCGAGGCGCTGTCGCGCTTC
GAC
>CFBP4869 Xylophilus ampelinus
CACGCCGGCGGCAAGTTCAACCAGAACAGCTACAAGGTCTCGGGCGGCCTGCACGGCGTG
GGCGTGAGTTGCGTCAACGCGCTCAGCAAGATGCTGCGCCTGACGGTGCGGCGCGACGGC
AAGGTCCACGTGCTGGAGTTCAGCAAGGGCTTCGTGCAGAACCGCATCGTCGAGCAGGCG
GGCGGCGTCGAGGTCTCGCCGATGCGCATCATGGGCGACACCGAGAAGCGCGGTACCGAG
GTGCACTTCCTGCCCGACAACGAGATCTTCAAGGAAAACGCCGATTTCCACTACGAGATC
CTGGCCAAGCGCCTGCGCGAACTGAGCTTCCTGAACAACGGCGTGCGCATCCGCCTGAAG
GACGAGCGCAGCGGCAAGGAAGACGATTTCTCCGGCGCCGGCGGCGTGCAGGGTTTCGTC
GAATTCATCAACAAGGGCAAGACCATCCTGCACCCCACGCCCTTCTACGCCGCGGGCGAG
AAGCCGGCCGAGACGTACGGCGGCATCCCCGGCACGCACATCGGCGTGGAAGTGTCGATG
CAGTGGAACAGCGGCTACACCGAGCAGGTGCTCTGTTTCACCAACAACATCCCGCAGCGC
GACGGCGGCACCCACCTGACCGGCCTGCGCGCCGCGATGACCCGGATCATCAACAAGTAC
ATCGAAGAAAACGAGCTGGCCAAGAAGGCCAAGGTCGAGGTAACCGGCGACGACATGCGC
GAAGAAGAGGACATGGCCAAGCGCCGTTCGCGCCTGAAGGCGTTGATCACGCTCGGCAAG
ACCCGCGGCTACCTGACGCAGGTCGAGATCTCCGACCACCTGCCCGACAAGCTGGTCGAC

GCCGAGACGATGGAAGTCGTGATCGCCATGTTGAACGACATGGGCATCGCGGTCTACGAG
CAGACGCCCGACGCCGAGACGCTGTTCCAGAACAACGTCACCCCCACCGCCGCCACTGTC
GAGGAAGCCGAGGAAGAAGCCGAGGCCGCGCTCTCCACCGTCGACAGCGAATTCGGCCGC
ACCACCGACCCGGTCCGCATGTACATGCGCGAAATGGGCACCGTCGAGCTGCTGACCCGC
GAAGGCGAGATCGAGATCGCCAAGCGCATCGAAGGCGGCCTGCAGGCCATGATGGAGGCG
ATCAGCGCGTCGCCCGCCACCATCGCCGCGATCCTGGTGATGGGCGAGGACATCCGCGAA
GGCAAGGTCGTCATCTCGACCATCGTCGACGGCTTCTCCAACCCCAACGAAGCCGACGAC
TACGTGGCCGAGGAGGACTTCGACGAGTTCGACGAGGAAGACGACGACGACGGCAAGGGC
GGCTCCAAGGCGCTGACCAAGAAGCTGGAAGAATTGAAGCGCGAGGCGCTGTCGCGCTTC
GAC
>CFBP4870 Xylophilus ampelinus
CACGCCGGCGGCAAGTTCAACCAGAACAGCTACAAGGTCTCGGGCGGCCTGCACGGCGTG
GGCGTGAGTTGCGTCAACGCGCTCAGCAAGATGCTGCGCCTGACGGTGCGGCGCGACGGC
AAGGTCCACGTGCTGGAGTTCAGCAAGGGCTTCGTGCAGAACCGCATCGTCGAGCAGGCG
GGCGGCGTCGAGGTCTCGCCGATGCGCATCATGGGCGACACCGAGAAGCGCGGTACCGAG
GTGCACTTCCTGCCCGACAACGAGATCTTCAAGGAAAACGCCGATTTCCACTACGAGATC
CTGGCCAAGCGCCTGCGCGAACTGAGCTTCCTGAACAACGGCGTGCGCATCCGCCTGAAG
GACGAGCGCAGCGGCAAGGAAGACGATTTCTCCGGCGCCGGCGGCGTGCAGGGTTTCGTC
GAATTCATCAACAAGGGCAAGACCATCCTGCACCCCACGCCCTTCTACGCCGCGGGCGAG
AAGCCGGCCGAGACGTACGGCGGCATCCCCGGCACGCACATCGGCGTGGAAGTGTCGATG
CAGTGGAACAGCGGCTACACCGAGCAGGTGCTCTGTTTCACCAACAACATCCCGCAGCGC
GACGGCGGCACCCACCTGACCGGCCTGCGCGCCGCGATGACCCGGATCATCAACAAGTAC
ATCGAAGAAAACGAGCTGGCCAAGAAGGCCAAGGTCGAGGTAACCGGCGACGACATGCGC
GAAGAAGAGGACATGGCCAAGCGCCGTTCGCGCCTGAAGGCGTTGATCACGCTCGGCAAG
ACCCGCGGCTACCTGACGCAGGTCGAGATCTCCGACCACCTGCCCGACAAGCTGGTCGAC
GCCGAGACGATGGAAGTCGTGATCGCCATGTTGAACGACATGGGCATCGCGGTCTACGAG
CAGACGCCCGACGCCGAGACGCTGTTCCAGAACAACGTCACCCCCACCGCCGCCACTGTC
GAGGAAGCCGAGGAAGAAGCCGAGGCCGCGCTCTCCACCGTCGACAGCGAATTCGGCCGC
ACCACCGACCCGGTCCGCATGTACATGCGCGAAATGGGCACCGTCGAGCTGCTGACCCGC
GAAGGCGAGATCGAGATCGCCAAGCGCATCGAAGGCGGCCTGCAGGCCATGATGGAGGCG
ATCAGCGCGTCGCCCGCCACCATCGCCGCGATCCTGGTGATGGGCGAGGACATCCGCGAA
GGCAAGGTCGTCATCTCGACCATCGTCGACGGCTTCTCCAACCCCAACGAAGCCGACGAC
TACGTGGCCGAGGAGGACTTCGACGAGTTCGACGAGGAAGACGACGACGACGGCAAGGGC
GGCTCCAAGGCGCTGACCAAGAAGCTGGAAGAATTGAAGCGCGAGGCGCTGTCGCGCTTC
GAC
>CFBP4871 Xylophilus ampelinus
CACGCCGGCGGCAAGTTCAACCAGAACAGCTACAAGGTCTCGGGCGGCCTGCACGGCGTG
GGCGTGAGTTGCGTCAACGCGCTCAGCAAGATGCTGCGCCTGACGGTGCGGCGCGACGGC
AAGGTCCACGTGCTGGAGTTCAGCAAGGGCTTCGTGCAGAACCGCATCGTCGAGCAGGCG
GGCGGCGTCGAGGTCTCGCCGATGCGCATCATGGGCGACACCGAGAAGCGCGGTACCGAG
GTGCACTTCCTGCCCGACAACGAGATCTTCAAGGAAAACGCCGATTTCCACTACGAGATC
CTGGCCAAGCGCCTGCGCGAACTGAGCTTCCTGAACAACGGCGTGCGCATCCGCCTGAAG
GACGAGCGCAGCGGCAAGGAAGACGATTTCTCCGGCGCCGGCGGCGTGCAGGGTTTCGTC
GAATTCATCAACAAGGGCAAGACCATCCTGCACCCCACGCCCTTCTACGCCGCGGGCGAG
AAGCCGGCCGAGACGTACGGCGGCATCCCCGGCACGCACATCGGCGTGGAAGTGTCGATG
CAGTGGAACAGCGGCTACACCGAGCAGGTGCTCTGTTTCACCAACAACATCCCGCAGCGC
GACGGCGGCACCCACCTGACCGGCCTGCGCGCCGCGATGACCCGGATCATCAACAAGTAC
ATCGAAGAAAACGAGCTGGCCAAGAAGGCCAAGGTCGAGGTAACCGGCGACGACATGCGC
GAAGAAGAGGACATGGCCAAGCGCCGTTCGCGCCTGAAGGCGTTGATCACGCTCGGCAAG
ACCCGCGGCTACCTGACGCAGGTCGAGATCTCCGACCACCTGCCCGACAAGCTGGTCGAC
GCCGAGACGATGGAAGTCGTGATCGCCATGTTGAACGACATGGGCATCGCGGTCTACGAG
CAGACGCCCGACGCCGAGACGCTGTTCCAGAACAACGTCACCCCCACCGCCGCCACTGTC
GAGGAAGCCGAGGAAGAAGCCGAGGCCGCGCTCTCCACCGTCGACAGCGAATTCGGCCGC
ACCACCGACCCGGTCCGCATGTACATGCGCGAAATGGGCACCGTCGAGCTGCTGACCCGC
GAAGGCGAGATCGAGATCGCCAAGCGCATCGAAGGCGGCCTGCAGGCCATGATGGAGGCG
ATCAGCGCGTCGCCCGCCACCATCGCCGCGATCCTGGTGATGGGCGAGGACATCCGCGAA
GGCAAGGTCGTCATCTCGACCATCGTCGACGGCTTCTCCAACCCCAACGAAGCCGACGAC
TACGTGGCCGAGGAGGACTTCGACGAGTTCGACGAGGAAGACGACGACGACGGCAAGGGC

GGCTCCAAGGCGCTGACCAAGAAGCTGGAAGAATTGAAGCGCGAGGCGCTGTCGCGCTTC
GAC
>CFBP4872 Xylophilus ampelinus
CACGCCGGCGGCAAGTTCAACCAGAACAGCTACAAGGTCTCGGGCGGCCTGCACGGCGTG
GGCGTGAGTTGCGTCAACGCGCTCAGCAAGATGCTGCGCCTGACGGTGCGGCGCGACGGC
AAGGTCCACGTGCTGGAGTTCAGCAAGGGCTTCGTGCAGAACCGCATCGTCGAGCAGGCG
GGCGGCGTCGAGGTCTCGCCGATGCGCATCATGGGCGACACCGAGAAGCGCGGTACCGAG
GTGCACTTCCTGCCCGACAACGAGATCTTCAAGGAAAACGCCGATTTCCACTACGAGATC
CTGGCCAAGCGCCTGCGCGAACTGAGCTTCCTGAACAACGGCGTGCGCATCCGCCTGAAG
GACGAGCGCAGCGGCAAGGAAGACGATTTCTCCGGCGCCGGCGGCGTGCAGGGTTTCGTC
GAATTCATCAACAAGGGCAAGACCATCCTGCACCCCACGCCCTTCTACGCCGCGGGCGAG
AAGCCGGCCGAGACGTACGGCGGCATCCCCGGCACGCACATCGGCGTGGAAGTGTCGATG
CAGTGGAACAGCGGCTACACCGAGCAGGTGCTCTGTTTCACCAACAACATCCCGCAGCGC
GACGGCGGCACCCACCTGACCGGCCTGCGCGCCGCGATGACCCGGATCATCAACAAGTAC
ATCGAAGAAAACGAGCTGGCCAAGAAGGCCAAGGTCGAGGTAACCGGCGACGACATGCGC
GAAGAAGAGGACATGGCCAAGCGCCGTTCGCGCCTGAAGGCGTTGATCACGCTCGGCAAG
ACCCGCGGCTACCTGACGCAGGTCGAGATCTCCGACCACCTGCCCGACAAGCTGGTCGAC
GCCGAGACGATGGAAGTCGTGATCGCCATGTTGAACGACATGGGCATCGCGGTCTACGAG
CAGACGCCCGACGCCGAGACGCTGTTCCAGAACAACGTCACCCCCACCGCCGCCACTGTC
GAGGAAGCCGAGGAAGAAGCCGAGGCCGCGCTCTCCACCGTCGACAGCGAATTCGGCCGC
ACCACCGACCCGGTCCGCATGTACATGCGCGAAATGGGCACCGTCGAGCTGCTGACCCGC
GAAGGCGAGATCGAGATCGCCAAGCGCATCGAAGGCGGCCTGCAGGCCATGATGGAGGCG
ATCAGCGCGTCGCCCGCCACCATCGCCGCGATCCTGGTGATGGGCGAGGACATCCGCGAA
GGCAAGGTCGTCATCTCGACCATCGTCGACGGCTTCTCCAACCCCAACGAAGCCGACGAC
TACGTGGCCGAGGAGGACTTCGACGAGTTCGACGAGGAAGACGACGACGACGGCAAGGGC
GGCTCCAAGGCGCTGACCAAGAAGCTGGAAGAATTGAAGCGCGAGGCGCTGTCGCGCTTC
GAC
>CFBP4873 Xylophilus ampelinus
CACGCCGGCGGCAAGTTCAACCAGAACAGCTACAAGGTCTCGGGCGGCCTGCACGGCGTG
GGCGTGAGTTGCGTCAACGCGCTCAGCAAGATGCTGCGCCTGACGGTGCGGCGCGACGGC
AAGGTCCACGTGCTGGAGTTCAGCAAGGGCTTCGTGCAGAACCGCATCGTCGAGCAGGCG
GGCGGCGTCGAGGTCTCGCCGATGCGCATCATGGGCGACACCGAGAAGCGCGGTACCGAG
GTGCACTTCCTGCCCGACAACGAGATCTTCAAGGAAAACGCCGATTTCCACTACGAGATC
CTGGCCAAGCGCCTGCGCGAACTGAGCTTCCTGAACAACGGCGTGCGCATCCGCCTGAAG
GACGAGCGCAGCGGCAAGGAAGACGATTTCTCCGGCGCCGGCGGCGTGCAGGGTTTCGTC
GAATTCATCAACAAGGGCAAGACCATCCTGCACCCCACGCCCTTCTACGCCGCGGGCGAG
AAGCCGGCCGAGACGTACGGCGGCATCCCCGGCACGCACATCGGCGTGGAAGTGTCGATG
CAGTGGAACAGCGGCTACACCGAGCAGGTGCTCTGTTTCACCAACAACATCCCGCAGCGC
GACGGCGGCACCCACCTGACCGGCCTGCGCGCCGCGATGACCCGGATCATCAACAAGTAC
ATCGAAGAAAACGAGCTGGCCAAGAAGGCCAAGGTCGAGGTAACCGGCGACGACATGCGC
GAAGAAGAGGACATGGCCAAGCGCCGTTCGCGCCTGAAGGCGTTGATCACGCTCGGCAAG
ACCCGCGGCTACCTGACGCAGGTCGAGATCTCCGACCACCTGCCCGACAAGCTGGTCGAC
GCCGAGACGATGGAAGTCGTGATCGCCATGTTGAACGACATGGGCATCGCGGTCTACGAG
CAGACGCCCGACGCCGAGACGCTGTTCCAGAACAACGTCACCCCCACCGCCGCCACTGTC
GAGGAAGCCGAGGAAGAAGCCGAGGCCGCGCTCTCCACCGTCGACAGCGAATTCGGCCGC
ACCACCGACCCGGTCCGCATGTACATGCGCGAAATGGGCACCGTCGAGCTGCTGACCCGC
GAAGGCGAGATCGAGATCGCCAAGCGCATCGAAGGCGGCCTGCAGGCCATGATGGAGGCG
ATCAGCGCGTCGCCCGCCACCATCGCCGCGATCCTGGTGATGGGCGAGGACATCCGCGAA
GGCAAGGTCGTCATCTCGACCATCGTCGACGGCTTCTCCAACCCCAACGAAGCCGACGAC
TACGTGGCCGAGGAGGACTTCGACGAGTTCGACGAGGAAGACGACGACGACGGCAAGGGC
GGCTCCAAGGCGCTGACCAAGAAGCTGGAAGAATTGAAGCGCGAGGCGCTGTCGCGCTTC
GAC
>CFBP4874 Xylophilus ampelinus
CACGCCGGCGGCAAGTTCAACCAGAACAGCTACAAGGTCTCGGGCGGCCTGCACGGCGTG
GGCGTGAGTTGCGTCAACGCGCTCAGCAAGATGCTGCGCCTGACGGTGCGGCGCGACGGC
AAGGTCCACGTGCTGGAGTTCAGCAAGGGCTTCGTGCAGAACCGCATCGTCGAGCAGGCG
GGCGGCGTCGAGGTCTCGCCGATGCGCATCATGGGCGACACCGAGAAGCGCGGTACCGAG
GTGCACTTCCTGCCCGACAACGAGATCTTCAAGGAAAACGCCGATTTCCACTACGAGATC

CTGGCCAAGCGCCTGCGCGAACTGAGCTTCCTGAACAACGGCGTGCGCATCCGCCTGAAG
GACGAGCGCAGCGGCAAGGAAGACGATTTCTCCGGCGCCGGCGGCGTGCAGGGTTTCGTC
GAATTCATCAACAAGGGCAAGACCATCCTGCACCCCACGCCCTTCTACGCCGCGGGCGAG
AAGCCGGCCGAGACGTACGGCGGCATCCCCGGCACGCACATCGGCGTGGAAGTGTCGATG
CAGTGGAACAGCGGCTACACCGAGCAGGTGCTCTGTTTCACCAACAACATCCCGCAGCGC
GACGGCGGCACCCACCTGACCGGCCTGCGCGCCGCGATGACCCGGATCATCAACAAGTAC
ATCGAAGAAAACGAGCTGGCCAAGAAGGCCAAGGTCGAGGTAACCGGCGACGACATGCGC
GAAGAAGAGGACATGGCCAAGCGCCGTTCGCGCCTGAAGGCGTTGATCACGCTCGGCAAG
ACCCGCGGCTACCTGACGCAGGTCGAGATCTCCGACCACCTGCCCGACAAGCTGGTCGAC
GCCGAGACGATGGAAGTCGTGATCGCCATGTTGAACGACATGGGCATCGCGGTCTACGAG
CAGACGCCCGACGCCGAGACGCTGTTCCAGAACAACGTCACCCCCACCGCCGCCACTGTC
GAGGAAGCCGAGGAAGAAGCCGAGGCCGCGCTCTCCACCGTCGACAGCGAATTCGGCCGC
ACCACCGACCCGGTCCGCATGTACATGCGCGAAATGGGCACCGTCGAGCTGCTGACCCGC
GAAGGCGAGATCGAGATCGCCAAGCGCATCGAAGGCGGCCTGCAGGCCATGATGGAGGCG
ATCAGCGCGTCGCCCGCCACCATCGCCGCGATCCTGGTGATGGGCGAGGACATCCGCGAA
GGCAAGGTCGTCATCTCGACCATCGTCGACGGCTTCTCCAACCCCAACGAAGCCGACGAC
TACGTGGCCGAGGAGGACTTCGACGAGTTCGACGAGGAAGACGACGACGACGGCAAGGGC
GGCTCCAAGGCGCTGACCAAGAAGCTGGAAGAATTGAAGCGCGAGGCGCTGTCGCGCTTC
GAC
>CFBP4875 Xylophilus ampelinus
CACGCCGGCGGCAAGTTCAACCAGAACAGCTACAAGGTCTCGGGCGGCCTGCACGGCGTG
GGCGTGAGTTGCGTCAACGCGCTCAGCAAGATGCTGCGCCTGACGGTGCGGCGCGACGGC
AAGGTCCACGTGCTGGAGTTCAGCAAGGGCTTCGTGCAGAACCGCATCGTCGAGCAGGCG
GGCGGCGTCGAGGTCTCGCCGATGCGCATCATGGGCGACACCGAGAAGCGCGGTACCGAG
GTGCACTTCCTGCCCGACAACGAGATCTTCAAGGAAAACGCCGATTTCCACTACGAGATC
CTGGCCAAGCGCCTGCGCGAACTGAGCTTCCTGAACAACGGCGTGCGCATCCGCCTGAAG
GACGAGCGCAGCGGCAAGGAAGACGATTTCTCCGGCGCCGGCGGCGTGCAGGGTTTCGTC
GAATTCATCAACAAGGGCAAGACCATCCTGCACCCCACGCCCTTCTACGCCGCGGGCGAG
AAGCCGGCCGAGACGTACGGCGGCATCCCCGGCACGCACATCGGCGTGGAAGTGTCGATG
CAGTGGAACAGCGGCTACACCGAGCAGGTGCTCTGTTTCACCAACAACATCCCGCAGCGC
GACGGCGGCACCCACCTGACCGGCCTGCGCGCCGCGATGACCCGGATCATCAACAAGTAC
ATCGAAGAAAACGAGCTGGCCAAGAAGGCCAAGGTCGAGGTAACCGGCGACGACATGCGC
GAAGAAGAGGACATGGCCAAGCGCCGTTCGCGCCTGAAGGCGTTGATCACGCTCGGCAAG
ACCCGCGGCTACCTGACGCAGGTCGAGATCTCCGACCACCTGCCCGACAAGCTGGTCGAC
GCCGAGACGATGGAAGTCGTGATCGCCATGTTGAACGACATGGGCATCGCGGTCTACGAG
CAGACGCCCGACGCCGAGACGCTGTTCCAGAACAACGTCACCCCCACCGCCGCCACTGTC
GAGGAAGCCGAGGAAGAAGCCGAGGCCGCGCTCTCCACCGTCGACAGCGAATTCGGCCGC
ACCACCGACCCGGTCCGCATGTACATGCGCGAAATGGGCACCGTCGAGCTGCTGACCCGC
GAAGGCGAGATCGAGATCGCCAAGCGCATCGAAGGCGGCCTGCAGGCCATGATGGAGGCG
ATCAGCGCGTCGCCCGCCACCATCGCCGCGATCCTGGTGATGGGCGAGGACATCCGCGAA
GGCAAGGTCGTCATCTCGACCATCGTCGACGGCTTCTCCAACCCCAACGAAGCCGACGAC
TACGTGGCCGAGGAGGACTTCGACGAGTTCGACGAGGAAGACGACGACGACGGCAAGGGC
GGCTCCAAGGCGCTGACCAAGAAGCTGGAAGAATTGAAGCGCGAGGCGCTGTCGCGCTTC
GAC
>CFBP4876 Xylophilus ampelinus
CACGCCGGCGGCAAGTTCAACCAGAACAGCTACAAGGTCTCGGGCGGCCTGCACGGCGTG
GGCGTGAGTTGCGTCAACGCGCTCAGCAAGATGCTGCGCCTGACGGTGCGGCGCGACGGC
AAGGTCCACGTGCTGGAGTTCAGCAAGGGCTTCGTGCAGAACCGCATCGTCGAGCAGGCG
GGCGGCGTCGAGGTCTCGCCGATGCGCATCATGGGCGACACCGAGAAGCGCGGTACCGAG
GTGCACTTCCTGCCCGACAACGAGATCTTCAAGGAAAACGCCGATTTCCACTACGAGATC
CTGGCCAAGCGCCTGCGCGAACTGAGCTTCCTGAACAACGGCGTGCGCATCCGCCTGAAG
GACGAGCGCAGCGGCAAGGAAGACGATTTCTCCGGCGCCGGCGGCGTGCAGGGTTTCGTC
GAATTCATCAACAAGGGCAAGACCATCCTGCACCCCACGCCCTTCTACGCCGCGGGCGAG
AAGCCGGCCGAGACGTACGGCGGCATCCCCGGCACGCACATCGGCGTGGAAGTGTCGATG
CAGTGGAACAGCGGCTACACCGAGCAGGTGCTCTGTTTCACCAACAACATCCCGCAGCGC
GACGGCGGCACCCACCTGACCGGCCTGCGCGCCGCGATGACCCGGATCATCAACAAGTAC
ATCGAAGAAAACGAGCTGGCCAAGAAGGCCAAGGTCGAGGTAACCGGCGACGACATGCGC
GAAGAAGAGGACATGGCCAAGCGCCGTTCGCGCCTGAAGGCGTTGATCACGCTCGGCAAG

ACCCGCGGCTACCTGACGCAGGTCGAGATCTCCGACCACCTGCCCGACAAGCTGGTCGAC
GCCGAGACGATGGAAGTCGTGATCGCCATGTTGAACGACATGGGCATCGCGGTCTACGAG
CAGACGCCCGACGCCGAGACGCTGTTCCAGAACAACGTCACCCCCACCGCCGCCACTGTC
GAGGAAGCCGAGGAAGAAGCCGAGGCCGCGCTCTCCACCGTCGACAGCGAATTCGGCCGC
ACCACCGACCCGGTCCGCATGTACATGCGCGAAATGGGCACCGTCGAGCTGCTGACCCGC
GAAGGCGAGATCGAGATCGCCAAGCGCATCGAAGGCGGCCTGCAGGCCATGATGGAGGCG
ATCAGCGCGTCGCCCGCCACCATCGCCGCGATCCTGGTGATGGGCGAGGACATCCGCGAA
GGCAAGGTCGTCATCTCGACCATCGTCGACGGCTTCTCCAACCCCAACGAAGCCGACGAC
TACGTGGCCGAGGAGGACTTCGACGAGTTCGACGAGGAAGACGACGACGACGGCAAGGGC
GGCTCCAAGGCGCTGACCAAGAAGCTGGAAGAATTGAAGCGCGAGGCGCTGTCGCGCTTC
GAC
>CFBP4877 Xylophilus ampelinus
CACGCCGGCGGCAAGTTCAACCAGAACAGCTACAAGGTCTCGGGCGGCCTGCACGGCGTG
GGCGTGAGTTGCGTCAACGCGCTCAGCAAGATGCTGCGCCTGACGGTGCGGCGCGACGGC
AAGGTCCACGTGCTGGAGTTCAGCAAGGGCTTCGTGCAGAACCGCATCGTCGAGCAGGCG
GGCGGCGTCGAGGTCTCGCCGATGCGCATCATGGGCGACACCGAGAAGCGCGGTACCGAG
GTGCACTTCCTGCCCGACAACGAGATCTTCAAGGAAAACGCCGATTTCCACTACGAGATC
CTGGCCAAGCGCCTGCGCGAACTGAGCTTCCTGAACAACGGCGTGCGCATCCGCCTGAAG
GACGAGCGCAGCGGCAAGGAAGACGATTTCTCCGGCGCCGGCGGCGTGCAGGGTTTCGTC
GAATTCATCAACAAGGGCAAGACCATCCTGCACCCCACGCCCTTCTACGCCGCGGGCGAG
AAGCCGGCCGAGACGTACGGCGGCATCCCCGGCACGCACATCGGCGTGGAAGTGTCGATG
CAGTGGAACAGCGGCTACACCGAGCAGGTGCTCTGTTTCACCAACAACATCCCGCAGCGC
GACGGCGGCACCCACCTGACCGGCCTGCGCGCCGCGATGACCCGGATCATCAACAAGTAC
ATCGAAGAAAACGAGCTGGCCAAGAAGGCCAAGGTCGAGGTAACCGGCGACGACATGCGC
GAAGAAGAGGACATGGCCAAGCGCCGTTCGCGCCTGAAGGCGTTGATCACGCTCGGCAAG
ACCCGCGGCTACCTGACGCAGGTCGAGATCTCCGACCACCTGCCCGACAAGCTGGTCGAC
GCCGAGACGATGGAAGTCGTGATCGCCATGTTGAACGACATGGGCATCGCGGTCTACGAG
CAGACGCCCGACGCCGAGACGCTGTTCCAGAACAACGTCACCCCCACCGCCGCCACTGTC
GAGGAAGCCGAGGAAGAAGCCGAGGCCGCGCTCTCCACCGTCGACAGCGAATTCGGCCGC
ACCACCGACCCGGTCCGCATGTACATGCGCGAAATGGGCACCGTCGAGCTGCTGACCCGC
GAAGGCGAGATCGAGATCGCCAAGCGCATCGAAGGCGGCCTGCAGGCCATGATGGAGGCG
ATCAGCGCGTCGCCCGCCACCATCGCCGCGATCCTGGTGATGGGCGAGGACATCCGCGAA
GGCAAGGTCGTCATCTCGACCATCGTCGACGGCTTCTCCAACCCCAACGAAGCCGACGAC
TACGTGGCCGAGGAGGACTTCGACGAGTTCGACGAGGAAGACGACGACGACGGCAAGGGC
GGCTCCAAGGCGCTGACCAAGAAGCTGGAAGAATTGAAGCGCGAGGCGCTGTCGCGCTTC
GAC
>CFBP4878 Xylophilus ampelinus
CACGCCGGCGGCAAGTTCAACCAGAACAGCTACAAGGTCTCGGGCGGCCTGCACGGCGTG
GGCGTGAGTTGCGTCAACGCGCTCAGCAAGATGCTGCGCCTGACGGTGCGGCGCGACGGC
AAGGTCCACGTGCTGGAGTTCAGCAAGGGCTTCGTGCAGAACCGCATCGTCGAGCAGGCG
GGCGGCGTCGAGGTCTCGCCGATGCGCATCATGGGCGACACCGAGAAGCGCGGTACCGAG
GTGCACTTCCTGCCCGACAACGAGATCTTCAAGGAAAACGCCGATTTCCACTACGAGATC
CTGGCCAAGCGCCTGCGCGAACTGAGCTTCCTGAACAACGGCGTGCGCATCCGCCTGAAG
GACGAGCGCAGCGGCAAGGAAGACGATTTCTCCGGCGCCGGCGGCGTGCAGGGTTTCGTC
GAATTCATCAACAAGGGCAAGACCATCCTGCACCCCACGCCCTTCTACGCCGCGGGCGAG
AAGCCGGCCGAGACGTACGGCGGCATCCCCGGCACGCACATCGGCGTGGAAGTGTCGATG
CAGTGGAACAGCGGCTACACCGAGCAGGTGCTCTGTTTCACCAACAACATCCCGCAGCGC
GACGGCGGCACCCACCTGACCGGCCTGCGCGCCGCGATGACCCGGATCATCAACAAGTAC
ATCGAAGAAAACGAGCTGGCCAAGAAGGCCAAGGTCGAGGTAACCGGCGACGACATGCGC
GAAGAAGAGGACATGGCCAAGCGCCGTTCGCGCCTGAAGGCGTTGATCACGCTCGGCAAG
ACCCGCGGCTACCTGACGCAGGTCGAGATCTCCGACCACCTGCCCGACAAGCTGGTCGAC
GCCGAGACGATGGAAGTCGTGATCGCCATGTTGAACGACATGGGCATCGCGGTCTACGAG
CAGACGCCCGACGCCGAGACGCTGTTCCAGAACAACGTCACCCCCACCGCCGCCACTGTC
GAGGAAGCCGAGGAAGAAGCCGAGGCCGCGCTCTCCACCGTCGACAGCGAATTCGGCCGC
ACCACCGACCCGGTCCGCATGTACATGCGCGAAATGGGCACCGTCGAGCTGCTGACCCGC
GAAGGCGAGATCGAGATCGCCAAGCGCATCGAAGGCGGCCTGCAGGCCATGATGGAGGCG
ATCAGCGCGTCGCCCGCCACCATCGCCGCGATCCTGGTGATGGGCGAGGACATCCGCGAA
GGCAAGGTCGTCATCTCGACCATCGTCGACGGCTTCTCCAACCCCAACGAAGCCGACGAC

TACGTGGCCGAGGAGGACTTCGACGAGTTCGACGAGGAAGACGACGACGACGGCAAGGGC
GGCTCCAAGGCGCTGACCAAGAAGCTGGAAGAATTGAAGCGCGAGGCGCTGTCGCGCTTC
GAC
>CFBP4879 Xylophilus ampelinus
CACGCCGGCGGCAAGTTCAACCAGAACAGCTACAAGGTCTCGGGCGGCCTGCACGGCGTG
GGCGTGAGTTGCGTCAACGCGCTCAGCAAGATGCTGCGCCTGACGGTGCGGCGCGACGGC
AAGGTCCACGTGCTGGAGTTCAGCAAGGGCTTCGTGCAGAACCGCATCGTCGAGCAGGCG
GGCGGCGTCGAGGTCTCGCCGATGCGCATCATGGGCGACACCGAGAAGCGCGGTACCGAG
GTGCACTTCCTGCCCGACAACGAGATCTTCAAGGAAAACGCCGATTTCCACTACGAGATC
CTGGCCAAGCGCCTGCGCGAACTGAGCTTCCTGAACAACGGCGTGCGCATCCGCCTGAAG
GACGAGCGCAGCGGCAAGGAAGACGATTTCTCCGGCGCCGGCGGCGTGCAGGGTTTCGTC
GAATTCATCAACAAGGGCAAGACCATCCTGCACCCCACGCCCTTCTACGCCGCGGGCGAG
AAGCCGGCCGAGACGTACGGCGGCATCCCCGGCACGCACATCGGCGTGGAAGTGTCGATG
CAGTGGAACAGCGGCTACACCGAGCAGGTGCTCTGTTTCACCAACAACATCCCGCAGCGC
GACGGCGGCACCCACCTGACCGGCCTGCGCGCCGCGATGACCCGGATCATCAACAAGTAC
ATCGAAGAAAACGAGCTGGCCAAGAAGGCCAAGGTCGAGGTAACCGGCGACGACATGCGC
GAAGAAGAGGACATGGCCAAGCGCCGTTCGCGCCTGAAGGCGTTGATCACGCTCGGCAAG
ACCCGCGGCTACCTGACGCAGGTCGAGATCTCCGACCACCTGCCCGACAAGCTGGTCGAC
GCCGAGACGATGGAAGTCGTGATCGCCATGTTGAACGACATGGGCATCGCGGTCTACGAG
CAGACGCCCGACGCCGAGACGCTGTTCCAGAACAACGTCACCCCCACCGCCGCCACTGTC
GAGGAAGCCGAGGAAGAAGCCGAGGCCGCGCTCTCCACCGTCGACAGCGAATTCGGCCGC
ACCACCGACCCGGTCCGCATGTACATGCGCGAAATGGGCACCGTCGAGCTGCTGACCCGC
GAAGGCGAGATCGAGATCGCCAAGCGCATCGAAGGCGGCCTGCAGGCCATGATGGAGGCG
ATCAGCGCGTCGCCCGCCACCATCGCCGCGATCCTGGTGATGGGCGAGGACATCCGCGAA
GGCAAGGTCGTCATCTCGACCATCGTCGACGGCTTCTCCAACCCCAACGAAGCCGACGAC
TACGTGGCCGAGGAGGACTTCGACGAGTTCGACGAGGAAGACGACGACGACGGCAAGGGC
GGCTCCAAGGCGCTGACCAAGAAGCTGGAAGAATTGAAGCGCGAGGCGCTGTCGCGCTTC
GAC
>CFBP4880 Xylophilus ampelinus
CACGCCGGCGGCAAGTTCAACCAGAACAGCTACAAGGTCTCGGGCGGCCTGCACGGCGTG
GGCGTGAGTTGCGTCAACGCGCTCAGCAAGATGCTGCGCCTGACGGTGCGGCGCGACGGC
AAGGTCCACGTGCTGGAGTTCAGCAAGGGCTTCGTGCAGAACCGCATCGTCGAGCAGGCG
GGCGGCGTCGAGGTCTCGCCGATGCGCATCATGGGCGACACCGAGAAGCGCGGTACCGAG
GTGCACTTCCTGCCCGACAACGAGATCTTCAAGGAAAACGCCGATTTCCACTACGAGATC
CTGGCCAAGCGCCTGCGCGAACTGAGCTTCCTGAACAACGGCGTGCGCATCCGCCTGAAG
GACGAGCGCAGCGGCAAGGAAGACGATTTCTCCGGCGCCGGCGGCGTGCAGGGTTTCGTC
GAATTCATCAACAAGGGCAAGACCATCCTGCACCCCACGCCCTTCTACGCCGCGGGCGAG
AAGCCGGCCGAGACGTACGGCGGCATCCCCGGCACGCACATCGGCGTGGAAGTGTCGATG
CAGTGGAACAGCGGCTACACCGAGCAGGTGCTCTGTTTCACCAACAACATCCCGCAGCGC
GACGGCGGCACCCACCTGACCGGCCTGCGCGCCGCGATGACCCGGATCATCAACAAGTAC
ATCGAAGAAAACGAGCTGGCCAAGAAGGCCAAGGTCGAGGTAACCGGCGACGACATGCGC
GAAGAAGAGGACATGGCCAAGCGCCGTTCGCGCCTGAAGGCGTTGATCACGCTCGGCAAG
ACCCGCGGCTACCTGACGCAGGTCGAGATCTCCGACCACCTGCCCGACAAGCTGGTCGAC
GCCGAGACGATGGAAGTCGTGATCGCCATGTTGAACGACATGGGCATCGCGGTCTACGAG
CAGACGCCCGACGCCGAGACGCTGTTCCAGAACAACGTCACCCCCACCGCCGCCACTGTC
GAGGAAGCCGAGGAAGAAGCCGAGGCCGCGCTCTCCACCGTCGACAGCGAATTCGGCCGC
ACCACCGACCCGGTCCGCATGTACATGCGCGAAATGGGCACCGTCGAGCTGCTGACCCGC
GAAGGCGAGATCGAGATCGCCAAGCGCATCGAAGGCGGCCTGCAGGCCATGATGGAGGCG
ATCAGCGCGTCGCCCGCCACCATCGCCGCGATCCTGGTGATGGGCGAGGACATCCGCGAA
GGCAAGGTCGTCATCTCGACCATCGTCGACGGCTTCTCCAACCCCAACGAAGCCGACGAC
TACGTGGCCGAGGAGGACTTCGACGAGTTCGACGAGGAAGACGACGACGACGGCAAGGGC
GGCTCCAAGGCGCTGACCAAGAAGCTGGAAGAATTGAAGCGCGAGGCGCTGTCGCGCTTC
GAC
>CFBP4881 Xylophilus ampelinus
CACGCCGGCGGCAAGTTCAACCAGAACAGCTACAAGGTCTCGGGCGGCCTGCACGGCGTG
GGCGTGAGTTGCGTCAACGCGCTCAGCAAGATGCTGCGCCTGACGGTGCGGCGCGACGGC
AAGGTCCACGTGCTGGAGTTCAGCAAGGGCTTCGTGCAGAACCGCATCGTCGAGCAGGCG
GGCGGCGTCGAGGTCTCGCCGATGCGCATCATGGGCGACACCGAGAAGCGCGGTACCGAG

GTGCACTTCCTGCCCGACAACGAGATCTTCAAGGAAAACGCCGATTTCCACTACGAGATC
CTGGCCAAGCGCCTGCGCGAACTGAGCTTCCTGAACAACGGCGTGCGCATCCGCCTGAAG
GACGAGCGCAGCGGCAAGGAAGACGATTTCTCCGGCGCCGGCGGCGTGCAGGGTTTCGTC
GAATTCATCAACAAGGGCAAGACCATCCTGCACCCCACGCCCTTCTACGCCGCGGGCGAG
AAGCCGGCCGAGACGTACGGCGGCATCCCCGGCACGCACATCGGCGTGGAAGTGTCGATG
CAGTGGAACAGCGGCTACACCGAGCAGGTGCTCTGTTTCACCAACAACATCCCGCAGCGC
GACGGCGGCACCCACCTGACCGGCCTGCGCGCCGCGATGACCCGGATCATCAACAAGTAC
ATCGAAGAAAACGAGCTGGCCAAGAAGGCCAAGGTCGAGGTAACCGGCGACGACATGCGC
GAAGAAGAGGACATGGCCAAGCGCCGTTCGCGCCTGAAGGCGTTGATCACGCTCGGCAAG
ACCCGCGGCTACCTGACGCAGGTCGAGATCTCCGACCACCTGCCCGACAAGCTGGTCGAC
GCCGAGACGATGGAAGTCGTGATCGCCATGTTGAACGACATGGGCATCGCGGTCTACGAG
CAGACGCCCGACGCCGAGACGCTGTTCCAGAACAACGTCACCCCCACCGCCGCCACTGTC
GAGGAAGCCGAGGAAGAAGCCGAGGCCGCGCTCTCCACCGTCGACAGCGAATTCGGCCGC
ACCACCGACCCGGTCCGCATGTACATGCGCGAAATGGGCACCGTCGAGCTGCTGACCCGC
GAAGGCGAGATCGAGATCGCCAAGCGCATCGAAGGCGGCCTGCAGGCCATGATGGAGGCG
ATCAGCGCGTCGCCCGCCACCATCGCCGCGATCCTGGTGATGGGCGAGGACATCCGCGAA
GGCAAGGTCGTCATCTCGACCATCGTCGACGGCTTCTCCAACCCCAACGAAGCCGACGAC
TACGTGGCCGAGGAGGACTTCGACGAGTTCGACGAGGAAGACGACGACGACGGCAAGGGC
GGCTCCAAGGCGCTGACCAAGAAGCTGGAAGAATTGAAGCGCGAGGCGCTGTCGCGCTTC
GAC
>CFBP4882 Xylophilus ampelinus
CACGCCGGCGGCAAGTTCAACCAGAACAGCTACAAGGTCTCGGGCGGCCTGCACGGCGTG
GGCGTGAGTTGCGTCAACGCGCTCAGCAAGATGCTGCGCCTGACGGTGCGGCGCGACGGC
AAGGTCCACGTGCTGGAGTTCAGCAAGGGCTTCGTGCAGAACCGCATCGTCGAGCAGGCG
GGCGGCGTCGAGGTCTCGCCGATGCGCATCATGGGCGACACCGAGAAGCGCGGTACCGAG
GTGCACTTCCTGCCCGACAACGAGATCTTCAAGGAAAACGCCGATTTCCACTACGAGATC
CTGGCCAAGCGCCTGCGCGAACTGAGCTTCCTGAACAACGGCGTGCGCATCCGCCTGAAG
GACGAGCGCAGCGGCAAGGAAGACGATTTCTCCGGCGCCGGCGGCGTGCAGGGTTTCGTC
GAATTCATCAACAAGGGCAAGACCATCCTGCACCCCACGCCCTTCTACGCCGCGGGCGAG
AAGCCGGCCGAGACGTACGGCGGCATCCCCGGCACGCACATCGGCGTGGAAGTGTCGATG
CAGTGGAACAGCGGCTACACCGAGCAGGTGCTCTGTTTCACCAACAACATCCCGCAGCGC
GACGGCGGCACCCACCTGACCGGCCTGCGCGCCGCGATGACCCGGATCATCAACAAGTAC
ATCGAAGAAAACGAGCTGGCCAAGAAGGCCAAGGTCGAGGTAACCGGCGACGACATGCGC
GAAGAAGAGGACATGGCCAAGCGCCGTTCGCGCCTGAAGGCGTTGATCACGCTCGGCAAG
ACCCGCGGCTACCTGACGCAGGTCGAGATCTCCGACCACCTGCCCGACAAGCTGGTCGAC
GCCGAGACGATGGAAGTCGTGATCGCCATGTTGAACGACATGGGCATCGCGGTCTACGAG
CAGACGCCCGACGCCGAGACGCTGTTCCAGAACAACGTCACCCCCACCGCCGCCACTGTC
GAGGAAGCCGAGGAAGAAGCCGAGGCCGCGCTCTCCACCGTCGACAGCGAATTCGGCCGC
ACCACCGACCCGGTCCGCATGTACATGCGCGAAATGGGCACCGTCGAGCTGCTGACCCGC
GAAGGCGAGATCGAGATCGCCAAGCGCATCGAAGGCGGCCTGCAGGCCATGATGGAGGCG
ATCAGCGCGTCGCCCGCCACCATCGCCGCGATCCTGGTGATGGGCGAGGACATCCGCGAA
GGCAAGGTCGTCATCTCGACCATCGTCGACGGCTTCTCCAACCCCAACGAAGCCGACGAC
TACGTGGCCGAGGAGGACTTCGACGAGTTCGACGAGGAAGACGACGACGACGGCAAGGGC
GGCTCCAAGGCGCTGACCAAGAAGCTGGAAGAATTGAAGCGCGAGGCGCTGTCGCGCTTC
GAC
>CFBP4883 Xylophilus ampelinus
CACGCCGGCGGCAAGTTCAACCAGAACAGCTACAAGGTCTCGGGCGGCCTGCACGGCGTG
GGCGTGAGTTGCGTCAACGCGCTCAGCAAGATGCTGCGCCTGACGGTGCGGCGCGACGGC
AAGGTCCACGTGCTGGAGTTCAGCAAGGGCTTCGTGCAGAACCGCATCGTCGAGCAGGCG
GGCGGCGTCGAGGTCTCGCCGATGCGCATCATGGGCGACACCGAGAAGCGCGGTACCGAG
GTGCACTTCCTGCCCGACAACGAGATCTTCAAGGAAAACGCCGATTTCCACTACGAGATC
CTGGCCAAGCGCCTGCGCGAACTGAGCTTCCTGAACAACGGCGTGCGCATCCGCCTGAAG
GACGAGCGCAGCGGCAAGGAAGACGATTTCTCCGGCGCCGGCGGCGTGCAGGGTTTCGTC
GAATTCATCAACAAGGGCAAGACCATCCTGCACCCCACGCCCTTCTACGCCGCGGGCGAG
AAGCCGGCCGAGACGTACGGCGGCATCCCCGGCACGCACATCGGCGTGGAAGTGTCGATG
CAGTGGAACAGCGGCTACACCGAGCAGGTGCTCTGTTTCACCAACAACATCCCGCAGCGC
GACGGCGGCACCCACCTGACCGGCCTGCGCGCCGCGATGACCCGGATCATCAACAAGTAC
ATCGAAGAAAACGAGCTGGCCAAGAAGGCCAAGGTCGAGGTAACCGGCGACGACATGCGC

GAAGAAGAGGACATGGCCAAGCGCCGTTCGCGCCTGAAGGCGTTGATCACGCTCGGCAAG
ACCCGCGGCTACCTGACGCAGGTCGAGATCTCCGACCACCTGCCCGACAAGCTGGTCGAC
GCCGAGACGATGGAAGTCGTGATCGCCATGTTGAACGACATGGGCATCGCGGTCTACGAG
CAGACGCCCGACGCCGAGACGCTGTTCCAGAACAACGTCACCCCCACCGCCGCCACTGTC
GAGGAAGCCGAGGAAGAAGCCGAGGCCGCGCTCTCCACCGTCGACAGCGAATTCGGCCGC
ACCACCGACCCGGTCCGCATGTACATGCGCGAAATGGGCACCGTCGAGCTGCTGACCCGC
GAAGGCGAGATCGAGATCGCCAAGCGCATCGAAGGCGGCCTGCAGGCCATGATGGAGGCG
ATCAGCGCGTCGCCCGCCACCATCGCCGCGATCCTGGTGATGGGCGAGGACATCCGCGAA
GGCAAGGTCGTCATCTCGACCATCGTCGACGGCTTCTCCAACCCCAACGAAGCCGACGAC
TACGTGGCCGAGGAGGACTTCGACGAGTTCGACGAGGAAGACGACGACGACGGCAAGGGC
GGCTCCAAGGCGCTGACCAAGAAGCTGGAAGAATTGAAGCGCGAGGCGCTGTCGCGCTTC
GAC

>CFBP4947 Xylophilus ampelinus

CACGCCGGCGGCAAGTTCAACCAGAACAGCTACAAGGTCTCGGGCGGCCTGCACGGCGTG
GGCGTGAGTTGCGTCAACGCGCTCAGCAAGATGCTGCGCCTGACGGTGCGGCGCGACGGC
AAGGTCCACGTGCTGGAGTTCAGCAAGGGCTTCGTGCAGAACCGCATCGTCGAGCAGGCG
GGCGGCGTCGAGGTCTCGCCGATGCGCATCATGGGCGACACCGAGAAGCGCGGTACCGAG
GTGCACTTCCTGCCCGACAACGAGATCTTCAAGGAAAACGCCGATTTCCACTACGAGATC
CTGGCCAAGCGCCTGCGCGAACTGAGCTTCCTGAACAACGGCGTGCGCATCCGCCTGAAG
GACGAGCGCAGCGGCAAGGAAGACGATTTCTCCGGCGCCGGCGGCGTGCAGGGTTTCGTC
GAATTCATCAACAAGGGCAAGACCATCCTGCACCCCACGCCCTTCTACGCCGCGGGCGAG
AAGCCGGCCGAGACGTACGGCGGCATCCCCGGCACGCACATCGGCGTGGAAGTGTCGATG
CAGTGGAACAGCGGCTACACCGAGCAGGTGCTCTGTTTCACCAACAACATCCCGCAGCGC
GACGGCGGCACCCACCTGACCGGCCTGCGCGCCGCGATGACCCGGATCATCAACAAGTAC
ATCGAAGAAAACGAGCTGGCCAAGAAGGCCAAGGTCGAGGTAACCGGCGACGACATGCGC
GAAGAAGAGGACATGGCCAAGCGCCGTTCGCGCCTGAAGGCGTTGATCACGCTCGGCAAG
ACCCGCGGCTACCTGACGCAGGTCGAGATCTCCGACCACCTGCCCGACAAGCTGGTCGAC
GCCGAGACGATGGAAGTCGTGATCGCCATGTTGAACGACATGGGCATCGCGGTCTACGAG
CAGACGCCCGACGCCGAGACGCTGTTCCAGAACAACGTCACCCCCACCGCCGCCACTGTC
GAGGAAGCCGAGGAAGAAGCCGAGGCCGCGCTCTCCACCGTCGACAGCGAATTCGGCCGC
ACCACCGACCCGGTCCGCATGTACATGCGCGAAATGGGCACCGTCGAGCTGCTGACCCGC
GAAGGCGAGATCGAGATCGCCAAGCGCATCGAAGGCGGCCTGCAGGCCATGATGGAGGCG
ATCAGCGCGTCGCCCGCCACCATCGCCGCGATCCTGGTGATGGGCGAGGACATCCGCGAA
GGCAAGGTCGTCATCTCGACCATCGTCGACGGCTTCTCCAACCCCAACGAAGCCGACGAC
TACGTGGCCGAGGAGGACTTCGACGAGTTCGACGAGGAAGACGACGACGACGGCAAGGGC
GGCTCCAAGGCGCTGACCAAGAAGCTGGAAGAATTGAAGCGCGAGGCGCTGTCGCGCTTC
GAC

>CFBP4949 Xylophilus ampelinus

CACGCCGGCGGCAAGTTCAACCAGAACAGCTACAAGGTCTCGGGCGGCCTGCACGGCGTG
GGCGTGAGTTGCGTCAACGCGCTCAGCAAGATGCTGCGCCTGACGGTGCGGCGCGACGGC
AAGGTCCACGTGCTGGAGTTCAGCAAGGGCTTCGTGCAGAACCGCATCGTCGAGCAGGCG
GGCGGCGTCGAGGTCTCGCCGATGCGCATCATGGGCGACACCGAGAAGCGCGGTACCGAG
GTGCACTTCCTGCCCGACAACGAGATCTTCAAGGAAAACGCCGATTTCCACTACGAGATC
CTGGCCAAGCGCCTGCGCGAACTGAGCTTCCTGAACAACGGCGTGCGCATCCGCCTGAAG
GACGAGCGCAGCGGCAAGGAAGACGATTTCTCCGGCGCCGGCGGCGTGCAGGGTTTCGTC
GAATTCATCAACAAGGGCAAGACCATCCTGCACCCCACGCCCTTCTACGCCGCGGGCGAG
AAGCCGGCCGAGACGTACGGCGGCATCCCCGGCACGCACATCGGCGTGGAAGTGTCGATG
CAGTGGAACAGCGGCTACACCGAGCAGGTGCTCTGTTTCACCAACAACATCCCGCAGCGC
GACGGCGGCACCCACCTGACCGGCCTGCGCGCCGCGATGACCCGGATCATCAACAAGTAC
ATCGAAGAAAACGAGCTGGCCAAGAAGGCCAAGGTCGAGGTAACCGGCGACGACATGCGC
GAAGAAGAGGACATGGCCAAGCGCCGTTCGCGCCTGAAGGCGTTGATCACGCTCGGCAAG
ACCCGCGGCTACCTGACGCAGGTCGAGATCTCCGACCACCTGCCCGACAAGCTGGTCGAC
GCCGAGACGATGGAAGTCGTGATCGCCATGTTGAACGACATGGGCATCGCGGTCTACGAG
CAGACGCCCGACGCCGAGACGCTGTTCCAGAACAACGTCACCCCCACCGCCGCCACTGTC
GAGGAAGCCGAGGAAGAAGCCGAGGCCGCGCTCTCCACCGTCGACAGCGAATTCGGCCGC
ACCACCGACCCGGTCCGCATGTACATGCGCGAAATGGGCACCGTCGAGCTGCTGACCCGC
GAAGGCGAGATCGAGATCGCCAAGCGCATCGAAGGCGGCCTGCAGGCCATGATGGAGGCG
ATCAGCGCGTCGCCCGCCACCATCGCCGCGATCCTGGTGATGGGCGAGGACATCCGCGAA

GGCAAGGTCGTCATCTCGACCATCGTCGACGGCTTCTCCAACCCCAACGAAGCCGACGAC
TACGTGGCCGAGGAGGACTTCGACGAGTTCGACGAGGAAGACGACGACGACGGCAAGGGC
GGCTCCAAGGCGCTGACCAAGAAGCTGGAAGAATTGAAGCGCGAGGCGCTGTCGCGCTTC
GAC
>CFBP4977 Xylophilus ampelinus
CACGCCGGCGGCAAGTTCAACCAGAACAGCTACAAGGTCTCGGGCGGCCTGCACGGCGTG
GGCGTGAGTTGCGTCAACGCGCTCAGCAAGATGCTGCGCCTGACGGTGCGGCGCGACGGC
AAGGTCCACGTGCTGGAGTTCAGCAAGGGCTTCGTGCAGAACCGCATCGTCGAGCAGGCG
GGCGGCGTCGAGGTCTCGCCGATGCGCATCATGGGCGACACCGAGAAGCGCGGTACCGAG
GTGCACTTCCTGCCCGACAACGAGATCTTCAAGGAAAACGCCGATTTCCACTACGAGATC
CTGGCCAAGCGCCTGCGCGAACTGAGCTTCCTGAACAACGGCGTGCGCATCCGCCTGAAG
GACGAGCGCAGCGGCAAGGAAGACGATTTCTCCGGCGCCGGCGGCGTGCAGGGTTTCGTC
GAATTCATCAACAAGGGCAAGACCATCCTGCACCCCACGCCCTTCTACGCCGCGGGCGAG
AAGCCGGCCGAGACGTACGGCGGCATCCCCGGCACGCACATCGGCGTGGAAGTGTCGATG
CAGTGGAACAGCGGCTACACCGAGCAGGTGCTCTGTTTCACCAACAACATCCCGCAGCGC
GACGGCGGCACCCACCTGACCGGCCTGCGCGCCGCGATGACCCGGATCATCAACAAGTAC
ATCGAAGAAAACGAGCTGGCCAAGAAGGCCAAGGTCGAGGTAACCGGCGACGACATGCGC
GAAGAAGAGGACATGGCCAAGCGCCGTTCGCGCCTGAAGGCGTTGATCACGCTCGGCAAG
ACCCGCGGCTACCTGACGCAGGTCGAGATCTCCGACCACCTGCCCGACAAGCTGGTCGAC
GCCGAGACGATGGAAGTCGTGATCGCCATGTTGAACGACATGGGCATCGCGGTCTACGAG
CAGACGCCCGACGCCGAGACGCTGTTCCAGAACAACGTCACCCCCACCGCCGCCACTGTC
GAGGAAGCCGAGGAAGAAGCCGAGGCCGCGCTCTCCACCGTCGACAGCGAATTCGGCCGC
ACCACCGACCCGGTCCGCATGTACATGCGCGAAATGGGCACCGTCGAGCTGCTGACCCGC
GAAGGCGAGATCGAGATCGCCAAGCGCATCGAAGGCGGCCTGCAGGCCATGATGGAGGCG
ATCAGCGCGTCGCCCGCCACCATCGCCGCGATCCTGGTGATGGGCGAGGACATCCGCGAA
GGCAAGGTCGTCATCTCGACCATCGTCGACGGCTTCTCCAACCCCAACGAAGCCGACGAC
TACGTGGCCGAGGAGGACTTCGACGAGTTCGACGAGGAAGACGACGACGACGGCAAGGGC
GGCTCCAAGGCGCTGACCAAGAAGCTGGAAGAATTGAAGCGCGAGGCGCTGTCGCGCTTC
GAC
>CFBP4978 Xylophilus ampelinus
CACGCCGGCGGCAAGTTCAACCAGAACAGCTACAAGGTCTCGGGCGGCCTGCACGGCGTG
GGCGTGAGTTGCGTCAACGCGCTCAGCAAGATGCTGCGCCTGACGGTGCGGCGCGACGGC
AAGGTCCACGTGCTGGAGTTCAGCAAGGGCTTCGTGCAGAACCGCATCGTCGAGCAGGCG
GGCGGCGTCGAGGTCTCGCCGATGCGCATCATGGGCGACACCGAGAAGCGCGGTACCGAG
GTGCACTTCCTGCCCGACAACGAGATCTTCAAGGAAAACGCCGATTTCCACTACGAGATC
CTGGCCAAGCGCCTGCGCGAACTGAGCTTCCTGAACAACGGCGTGCGCATCCGCCTGAAG
GACGAGCGCAGCGGCAAGGAAGACGATTTCTCCGGCGCCGGCGGCGTGCAGGGTTTCGTC
GAATTCATCAACAAGGGCAAGACCATCCTGCACCCCACGCCCTTCTACGCCGCGGGCGAG
AAGCCGGCCGAGACGTACGGCGGCATCCCCGGCACGCACATCGGCGTGGAAGTGTCGATG
CAGTGGAACAGCGGCTACACCGAGCAGGTGCTCTGTTTCACCAACAACATCCCGCAGCGC
GACGGCGGCACCCACCTGACCGGCCTGCGCGCCGCGATGACCCGGATCATCAACAAGTAC
ATCGAAGAAAACGAGCTGGCCAAGAAGGCCAAGGTCGAGGTAACCGGCGACGACATGCGC
GAAGAAGAGGACATGGCCAAGCGCCGTTCGCGCCTGAAGGCGTTGATCACGCTCGGCAAG
ACCCGCGGCTACCTGACGCAGGTCGAGATCTCCGACCACCTGCCCGACAAGCTGGTCGAC
GCCGAGACGATGGAAGTCGTGATCGCCATGTTGAACGACATGGGCATCGCGGTCTACGAG
CAGACGCCCGACGCCGAGACGCTGTTCCAGAACAACGTCACCCCCACCGCCGCCACTGTC
GAGGAAGCCGAGGAAGAAGCCGAGGCCGCGCTCTCCACCGTCGACAGCGAATTCGGCCGC
ACCACCGACCCGGTCCGCATGTACATGCGCGAAATGGGCACCGTCGAGCTGCTGACCCGC
GAAGGCGAGATCGAGATCGCCAAGCGCATCGAAGGCGGCCTGCAGGCCATGATGGAGGCG
ATCAGCGCGTCGCCCGCCACCATCGCCGCGATCCTGGTGATGGGCGAGGACATCCGCGAA
GGCAAGGTCGTCATCTCGACCATCGTCGACGGCTTCTCCAACCCCAACGAAGCCGACGAC
TACGTGGCCGAGGAGGACTTCGACGAGTTCGACGAGGAAGACGACGACGACGGCAAGGGC
GGCTCCAAGGCGCTGACCAAGAAGCTGGAAGAATTGAAGCGCGAGGCGCTGTCGCGCTTC
GAC
>CFBP4979 Xylophilus ampelinus
CACGCCGGCGGCAAGTTCAACCAGAACAGCTACAAGGTCTCGGGCGGCCTGCACGGCGTG
GGCGTGAGTTGCGTCAACGCGCTCAGCAAGATGCTGCGCCTGACGGTGCGGCGCGACGGC
AAGGTCCACGTGCTGGAGTTCAGCAAGGGCTTCGTGCAGAACCGCATCGTCGAGCAGGCG

GGCGGCGTCGAGGTCTCGCCGATGCGCATCATGGGCGACACCGAGAAGCGCGGTACCGAG
GTGCACTTCCTGCCCGACAACGAGATCTTCAAGGAAAACGCCGATTTCCACTACGAGATC
CTGGCCAAGCGCCTGCGCGAACTGAGCTTCCTGAACAACGGCGTGCGCATCCGCCTGAAG
GACGAGCGCAGCGGCAAGGAAGACGATTTCTCCGGCGCCGGCGGCGTGCAGGGTTTCGTC
GAATTCATCAACAAGGGCAAGACCATCCTGCACCCCACGCCCTTCTACGCCGCGGGCGAG
AAGCCGGCCGAGACGTACGGCGGCATCCCCGGCACGCACATCGGCGTGGAAGTGTCGATG
CAGTGGAACAGCGGCTACACCGAGCAGGTGCTCTGTTTCACCAACAACATCCCGCAGCGC
GACGGCGGCACCCACCTGACCGGCCTGCGCGCCGCGATGACCCGGATCATCAACAAGTAC
ATCGAAGAAAACGAGCTGGCCAAGAAGGCCAAGGTCGAGGTAACCGGCGACGACATGCGC
GAAGAAGAGGACATGGCCAAGCGCCGTTCGCGCCTGAAGGCGTTGATCACGCTCGGCAAG
ACCCGCGGCTACCTGACGCAGGTCGAGATCTCCGACCACCTGCCCGACAAGCTGGTCGAC
GCCGAGACGATGGAAGTCGTGATCGCCATGTTGAACGACATGGGCATCGCGGTCTACGAG
CAGACGCCCGACGCCGAGACGCTGTTCCAGAACAACGTCACCCCCACCGCCGCCACTGTC
GAGGAAGCCGAGGAAGAAGCCGAGGCCGCGCTCTCCACCGTCGACAGCGAATTCGGCCGC
ACCACCGACCCGGTCCGCATGTACATGCGCGAAATGGGCACCGTCGAGCTGCTGACCCGC
GAAGGCGAGATCGAGATCGCCAAGCGCATCGAAGGCGGCCTGCAGGCCATGATGGAGGCG
ATCAGCGCGTCGCCCGCCACCATCGCCGCGATCCTGGTGATGGGCGAGGACATCCGCGAA
GGCAAGGTCGTCATCTCGACCATCGTCGACGGCTTCTCCAACCCCAACGAAGCCGACGAC
TACGTGGCCGAGGAGGACTTCGACGAGTTCGACGAGGAAGACGACGACGACGGCAAGGGC
GGCTCCAAGGCGCTGACCAAGAAGCTGGAAGAATTGAAGCGCGAGGCGCTGTCGCGCTTC
GAC
>CFBP4980 Xylophilus ampelinus
CACGCCGGCGGCAAGTTCAACCAGAACAGCTACAAGGTCTCGGGCGGCCTGCACGGCGTG
GGCGTGAGTTGCGTCAACGCGCTCAGCAAGATGCTGCGCCTGACGGTGCGGCGCGACGGC
AAGGTCCACGTGCTGGAGTTCAGCAAGGGCTTCGTGCAGAACCGCATCGTCGAGCAGGCG
GGCGGCGTCGAGGTCTCGCCGATGCGCATCATGGGCGACACCGAGAAGCGCGGTACCGAG
GTGCACTTCCTGCCCGACAACGAGATCTTCAAGGAAAACGCCGATTTCCACTACGAGATC
CTGGCCAAGCGCCTGCGCGAACTGAGCTTCCTGAACAACGGCGTGCGCATCCGCCTGAAG
GACGAGCGCAGCGGCAAGGAAGACGATTTCTCCGGCGCCGGCGGCGTGCAGGGTTTCGTC
GAATTCATCAACAAGGGCAAGACCATCCTGCACCCCACGCCCTTCTACGCCGCGGGCGAG
AAGCCGGCCGAGACGTACGGCGGCATCCCCGGCACGCACATCGGCGTGGAAGTGTCGATG
CAGTGGAACAGCGGCTACACCGAGCAGGTGCTCTGTTTCACCAACAACATCCCGCAGCGC
GACGGCGGCACCCACCTGACCGGCCTGCGCGCCGCGATGACCCGGATCATCAACAAGTAC
ATCGAAGAAAACGAGCTGGCCAAGAAGGCCAAGGTCGAGGTAACCGGCGACGACATGCGC
GAAGAAGAGGACATGGCCAAGCGCCGTTCGCGCCTGAAGGCGTTGATCACGCTCGGCAAG
ACCCGCGGCTACCTGACGCAGGTCGAGATCTCCGACCACCTGCCCGACAAGCTGGTCGAC
GCCGAGACGATGGAAGTCGTGATCGCCATGTTGAACGACATGGGCATCGCGGTCTACGAG
CAGACGCCCGACGCCGAGACGCTGTTCCAGAACAACGTCACCCCCACCGCCGCCACTGTC
GAGGAAGCCGAGGAAGAAGCCGAGGCCGCGCTCTCCACCGTCGACAGCGAATTCGGCCGC
ACCACCGACCCGGTCCGCATGTACATGCGCGAAATGGGCACCGTCGAGCTGCTGACCCGC
GAAGGCGAGATCGAGATCGCCAAGCGCATCGAAGGCGGCCTGCAGGCCATGATGGAGGCG
ATCAGCGCGTCGCCCGCCACCATCGCCGCGATCCTGGTGATGGGCGAGGACATCCGCGAA
GGCAAGGTCGTCATCTCGACCATCGTCGACGGCTTCTCCAACCCCAACGAAGCCGACGAC
TACGTGGCCGAGGAGGACTTCGACGAGTTCGACGAGGAAGACGACGACGACGGCAAGGGC
GGCTCCAAGGCGCTGACCAAGAAGCTGGAAGAATTGAAGCGCGAGGCGCTGTCGCGCTTC
GAC
>CFBP5787 Xylophilus ampelinus
CACGCCGGCGGCAAGTTCAACCAGAACAGCTACAAGGTCTCGGGCGGCCTGCACGGCGTG
GGCGTGAGTTGCGTCAACGCGCTCAGCAAGATGCTGCGCCTGACGGTGCGGCGCGACGGC
AAGGTCCACGTGCTGGAGTTCAGCAAGGGCTTCGTGCAGAACCGCATCGTCGAGCAGGCG
GGCGGCGTCGAGGTCTCGCCGATGCGCATCATGGGCGACACCGAGAAGCGCGGTACCGAG
GTGCACTTCCTGCCCGACAACGAGATCTTCAAGGAAAACGCCGATTTCCACTACGAGATC
CTGGCCAAGCGCCTGCGCGAACTGAGCTTCCTGAACAACGGCGTGCGCATCCGCCTGAAG
GACGAGCGCAGCGGCAAGGAAGACGATTTCTCCGGCGCCGGCGGCGTGCAGGGTTTCGTC
GAATTCATCAACAAGGGCAAGACCATCCTGCACCCCACGCCCTTCTACGCCGCGGGCGAG
AAGCCGGCCGAGACGTACGGCGGCATCCCCGGCACGCACATCGGCGTGGAAGTGTCGATG
CAGTGGAACAGCGGCTACACCGAGCAGGTGCTCTGTTTCACCAACAACATCCCGCAGCGC
GACGGCGGCACCCACCTGACCGGCCTGCGCGCCGCGATGACCCGGATCATCAACAAGTAC

ATCGAAGAAAACGAGCTGGCCAAGAAGGCCAAGGTCGAGGTAACCGGCGACGACATGCGC
GAAGAAGAGGACATGGCCAAGCGCCGTTCGCGCCTGAAGGCGTTGATCACGCTCGGCAAG
ACCCGCGGCTACCTGACGCAGGTCGAGATCTCCGACCACCTGCCCGACAAGCTGGTCGAC
GCCGAGACGATGGAAGTCGTGATCGCCATGTTGAACGACATGGGCATCGCGGTCTACGAG
CAGACGCCCGACGCCGAGACGCTGTTCCAGAACAACGTCACCCCCACCGCCGCCACTGTC
GAGGAAGCCGAGGAAGAAGCCGAGGCCGCGCTCTCCACCGTCGACAGCGAATTCGGCCGC
ACCACCGACCCGGTCCGCATGTACATGCGCGAAATGGGCACCGTCGAGCTGCTGACCCGC
GAAGGCGAGATCGAGATCGCCAAGCGCATCGAAGGCGGCCTGCAGGCCATGATGGAGGCG
ATCAGCGCGTCGCCCGCCACCATCGCCGCGATCCTGGTGATGGGCGAGGACATCCGCGAA
GGCAAGGTCGTCATCTCGACCATCGTCGACGGCTTCTCCAACCCCAACGAAGCCGACGAC
TACGTGGCCGAGGAGGACTTCGACGAGTTCGACGAGGAAGACGACGACGACGGCAAGGGC
GGCTCCAAGGCGCTGACCAAGAAGCTGGAAGAATTGAAGCGCGAGGCGCTGTCGCGCTTC
GAC
>CFBP5821 Xylophilus ampelinus
CACGCCGGCGGCAAGTTCAACCAGAACAGCTACAAGGTCTCGGGCGGCCTGCACGGCGTG
GGCGTGAGTTGCGTCAACGCGCTCAGCAAGATGCTGCGCCTGACGGTGCGGCGCGACGGC
AAGGTCCACGTGCTGGAGTTCAGCAAGGGCTTCGTGCAGAACCGCATCGTCGAGCAGGCG
GGCGGCGTCGAGGTCTCGCCGATGCGCATCATGGGCGACACCGAGAAGCGCGGTACCGAG
GTGCACTTCCTGCCCGACAACGAGATCTTCAAGGAAAACGCCGATTTCCACTACGAGATC
CTGGCCAAGCGCCTGCGCGAACTGAGCTTCCTGAACAACGGCGTGCGCATCCGCCTGAAG
GACGAGCGCAGCGGCAAGGAAGACGATTTCTCCGGCGCCGGCGGCGTGCAGGGTTTCGTC
GAATTCATCAACAAGGGCAAGACCATCCTGCACCCCACGCCCTTCTACGCCGCGGGCGAG
AAGCCGGCCGAGACGTACGGCGGCATCCCCGGCACGCACATCGGCGTGGAAGTGTCGATG
CAGTGGAACAGCGGCTACACCGAGCAGGTGCTCTGTTTCACCAACAACATCCCGCAGCGC
GACGGCGGCACCCACCTGACCGGCCTGCGCGCCGCGATGACCCGGATCATCAACAAGTAC
ATCGAAGAAAACGAGCTGGCCAAGAAGGCCAAGGTCGAGGTAACCGGCGACGACATGCGC
GAAGAAGAGGACATGGCCAAGCGCCGTTCGCGCCTGAAGGCGTTGATCACGCTCGGCAAG
ACCCGCGGCTACCTGACGCAGGTCGAGATCTCCGACCACCTGCCCGACAAGCTGGTCGAC
GCCGAGACGATGGAAGTCGTGATCGCCATGTTGAACGACATGGGCATCGCGGTCTACGAG
CAGACGCCCGACGCCGAGACGCTGTTCCAGAACAACGTCACCCCCACCGCCGCCACTGTC
GAGGAAGCCGAGGAAGAAGCCGAGGCCGCGCTCTCCACCGTCGACAGCGAATTCGGCCGC
ACCACCGACCCGGTCCGCATGTACATGCGCGAAATGGGCACCGTCGAGCTGCTGACCCGC
GAAGGCGAGATCGAGATCGCCAAGCGCATCGAAGGCGGCCTGCAGGCCATGATGGAGGCG
ATCAGCGCGTCGCCCGCCACCATCGCCGCGATCCTGGTGATGGGCGAGGACATCCGCGAA
GGCAAGGTCGTCATCTCGACCATCGTCGACGGCTTCTCCAACCCCAACGAAGCCGACGAC
TACGTGGCCGAGGAGGACTTCGACGAGTTCGACGAGGAAGACGACGACGACGGCAAGGGC
GGCTCCAAGGCGCTGACCAAGAAGCTGGAAGAATTGAAGCGCGAGGCGCTGTCGCGCTTC
GAC
>CFBP1192T Xylophilus ampelinus
CACGCCGGCGGCAAGTTCAACCAGAACAGCTACAAGGTCTCGGGCGGCCTGCACGGCGTG
GGCGTGAGTTGCGTCAACGCGCTCAGCAAGATGCTGCGCCTGACGGTGCGGCGCGACGGC
AAGGTCCACGTGCTGGAGTTCAGCAAGGGCTTCGTGCAGAACCGCATCGTCGAGCAGGCG
GGCGGCGTCGAGGTCTCGCCGATGCGCATCATGGGCGACACCGAGAAGCGCGGTACCGAG
GTGCACTTCCTGCCCGACAACGAGATCTTCAAGGAAAACGCCGATTTCCACTACGAGATC
CTGGCCAAGCGCCTGCGCGAACTGAGCTTCCTGAACAACGGCGTGCGCATCCGCCTGAAG
GACGAGCGCAGCGGCAAGGAAGACGATTTCTCCGGCGCCGGCGGCGTGCAGGGTTTCGTC
GAATTCATCAACAAGGGCAAGACCATCCTGCACCCCACGCCCTTCTACGCCGCGGGCGAG
AAGCCGGCCGAGACGTACGGCGGCATCCCCGGCACGCACATCGGCGTGGAAGTGTCGATG
CAGTGGAACAGCGGCTACACCGAGCAGGTGCTCTGTTTCACCAACAACATCCCGCAGCGC
GACGGCGGCACCCACCTGACCGGCCTGCGCGCCGCGATGACCCGGATCATCAACAAGTAC
ATCGAAGAAAACGAGCTGGCCAAGAAGGCCAAGGTCGAGGTAACCGGCGACGACATGCGC
GAAGAAGAGGACATGGCCAAGCGCCGTTCGCGCCTGAAGGCGTTGATCACGCTCGGCAAG
ACCCGCGGCTACCTGACGCAGGTCGAGATCTCCGACCACCTGCCCGACAAGCTGGTCGAC
GCCGAGACGATGGAAGTCGTGATCGCCATGTTGAACGACATGGGCATCGCGGTCTACGAG
CAGACGCCCGACGCCGAGACGCTGTTCCAGAACAACGTCACCCCCACCGCCGCCACTGTC
GAGGAAGCCGAGGAAGAAGCCGAGGCCGCGCTCTCCACCGTCGACAGCGAATTCGGCCGC
ACCACCGACCCGGTCCGCATGTACATGCGCGAAATGGGCACCGTCGAGCTGCTGACCCGC
GAAGGCGAGATCGAGATCGCCAAGCGCATCGAAGGCGGCATGCAGGCCATGATGGAGGCG

ATCAGCGCGTCGCCCGCCACCATCGCCGCGATCCTGGTGATGGGCGAGGACATCCGCGAA
GGCAAGGTCGTCATCTCGACCATCGTCGACGGCTTCTCCAACCCCAACGAAGCCGACGAC
TACGTGGCCGAGGAGGACTTCGACGAGTTCGACGAGGAAGACGACGACGACGGCAAGGGC
GGCTCCAAGGCGCTGACCAAGAAGCTGGAAGAATTGAAGCGCGAGGCGCTGTCGCGCTTC
GAC
>CFPB3232T Acidovorax anthurii
CACGCGGGCGGCAAGTTCAACCAGAACAGCTACAAGGTCTCGGGCGGCCTGCACGGCGTG
GGCGTCTCGTGCGTGAACGCGCTCAGCAAGATGCTGCGCCTCACCGTGCGCCGCGAGGGC
AAGGTGCACGTGCTCGAATTCAGCCAGGGTTTCGTGCAGAACCGCATCCTGGAGACGGTG
AACGGCGTCGAGGTCTCGCCCATGCGCGTGACCGGCGAGACCGACAAGCGCGGCACCGAG
GTGCACTTCCTGCCCGACACCGAGATCTTCAAGGAGAACCACGACTTCCACTACGAGATC
CTCGCCAAGCGCCTGCGCGAGCTCTCGTTCCTGAACAACGGCGTGCGCATCCGCCTGAGG
GACGAGCGCACGGGCAAGGAAGACGACTTCTCCGGCGCCGGCGGCGTGATGGGCTTCGTG
CAGTTCATCAACGCCAGCAAGAAGGTGCTGCACCCCAACGCCTTCCACGCCACGGGCAGC
CGCCCGGCCGAGACCTACGGCGGCATCCCCGGCACCGAGATCGGCGTGGAAGTGGCCATG
CAGTGGAACGACGGCTTCAACGAATCCGTGCTCTGCTTCACCAACAACATCCCGCAGCGC
GACGGCGGCACCCACCTCACGGGCCTGCGCGCGGCCATGACGCGCGTCATCGGCAAATAT
ATCGAGCAGAACGAGCTGGCCAAGAAGGCCAAGGTCGAGGTGAGCGGCGACGACATGCGC
GAAGAGGAAGACCTGGCCAAGCGCCGTTCCCGCCTGAAGGCCCTGATCACGCTGGGCAAG
ACCCGCGGCTACCTCACGCAGGTCGAGATCTCCGACCACCTGCCCGACAAGCTCGTCGAT
GCCGAGACCATGGAAGTCGTGGTCACCATGCTCAACGACATGGGCGTGGCCGTGTACGAG
CAGACGCCCGACGCCGAGACGCTGTTCCAGAACAACGTGACGCCCACGGCCACCACCGTG
GAAGAGGCCGAGGAAGAAGCCGAGGCCGCGCTCTCCACCGTGGACAGCGAATTCGGCCGC
ACCACCGACCCCGTGCGCATGTACATGCGCGAGATGGGCACGGTGGAGCTGCTCACGCGC
GAGGGCGAGATCGAGATCGCCAAGCGCATCGAAGGCGGCCTGCAGGCCATGATGGAAGCC
ATCAGCGCGTCTCCCGCCACCATCGCCGAGATCCTCGCCATGGGCGAGGAGATCCGCGAG
GGCAAGGTCGTCATCTCCACCATCGTGGACGGCTTCTCCAACCCCAACGAGGCCGATGAC
TACGTGGCCGAGGAAGACTTCGACGAGTTCGACGAAGAGGACGACGACGACGGCAAGGGC
GGCTCCAAGGCCCTCACCAAGAAGCTGGAAGAACTCAAGAACGAGGCCCTGCGCCGCTTC
GAC
